# Uganda chicken genetic resources: I. phenotypic and production characteristics

**DOI:** 10.3389/fgene.2022.1033031

**Published:** 2023-01-24

**Authors:** Illyass Yussif, Donald Rugira Kugonza, Marion Wilfred Okot, Peace Oliver Amuge, Rosa Costa, Filomena Dos Anjos

**Affiliations:** ^1^ College of Agricultural and Environmental Sciences, Makerere University, Kampala, Uganda; ^2^ Faculty of Agriculture and Environment, Gulu University, Gulu, Uganda; ^3^ Women of Uganda Network, Kampala, Uganda; ^4^ Kyeema Foundation, Maputo, Mozambique; ^5^ Faculty of Veterinary Medicine, Eduardo Mondlane University, Maputo, Mozambique

**Keywords:** indigenous chickens, qualitative traits, Gallus gallu, descriptive phenotypic traits, scavenging management system, phenotypic effect on morphological traits, genetic diversity, chicken strains

## Abstract

The extent of diversity in the indigenous chicken breeds of Uganda was assessed for their potential utilisation in breeding programmes. A total of 293 indigenous-chicken-keeping households in villages across 35 districts forming 12 sub-regions of the four regions were randomly sampled for 586 mature chickens of both sexes. While only 20.8% of households were headed by women, 40.6% of indigenous chicken keepers were women. The production objectives mainly focused on chickens as sources of income from sales and household food. The chickens were predominantly managed in a scavenging (94.2%) feeding system in a mainly (96.9%) mixed crop-livestock system. The average flock size was 19.80 ± 1.21 chickens with 3.83 ± 0.29 laying hens, each producing an average of 13.41 ± 0.20 eggs/clutch and 40.3 ± 0.6 eggs/annum of 5.20 ± 0.03-month egg production age. Normal-feather strains predominated (>90%), with scattered incidences of naked neck, frizzles, polydactyl, and ptilopody traits in both sexes. Dark (49.0% hen; 43.8% cock) and white (38.3% hen; 42.4% cock) skin colours were most common among the chickens compared to yellow skin. However, yellow-coloured shanks were proportionally the most observed (41% cock; 29% hen). Orange and brown iris (eye) colours were the most common in both sexes. The hens commonly had small round earlobes with varying colours, while cocks had large oval-shaped, mainly red (70%) earlobes. The single-comb type was dominant in both sexes, with wattles almost universally present. Frizzle and polydactyl allele frequencies were significantly lower (*p <* 0.05) than the expected Mendelian proportions, indicating a possible state of endangerment. Meanwhile, the estimated allele frequencies of ptilopody, tufted-crest, and rose comb alleles in the population were similar (*p >* 0.05) to the expected Mendelian frequencies. However, these strains did not show any significant (*p >* 0.05) influence on the body weight or the linear morphometric estimates except for being marginally higher than the normal strains. The phenotypic correlations of body weight and morphometric traits ranged from 0.457 to 0.668 and 0.292 to 0.454 in cocks and hens, respectively. These findings provide hints about the prospects for improved performance with modifications in the production environment. The wide phenotypic diversity would support management efforts for their sustainable utilisation and preservation.

## Introduction

Domestic free-range/scavenging indigenous chickens sustain the livelihoods of millions of people in smallholder subsistence economies (FAO, 2018a). They are amongst the most important livestock species, constituting the most popular domesticated animal in Uganda to provide a regular source of meat (65,000 tonnes per year) and eggs to a large proportion of the population (FAO, 2019a). Over 85% of the national chicken flock population of Uganda are mainly indigenous breeds (FAO, 2019a; NEMA, 2019), traditionally kept by smallholder backyard poultry farmers under a free-range system (MAAIF & UBOS, 2009; Vernooij et al., 2018; FAO, 2019b; MAAIF, 2019). Together with beef, chicken has been targeted in the Uganda Agriculture Sector Strategic Plan 2015/16–2019/20, as a priority commodity for development (FAO, 2018b). Tropical production environments are challenging, and most farmers raise their domestic indigenous chickens under no to minimal input conditions. Resilience to selective pressures is, therefore, what has allowed chickens to remain predominant in many villages, with wide phenotypic variability (Dessie et al., 2011; FAO, 2015; Mpenda et al., 2018; FAO, 2010). This indicates a huge genetic diversity requiring comprehensive characterisation, inventorying, and monitoring across different agroecological zones for their sustainable utilisation and conservation under the prevailing production system. Moreover, in the wake of the impact of climate change already dawning on Africa more intense and frequent climate stressors are expected to increase, particularly in East Africa, by 2050 (Waithaka et al., 2013; Girvetz et al., 2019). The rapidity with which mitigating adaptive measures are instituted will ensure that we can cope with climate change in the region, especially with the ongoing introgression of exotic breeds into the indigenous population to improve their performance. Examples of these introgressions include the Serere Agriculture and Animal Production Research Institute (SAARI) exotic chicken crossbreeding project (Ssewannyana et al., 2019), the National Animal Genetic Resources Centre and Data Bank (NAGRC & DB) Kuroiler crossbreeding projects (USAID, 2017), the *Rakai* (district) local chicken improvement with Bovans White sires (Roothaert et al., 2011) as well as the *Namasagali* (the local hen in Namasagali town of Kamuli district, Eastern Uganda) and Kuroiler sires crossbreeding project by National Livestock Resources Research Institute (NaLIRRI)-Gulu-Makerere University (Kayitesi, 2015; RUFORUM, 2016).

Therefore, defining the genetic attributes among the indigenous chicken genetic resources and determining the state of their available diversity are useful for an effective national breeding programme. This would involve a systematic identification, inventory, monitoring, and description of the production environment to set the entry point to the sustainable utilisation and conservation of these animal genetic resources (AnGR) (FAO, 2012; AU-IBAR, 2019). Our study, therefore, aimed to assess the production characteristics, phenotypic diversity, occurrence, and performance of major chicken genotypes/strains in the indigenous chicken population of Uganda under traditional husbandry conditions. Our findings will provide preliminary geographic scope information regarding the phenotypic characteristics of the indigenous chickens in Uganda and a description of their production system, thus setting a basis for wider genetic diversity studies to identify valuable chicken genetic resources for the selection and improvement of breeding programmes to mitigate the unavoidable climate change.

## Materials and methods

### Study area and study period

**FIGURE 1:**
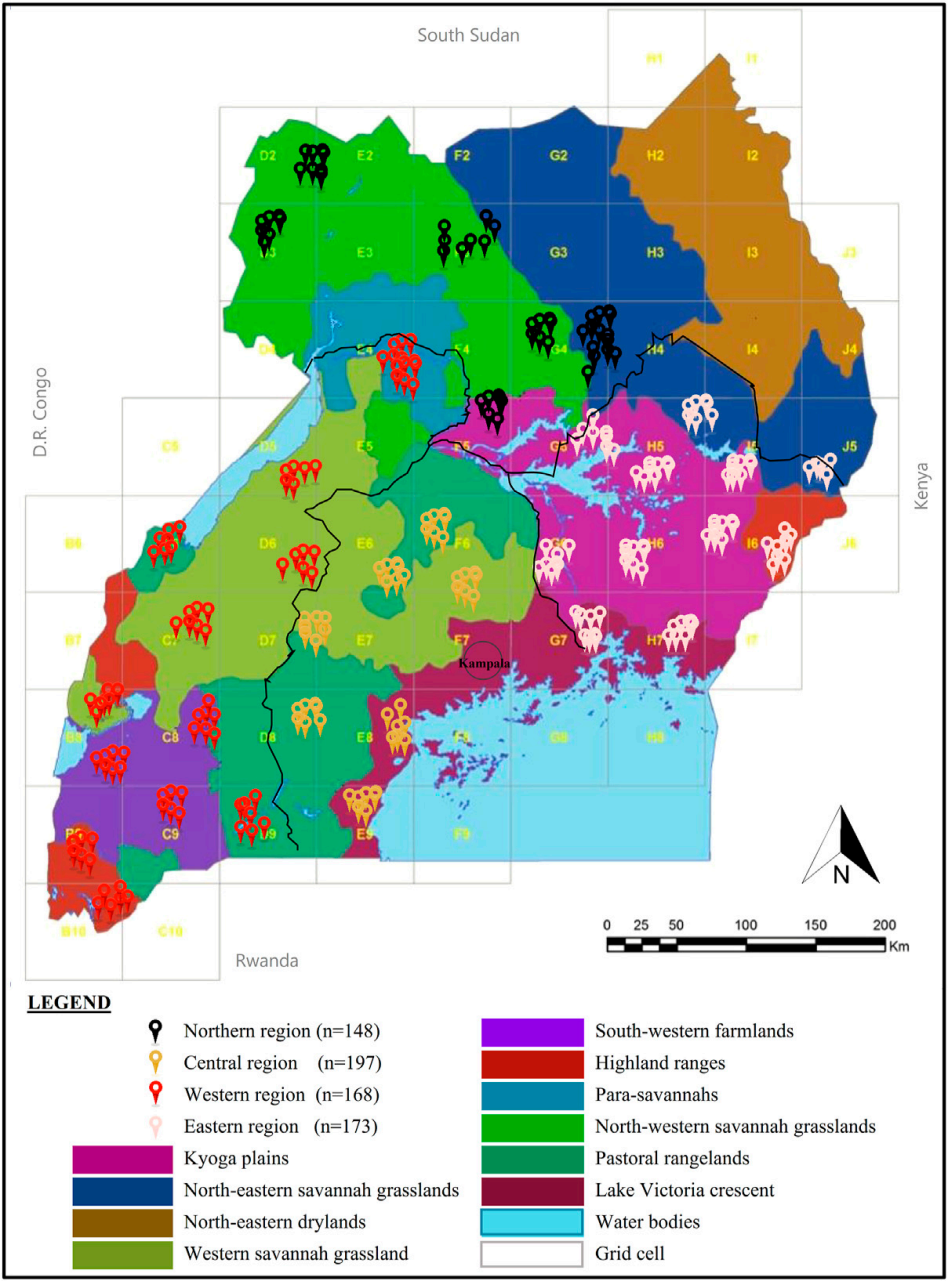
Map of the study locations of the indigenous chickens in Uganda. The sample distribution of the 293 randomly selected households from villages (>5 km apart) was obtained from grid cells of approx. 50 km^2^ across the AEZs of Uganda to ensure the collection of landscape data.

This study, covering 293 indigenous-chicken-keeping homesteads across 35 districts forming 12 sub-regions of the four regional clusters and spanning the diverse agricultural production/ecological zones (AEZs) of Uganda (Figure 1 and Supplementary Table S1), was conducted from January to March 2020. The Global Positioning System (GPS) map of the study location in Uganda is available as a Google map (here). Uganda is landlocked, bordered by the Congo Democratic Republic (DR), Kenya, Rwanda, South Sudan, and Tanzania. Much of its border is lakeshore and located astride the Equator (between latitudes 4^°^ North and 1^°^ South and longitudes 30^°^ East and 35^°^ East). The altitudes of the survey areas ranged from 614 to 2,261 m, averaging 1,184 m above mean sea level. Uganda has a total area of 241,550.7 km^2^ of which about 197,065.91 km^2^ is land area and 7,620.76 km^2^ is swamp and inland water masses. Although generally equatorial, the climate is not uniform since the altitude modifies the climatic conditions and vegetation type. Hence, 10 AEZ exist, defined by similar ecological conditions and socioeconomic characteristics, farming systems, and practices (MAAIF, 2010). A unique AEZ has common crops and livestock types while zones cut across districts (Kraybill and Kidoido, 2009). The descriptions of the AEZs are shown in Supplementary Table S2. The Northeastern drylands AEZ was excluded from this study because the area is a semi-arid zone inhabited by nomadic pastoralists who derive their livelihoods from cattle keeping and so hardly keep chickens. Additionally, the subregion (Karamoja) is prone to insecurity due to cattle rustling.

### Study design and data collection

**FIGURE 2:**
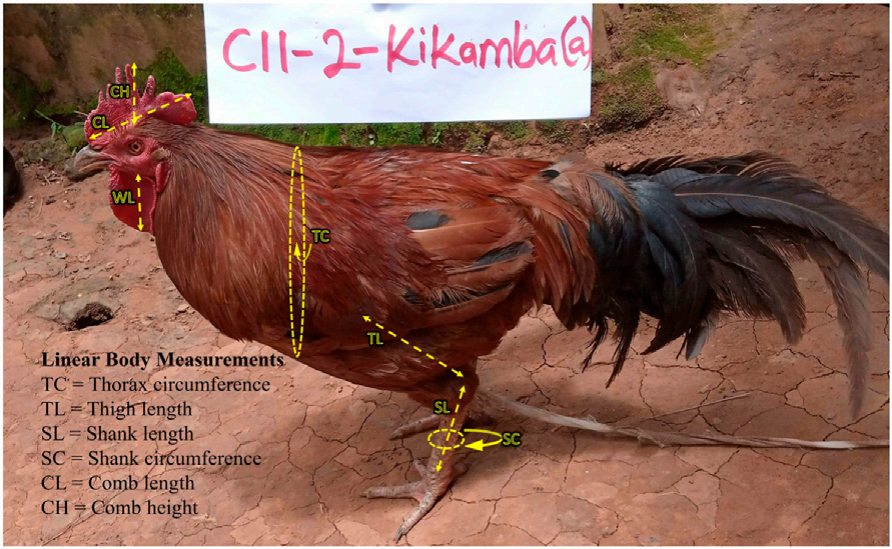
Linear measurements (in cm) of the indigenous chickens in Uganda, as described by AU-IBAR (2015b).

Chickens were sampled mainly from rural households based on grid cells of 50 km^2^ across the Ugandan landscape map (Figure 1). The wide landscape sampling ensured varied agro-climates, which have implications for genetic diversity through adaptive divergence. As such, sampling genetically diverse populations was maximised by randomly selecting at least three villages separated by at least 5 km in the grid-cell-identified districts. Then, with the help of the District Veterinary Officers (DVOs), reconnaissance surveys were conducted to sample one indigenous chicken-keeping household from each village for the study. Data on a total of 293 farmer-households and their flock profiles with production history were obtained through interviews (in the language they understood) using the African Union-InterAfrican Bureau for Animal Resources (AU-IBAR) harmonised standard questionnaire, the AnGR-CIM Tool ENFR v2.1 (deployed on the ODK Collect v1.29.5 application for Android devices). Following the AnGR guidelines for phenotypic characterisation (FAO, 2012), two mature unrelated chickens (a hen and a cock) were used for the phenotypic study in each of the farmer-households, totalling 586 (299 hens: 287 cocks) birds across sites. Pictorial field guidebooks (AU-IBAR, 2015a) aided in the description of the qualitative phenotypic characteristics regarding body condition score, body colours (skin, shank, eyes, ear lobe, and beak); plumage and feathering features (feather structure, feather distribution, and body plumage pattern); head features (earlobe size, earlobe shape, comb type, wattles, and crests and beak shapes); and skeletal variance (body shape/conformation, frame, spur size, and tail length). Body weights (in grams) were measured using a standard electronic weighing scale (WH-A08; 0–10 kg) while the linear body measurements of the live chickens (Figure 2) were taken using a tailors tape measure. The GPS location of each sample homestead and digital photographs of the studied chickens were captured with the integrated AnGR-Photo Tool (ENFR v2.1). The ages of the chickens were obtained from farmers’ records or estimated (where records were unavailable) as recalled by the owner or judged by visual appraisal as in the AnGR guidelines for phenotypic characterisation (FAO, 2012) and described by Birteeb et al. (2016). The body conditions of most of the matured cocks and hens were scored as emaciated, thin, good, or fat, on a scale of 1, 2, 3, and 4, respectively.

Estimation of the effective population size (Ne) for the randomly mated indigenous chicken populations across the household flocks in each region and their corresponding rate of inbreeding (∆F) were executed as described by Falconer and Mackay (1989) using the equations:

Effective population; $Ne=\frac{4\left(N_{m}\times N_{f}\right)}{N_{m}+N_{f}}$

Rate of inbreeding; $\Delta f\frac{1}{2N_{e}}$

where N_e_ is the net effective population size, N_m_ is the number of breeding cocks in the flock, N_f_ is the number of breeding hens in the flock, and ΔF is the rate of inbreeding per generation.

Records of indigenous chicken strains showing major genes that characterise genotypes with known adaptive values like the allele for frizzling [F/f], ptilopody [Pti/pti], tufted crests [Cr/cr], rose comb [R/r], and Polydactyl [P/p] were used to estimate their allele frequencies. The proportions of phenotypic counts were computed as:

$$Phenotypic\;frequency=\frac{Number\;of\;individuals\;carrying\;trait\;}{Total\;number\;of\;individuals\;sampled}\times 100$$

Chi-squared goodness of fit analyses of the proportions of the observed chicken strains against the expected Mendelian proportions (25% and 75%, respectively for incidence and absence of the genotype) were conducted using the chisq.test method in R software. The estimates of allele frequencies using the Hardy-Weinberg principle (Falconer and Mackay, 1989) were based on the models below:

$$q=\sqrt{\frac{m}{t}}\;and\;p=1\;–\;q$$

where q is the frequency of recessive allele, m is the observed number of indigenous chickens expressing the recessive phenotypes under consideration, t is the total number of chickens sampled, and p is the frequency of dominant allele expressed in the chickens not showing the major trait considered.

### Statistical analysis

With sample locations (geographical regions) and chicken sex categories fitted as fixed independent variables, the dependent variables were subjected to descriptive-analytical procedures in IBM SPSS Statistics for Windows, version 20.0.0.0. Kendall’s concordance coefficient *W* tests were applied to test and rank agreement among farmers regarding their rated household chicken production objectives at a 5% significance level. Analysis of variance (ANOVA) was conducted using the general linear models in IBM SPSS Statistics for Windows, version 20.0.0.0. The least-square means of significant differences were compared using Scheffe’s adjustment to account for unequal sample sizes per category. The model for the morphometric traits (body weights and linear body measurements), which excluded the interaction effects because they were non-significant, took the form: $Y_{ijk\;}=µ+{\;l}_{i}+s_{j}+e_{ijk}$ _;_ where $Y_{ijk\;}$ is the observation of the morphometric trait in chicken location *i*, for chicken sex *j*, µ is the overall mean, l_i_ is the effect of location (*i* = Northern, Central, Western, or Eastern region); s_j_ is the effect of sex (*j* = hen or cock), and e_ijk_ is the random effect. The overall correlation among these traits was tested using Pearson correlations.

## Results

### Demographic profile of indigenous chicken-rearing households in Uganda

The demographic analysis of the indigenous chicken-keeping households in Uganda, presented in Table 1, showed relatively similar proportions of male and female respondent farmers (*p >* 0.05) across the regions. While male respondents were mostly higher (*p* < 0.001) in Northern (64.9%), Western (56.0%), and Eastern (66.3%) Uganda, the opposite was true for Central Uganda, where female respondents (55.1%) were observed. The responses were largely (*p* < 001) obtained from the household heads (60.1%), followed by the spouses of the household heads (28.0%), with only a few coming from other household members in similar (*p >* 0.05) proportions across the regions. Most households (*p <* 0.001) were male-headed (79.2%), with significantly (*p* < 0.01) different proportions across the regions. However, the aggregate proportion of male respondents, was only slightly higher (59.4%) across the regions, in a society where most households are headed by men (*p* < 0.001). The age composition of the respondents was characteristically similar to the population pyramid in most developing countries, in which the active working group (30–60-year-olds) accounted for most (74.4%) of the respondents against those aged below 30 down to 22 (8.9%) or above 60 years (16.7%) in this study. The mean age across regions was similar (*p >* 0.05), with an overall mean age of 47.3 ± 0.8 years (range: 22–88 years). The average household sizes were also comparable (*p >* 0.05) across regions, with an overall mean size of 7.6 members. The mean distance from the farmers’ homestead to the market was 5.6 km and to an all-weather road was 33.3 km, both of which were similar (*p >* 0.05) across regions. The proportions of the most important household income source were similar (*p* > 0.05) across regions. In most households, the sale of crop (48.8%) and livestock and livestock-products (14.7%) respectively formed the most significant economic activity, which provided incomes for most families (*p* < 0.001). Only a few (12%) indigenous chicken-keeping families derived some income from off-farm activities or the trading of livestock and livestock-products. Some (27.3%) of households did not disclose information regarding their income source, likely due to a belief that one loses wealth upon disclosure, especially among farmers in western and central Uganda.

**TABLE 1:** Demographic analysis of indigenous chicken farmers in Uganda.

Variable (n = 293)	Farmer-households [n (%)]					χ^2^, region	χ^2^, category	*p*-value^1^
	Northern n = 74	Central n = 49	Western n = 84	Eastern n = 86	Total n = 293			
***Gender of respondents*** (***farmers***)						7.29^ns^	10.32^**^	
Male	48 (64.9)	22 (44.9)	47 (56.0)	57 (66.3)	174 (59.4)			
Female	26 (35.1)	27 (55.1)	37 (44.0)	29 (33.7)	119 (40.6)			
***Position of the respondent within the household***						4.85^ns^	105.55^**^	
Household head	48 (64.9)	28 (57.1)	46 (54.8)	54 (62.8)	176 (60.1)			
Spouse	20 (27.0)	12 (24.5)	26 (31.0)	24 (27.9)	82 (28.0)			
Other-household member	6 (8.1)	9 (18.4)	12 (14.3)	8 (9.3)	35 (11.9)			
***Gender of household head***						15.38^**^	99.80^***^	
Male	67 (91)	31 (63)	70 (83)	64 (74)	232 (79.2)			
Female	79)	18 (37)	14 (17)	22 (26)	61 (20.8)			
***Age of respondents*** (***farmers***)						3.4^ns^	225.10^***^	
22–30 years	10 (13.5)	3 (6.1)	5 (6.0)	8 (9.3)	26 (8.9)			
31–60 years	52 (70.3)	38 (77.6)	64 (76.2)	64 (74.4)	218 (74.4)			
Over 60 years	12 (16)	8 (16.3)	15 (17.9)	14 (16.3)	49 (16.7)			
Age (*Mean ± SE*)	46.6 ± 1.6	48.0 ± 1.6	48.7 ± 1.4	46.2 ± 1.4	47.3 ± 0.8			0.577
Household size (*Mean ± SE*)	7.91 ± 0.56	8.24 ± 0.67	7.68 ± 0.37	7.22 ± 0.46	7.64 ± 0.27			0.628
***Average distance from farmers’ homestead*** (***Mean±SE***)								
Road^1^ (km)	1.16 ± 0.29	4.57 ± 1.02	2.50 ± 0.35	5.31 ± 4.65	33.30 ± 1.38			0.691
Market (km)	3.16 ± 0.26	6.58 ± 0.93	4.28 ± 0.41	8.45 ± 4.64	5.60 ± 1.38			0.497
***The most important household income of source*** (***n = 213***)						7.74^ns^	217.55^***^	
Crop products sales	53 (71.6)	13 (26.5)	20 (23.8)	57 (66.3)	143 (48.8)			
Sale of L&LP*	11 (14.9)	8 (16.3)	10 (11.9)	14 (16.3)	43 (14.7)			
Trade in L&LP	1 (1.4)	-	-	1 (1.2)	2 (0.7)			
Off-farm	9 (12.2)	2 (4.1)	3 (3.6)	11 (12.8)	25 (8.5)			
Undisclosed	-	26 (53.1)	51 (60.7)	3 (3.5)	80 (27.3)			

Numbers in brackets are the percentages of total respondents in each location; n = chickens sampled (-) = not reported; * livestock and livestock-products; χ^2^ = chi-square tests for among region and within variable categories; ^ns^ *P*>0.05, ^*^ *p* < 0.05, ^**^ *p* < 0.01, ^***^ *p* < 0.001; ^1^ *p*-value of one-way ANOVA; SE, standard error. Means with no superscripts within rows did not differ significantly (*p* > 0.05). ^1^Distance from household to an all-weather road.

### Flock descriptions, ownership, and production objectives of indigenous chickens in Uganda

The number and proportion of the different chicken age and sex categories in a flock describe the structure of the flock. The results presented in Table 2 and Supplementary Figure S1A–D show that all households kept indigenous chickens of different flock compositions and ownership characteristics. The composition of flocks varied (*p* < 0.01) with the regions and the lowest flock numbers per category were in Central and Western Uganda whilst the highest were observed in Northern and Eastern Uganda. An overall average of 19.80 ± 1.21 chickens was held in indigenous-chicken-keeping households. Similarly, the flock size per chicken category differed significantly (*p <* 0.001) with the proportions of households owning them. Most households held between one and five chickens for the different flock compositions, except for chicks that were also held substantially in all sizes by relatively larger proportions of households. Cock numbers were mainly low, between one to five in most (92.8%) households and across the regions, with an average of 2.6 ± 0.2. Up to 10 hens and growers (pullets/cockerels) were kept in most households, with averages of 6.9 ± 0.4 hens, 4.8 ± 0.5 cockerels, and 5.5 ± 0.4 pullets, respectively. In most households, adult men owned the highest (37.4%) number of chickens in the flock compared to adult women (24.6%) Joint ownership by adult men and women household members was also common (28.6%), whilst children under 18 years owned just a few chickens in a flock. In rare instances, a few chickens were kept elsewhere from the household flock or were not owned but kept on behalf of others (Supplementary Figure S1A). The overall effective population size (*Ne*) and rate of inbreeding (ΔF) estimated for the indigenous chicken flock kept across the farmer-households were, therefore, 2,200 and 0.023%, respectively (Supplementary Figure S1B). Regionally, the net effective population size was higher in the Eastern region (802), followed by the Northern (636), Western (441), and Central regions (313), respectively; with a corresponding trend in the rate of inbreeding, which was lower in the Eastern (0.062%) and Northern regions (0.079%) but higher in the Central (0.160%) and Western regions (0.113%). The selling price of chickens differed (*p* < 001) with age category (Supplementary Figure S1C) with the highest average price observed for mature cocks (UGX 24,110.8/ = ∼ US$ 6.42). The average price of a mature hen (UGX 16,576.27/ = ∼ US$ 4.48) was similar to that of a cockerel (UGX 16,421.05/ = ∼ US$ 4.44) and was lowest for a pullet (UGX 9,555.56/= (US$ 2.58). The purpose of selling chickens among the farmers varied significantly (*p* < 0.01) with only 47.6% degree of concordance. However, selling chickens to meet planned household expense was ranked first by the majority (69.2%) of households (Supplementary Figure S1D). The body condition scores of most of the breeding cocks and hens were similar (*p >* 0.05) across households (Supplementary Figure S2). Few thin (7.2% cocks; 8.9% hens), fat (2.4% cocks; 1.7% hens), or emaciated (0.3% cocks; 1.7% hens) chickens were observed, with most of the cocks (90.1%) and hens (87.7%) showing good body condition. Flock numbers showed an increasing trend among farmers with smaller flock sizes when analysed retrospectively across 12 calendar months (Supplementary Figure S3).

**TABLE 2:** Composition of household indigenous chicken flocks in Uganda.

Flock description	Farmer-households					*p*-value
	Northern	Central	Western	Eastern	Overall	
*Flock composition (Mean ± SE)*						
Breeding Cock	3.1 ± 0.4^a^ ^,^ ^b^	2.1 ± 0.2^a^ ^,^ ^b^	1.8 ± 0.2^a^	3.2 ± 0.4^b^	2.6 ± 0.2	0.002
Breeding hens	7.0 ± 0.6^a^ ^,^ ^b^	7.1 ± 1.5^a^ ^,^ ^b^	4.8 ± 0.5^a^	8.9 ± 0.9^b^	6.9 ± 0.4	0.004
Cockerels	7.6 ± 0.8^a^	1.9 ± 0.7^b^	1.5 ± 0.2^b^	7.5 ± 1.3^a^	4.8 ± 0.5	<0.001
Pullets	9.0 ± 1.2^a^	3.0 ± 0.8^b^	2.1 ± 0.3^b^	7.0 ± 0.8^a^	5.5 ± 0.4	<0.001
Chicks	17.7 ± 2.4^a^	8.7 ± 1.3^b^ ^,^ ^c^	6.5 ± 0.8^c^	15.4 ± 1.7^a^ ^,^ ^b^	12.3 ± 0.9	<0.001
Total flock size	26.8 ± 2.6^a^	14.0 ± 2.3^b^	10.1 ± 0.8^b^	26.6 ± 2.7^a^	19.8 ± 1.2	<0.001
***Flock category size, n = 293*** (***%***)	**1–5 chickens**	**6–10 chickens**	**11–15 chickens**	**16–20 chickens**	**20+ chickens**	**χ** ^**2**^
Cock	272 (92.8)	15 (5.1)	3 (1.0)	2 (0.7)	1 (0.3)	973.60^**^
Hens	169 (57.7)	87 (29.7)	17 (5.8)	7 (2.4)	13 (4.4)	332.20^**^
Cockerels	216 (73.7)	42 (14.3)	12 (4.1)	14 (4.8)	9 (3.1)	504.46^**^
Pullets	191 (65.2)	64 (21.8)	18 (6.1)	9 (3.1)	11 (3.8)	408.42^**^
Chicks	99 (33.8)	72 (24.6)	48 (16.4)	28 (9.6)	46 (15.5)	51.52^**^

^a^ Means within rows with different superscripts indicate significant differences (*p* < 0.05).

^b^ SE, standard error of means.

^c^ Percentages (in brackets) are based on the total number of respondents (household) per each category in rows. χ^2^ = chi-square test.^**^ *p* < 0.01; ^**^ *p* < 0.001.

They are the flock size range (as heading) per category of chickens kept.

Chicken exit from the flock showed different forms among indigenous chicken-producing households in Uganda. While most households allowed cocks to stay in the flock for up to 1 year (41%) or longer (44%), only about 15% of households kept their cocks for just a few months (Supplementary Figure S4). One-third (29%) of farmers exchanged their chickens to acquire other livestock species (Supplementary Figure S5), usually (25.0%) goats but also occasionally pigs (1.4%), chickens with unique traits (1.45%), cattle (1.0%), and sheep (0.3%).

The production objectives of indigenous chickens among households across Uganda were significantly (*p <* 0.001) similar. The farmers were 70.1% concordant with one another regarding the ranked purposes for keeping chickens (Table 3). The most significant production objectives of indigenous chickens, ranked first and second across the regions, were income generation from the sale of the chickens and consumption as food in the form of meat and eggs, respectively. The lowest ranked production objectives included the benefits obtained from by-products in the form of chicken droppings for use as organic fertilisers in their crop gardens, socio-economic/prestige and cultural reasons, and keeping chickens for gifts to visitors and/or barter trading to obtain other livestock species and for leisure purposes, respectively.

**TABLE 3:** Reasons/purposes for keeping chickens.

Ranking variables (n = 293)	Household ranking^a^					Total (%)	Mean ranks
	1st	2nd	3rd	4th	5th		
*Purpose of keeping chickens*							
Income (cash from sales)	91	115	11	-	-	217 (45.5)	5.40
Food (meat and egg source)	130	72	4	-	-	206 (43.2)	5.02
By-products (dropping)^b^	1	12	8	2	1	24 (5.0)	2.81
Socio-economic/prestige and Culture	1	7	9	4	-	21 (4.4)	2.77
Gift/barter^c^	1	2	2	1	-	6 (1.3)	2.53
Leisure	1	-	1	1	-	3 (0.6)	2.47
Kendall’s W^d^						0.701	
Chi-square test (χ^2^)						782.12^**^	

^a^ The purposes for keeping chickens in households were ranked from 1 (most) to 5 (least) in corresponding order of importance.

^b^ By-product (droppings) for use as organic fertiliser.

^c^ Gifts/slaughtered for visitors or exchanged for other livestock species.

^d^ Kendall’s coefficient of concordance W. % = relative proportion, (-) = not reported. ^**^ *p* < 0.001.

### Indigenous chicken production/management systems and their performance in Uganda

#### Feed resources, feeding and watering management practices

The production system and feeding management practised were similar across the regions (*p* > 0.05). The mixed crop-livestock system, where farmers kept some livestock alongside crop farming, was the predominant (96.9%) agricultural production system in most farmer-households (Table 4). However, the agro-pastoral system (1.8%) which combines crop and pastoral livestock production and the farming of only poultry species under scavenging management (1.3%) formed isolated cases of production systems in households in the Northern, Central, and Western regions. Across the regions, scavenging (free-range) feeding management was mainly (94.2%) practised in most indigenous-chicken-keeping households, irrespective of the season, followed by restrictive scavenge-feeding (1.3%), where the flock only has access to leftovers, brans or grains within a confined area; or scavenging-supplementation (2.2%), where the flock is provided supplemental feed as they scavenge for feed near and around the homestead (Supplementary Figure S6). Hardly any formulated feed (0.4%) balanced for all the required nutrients was provided to the chickens in most households except in one case each from the Northern, Western, and Eastern regions.

**TABLE 4:** Indigenous chicken production and management practices in Uganda.

Production and management practices	Farmer-households, n (%)				Total	χ^2^
	Northern	Central	Western	Eastern		
***Production system*** (***dry or wet season***)						11.833^ns^
Agro-pastoral	2 (2.7)	2 (7.7)	-	-	4 (1.8)	
Mixed crop-livestock	70 (94.6)	24 (92.3)	36 (97.3)	86 (100)	216 (96.9)	
Scavenging-poultry^a^	2 (2.7)	-	1 (2.7)	-	3 (1.3)	
Total	74	25	37	86	223	
***Feeding management practised***						13.394^ns^
Scavenging (free-range)	71 (95.9)	25 (96.2)	31 (83.8)	86 (96.5)	214 (94.2)	
Restrictive scavenging feeding^b^	2 (2.7)	-	1 (2.7)	1 (1.2)	3 (1.3)	
Scavenging-supplementation^c^	-	1 (3.8)	4 (10.8)	1 (1.2)	5 (2.2)	
Nutritionally complete feeding^d^	1 (1.4)	-	1 (2.7)	1 (1.2)	1 (0.4)	
Total	74	26	37	86	223	
***Feed available to chickens*** ^e^						71.138^***^
Concentrate (*grains*)	31 (53.4)	-	-	28 (46.6)	59 (11.7)	
Pasturage (*forage, seeds, worms*)	46 (29.3)	25 (16.6)	36 (22.9)	50 (31.2)	157 (31.6)	
Agro-by-product (*spent grains/bran*)	18 (12.9)	23 (17.3)	25 (20.1)	68 (49.6)	133 (28.0)	
Kitchen residue (*leftovers*)	43 (31.2)	24 (16.7)	30 (22.5)	40 (29.7)	137 (27.8)	
Nutritionally complete feed^d^	1 (20.0)	-	1 (20.0)	3 (60.0)	5 (1.0)	
***Water provision to chicken flocks***						71.141^***^
Water is fetched/provided	69 (93.2)	25 (51.0)	36 (42.9)	76 (88.4)	206 (70.3)	
Chicken searches for water	5 (6.8)	24 (49.0)	48 (57.1)	10 (11.6)	87 (29.7)	
Total	74	49	84	86	293	
***Quality of water available to chickens***						100.622^***^
Muddy	2 (2.7)	24 (49.0)	46 (54.8)	1 (1.2)	73 (24.9)	
Good/clear	72 (97.3)	25 (51.0)	38 (45.2)	85 (98.8)	220 (75.1)	
Total	74	49	84	86	293	

^a^ Production of only poultry species under scavenging management.

^b^ Chickens are only fed leftovers, grains, brans, etc. within a tethered radius.

^c^ Scavenging with some supplementation.

^d^ Chickens fed manufactured or formulated feed balanced for all nutrients.

^e^ Feed available row percentages are based on the sum of responses for each category. The row total percentages are based on the overall responses (i.e., households feed more than one type). Wet and dry = rainy and dry seasons. (-) = not reported. ^ns^ *P*>0.05; ^*^ *p* < 0.05;^**^ *p* < 0.001.

The three major feed resources available for the chicken flock were pasturage (31.6%) near or around the homesteads for the chickens to forage, followed by agricultural by-products like spent grains from breweries, residues and bran from grain mills (28.0%), and kitchen refuse/leftovers (27.8%). Only a small proportion of households (11.7%) provided grain concentrates to supplement their flock’s scavengeable feed resources, with rare cases of households (1.0%) providing manufactured or formulated nutritionally complete feed to their chickens. In most households in the Western region (57.1%) and a good number in the Central region (49.0%), chickens were not provided water for drinking; rather, the chickens searched or walked to sources of water within their scavenging range. However, most households in the Northern (93.2%) and Eastern (88.4%) regions provided drinking water to their flocks. The water sources within the scavenging radius of the flock were good in most (75.1%) of the households except for one-third of those in Central and Western Uganda, in which the water sources were muddy.

#### Housing systems for indigenous chickens in Uganda

Most households provided chickens with some form of housing to shelter both young and old birds (*p >* 0.05). Predominant among the housing units across the households were shelters constructed on the side of the main house (43.9% young; 46.9% adult) and sheds (43.4% young; 37.2% adult) in the homestead. A few other households sheltered their chickens in human dwellings (10.2%, young and adults)), off-ground perches/kitchens/trees (2.0% young; 4.6% adult), and stalls (0.5% young; 1% adult) (Supplementary Figures S7A, S8). Regional differences were observed in the housing structures of chickens (*p* < 0.001). Sheds were more popular in the Northern and Eastern regions whilst shelters constructed on the side of the main house dominated the Central and Western regions (Supplementary Figure S7B, S8). However, the proportions of households keeping chickens in human-dwelling units were similar across regions.

#### Chicken health services and disease control

Disease incidence and treatment dynamics in the chicken flock differed significantly (*p <* 0.001) across the households (Supplementary Table S3). Holistically, disease episodes in most household indigenous chicken flocks were reported to occur once in a while (35.5%) and seasonally (32.4%), as almost a quarter of the households (24.2%) reported seldom experiencing disease challenges. Most of the households reporting a low disease incidence were in the Central and Western regions, whereas households in Northern and Eastern regions mostly reported occasional disease episodes. Most farmer-households across the regions treated diseased chickens only when they were very sick. Farmers, especially those in Northern (45.9%) and Eastern (33.7%) regions, had to treat their diseased chickens, or they died of their ailment during the disease episodes. Many of the farmers reported that their chickens recovered on their own without treatment during disease episodes or died, while farmers in the Northern and Eastern regions reported losing their birds before any treatment could be administered. A small proportion of farmers across the regions did not encounter disease episodes in their flock, which they attributed to their use of phyto-prophylaxis.

Poultry health services available to chickens differed (*p* < 0.001) across regions, with curative treatment the most common (49.5%), followed by vaccination (45.3%) which was higher in the Northern (36.2%) and Eastern (48.5%) regions (Supplementary Table S3 and Supplementary Figure S9A). Antihelminthic deworming and ectoparasite control were minimal, at 6.3% and 1.0% respectively. Most farmers (87%) relied on ethnoveterinary concoctions to treat their morbid chickens compared to the few (13%) who used conventional medication (*p* < 0.001). Predominant among the ethno-materials were plant leaves (68%) while stems, seeds, fruits, barks, and wood ash were also sometimes used (Supplementary Figure S9B).

**FIGURE 3:**
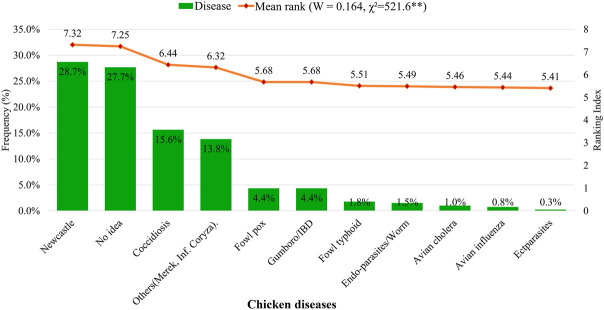
Disease prevalence in indigenous chickens of Uganda as described by households. W = Kendall’s coefficient of concordance. x^2^ = chi-squared.^**^ *p* < 0.001.

The farmers showed significant (*p <* 0.001) agreement in their ranking of the diseases that challenged their chicken flocks, based on experience (not pathological diagnosis). However, a significant proportion (27.7%) of the farmers had no idea about the diseases that challenged their production. Newcastle disease was ranked first (28.7%) as the most prevalent disease, followed by coccidiosis (15.6%). Other diseases of importance were Marek’s disease and infectious coryza (13.8%), Gumboro/infectious bursal disease (IBD) (4.4%), and a host of other minor health challenges including fowl typhoid, helminths, avian cholera/influenza, and some ectoparasites (Figure 3).

Most (38.4%) of the farmers administered healthcare services to their flock themselves or through a neighbour without any professional advice, while 22.0% sought professional advice (Supplementary Figure S10). Farmers reaching out to animal health service providers and governmental veterinarians constituted 17.3% and 17.0% respectively, as 4.3% accessed treatment from veterinary supply shops in their neighbourhoods. Community vaccinations were accessed by only a few (0.9%) indigenous chicken farmers.

#### Chicken identification and pedigree information

Regarding chicken identification and pedigree information, 95% of indigenous-chicken-keeping farmers hardly kept any form of written records on their production (Supplementary Figure S11). Nonetheless, 90% of them claimed they could recognise their chickens individually, though a planned outline of naming or identification of individual members of their chicken flock was non-existent in most (95%) of the households.

#### Productive performance and sales from indigenous chicken flocks in Uganda

Table 5 shows that most households (87.8%) generally had an average of 3.83 ± 0.29 laying hens in their flock; however, this number varied significantly across the regional households (*p <* 0.05). The Northern region had a higher (*p <* 0.01) average number of laying hens compared to the Western region but not the Central and Eastern regions. The average age at first lay was 5.20 ± 0.03 months (21 weeks). The average of 13.41 ± 0.20 eggs per hen per clutch was similar (*p >* 0.05) across regions. Consequently, an average of 40.22 ± 0.60 eggs were produced per year.

**TABLE 5:** Egg production and sale of indigenous chickens in Uganda.

Production variable	Regional households [n (%)]				Overall n = 253	χ^2^	*p*-value
	Northern n = 71	Central n = 42	Western n = 59	Eastern n = 80			
***Laying hen in the flock?***						19.528	<0.001
No	3 (4.1)	6 (12.2)	20 (25.3)	6 (7.0)	35 (12.2)		
Yes	71 (95.9)	43 (87.8)	59 (74.7)	80 (93.0)	253 (87.8)		
Laying hens (Mean ± SEM)	5.23 ± 0.52^a^	3.56 ± 0.67^a^ ^,^ ^b^	2.56 ± 0.58^b^	3.96 ± 0.49^a^ ^,^ ^b^	3.83 ± 0.29		0.008
***Egg numbers per laying hen***							
Age at laying (month)	5.21 ± 0.05^a^	5.02 ± 0.06^a^	5.07 ± 0.05^a^	5.50 ± 0.04^b^	5.20 ± 0.03		<0.001
Average eggs per clutch	13.06 ± 0.37	12.95 ± 0.47	13.98 ± 0.40	13.64 ± 0.35	13.41 ± 0.20		0.234
Average, 3 clutches (a year)	39.17 ± 1.10	38.86 ± 1.42	41.95 ± 1.21	40.91 ± 1.04	40.22 ± 0.60		0.234
***Eggs sold per laying cycle***						36.122	0.002
0	63 (88.7)	34 (79.1)	41 (69.5)	65 (81.3)	203 (80.2)		
1–10	-	1 (2.3)	-	8 (10.0)	9 (3.6)		
11–20	6 (8.5)	3 (7.0)	6 (10.2)	5 (6.3)	20 (7.9)		
21–30	-	3 (7.0)	7 (11.9)	2 (2.5)	12 (4.7)		
31–60	2 (2.8)	1 (2.3)	4 (6.8)	-	7 (2.8)		
61–90	-	1 (2.3)	1 (1.7)	-	2 (0.8)		
***Egg sales points***						28.924	0.004
Not sold, consumed/incubated	64 (90.1)	30 (69.8)	40 (67.8)	67 (83.8)	201 (79.4)		
Local market	6 (8.5)	4 (9.3)	8 (13.6)	7 (8.8)	25 (9.9)		
Within neighbourhood	1 (1.4)	3 (7.0)	6 (10.6)	1 (1.3)	11 (4.3)		
Retail shops	-	2 (4.7)	3 (5.1)	5 (6.3)	10 (4.0)		
Small-scale hatcheries	-	4 (9.3)	2 (3.4)	-	6 (2.4)		

^a^ Means within rows with different superscripts differ significantly (*p* < 0.05).

^b^ Means with no superscripts within rows do not differ significantly (*p* > 0.05). χ^2^ = chi-square test (-) = not reported, SEM, standard error of the mean.

A high proportion (80.2%) of farmers across the regions (*p <* 0.01) had not sold any eggs in the 12 months before the study. About 11.5% of them sold 20 eggs at most while a small proportion (8.3%) sold at least an egg crate (30 eggs) from their flock. Most (79.4%) of the eggs produced were used as food in the homestead or were incubated to hatch chicks, leaving a few for sale in local markets (9.9%) within the neighbourhood (4.3%), at retail shops (4.0%), and to small-scale hatcheries, mainly in the Central and Western regions (2.4%).

**FIGURE 4:**
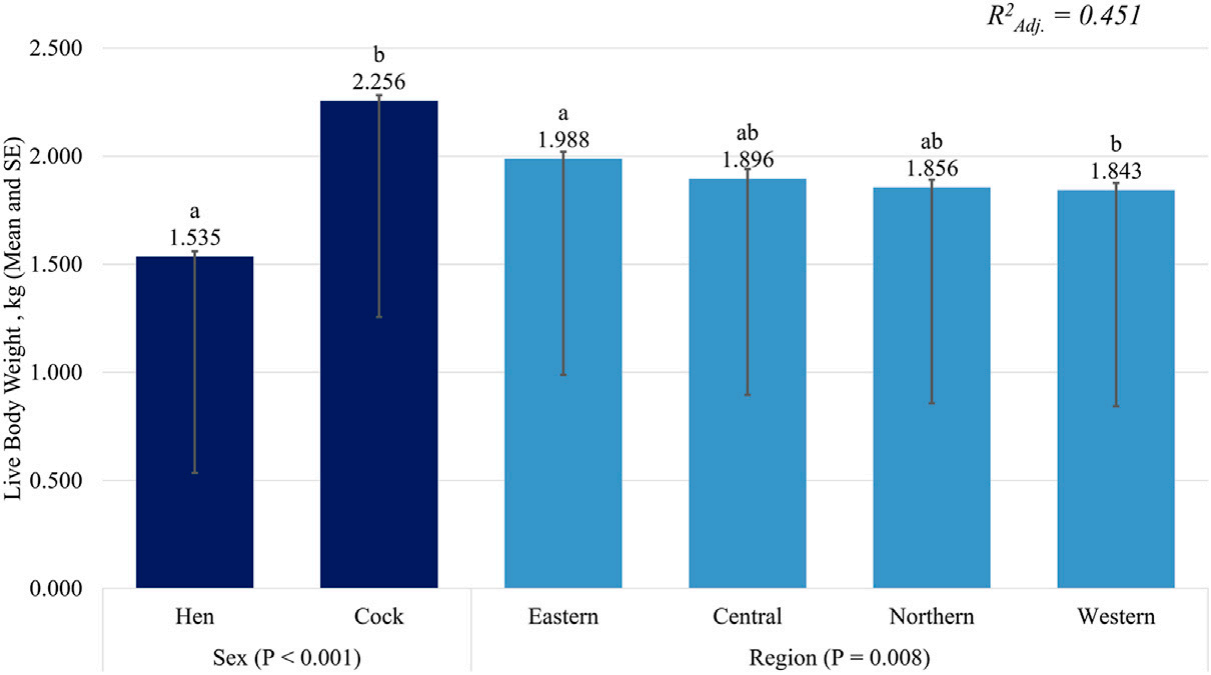
Mature body weight (mean and SE) of indigenous chickens in Uganda. ^a,b^Significant differences in within-factor mean bars (*p* < 0.05). x^2^ = chi-squared.

The mature chicken body weights (Figure 4) for market differed between sexes, with the cocks heavier (*p <* 0.001) than the hens. Regional differences (*p <* 0.05) were also observed, with chickens in the Western region lighter (*p <* 0.05) than those in the Eastern region but as heavy (*p >* 0.05) as those from the other regions.

### Phenotypic characteristics of indigenous chickens in Uganda

The total of 586 indigenous breeding chickens sampled for the phenotypic characterisation study were of similar (*p >* 0.05) sex categories, with overall comparable (*p >* 0.05) regional proportions (Supplementary Table S1 and Figure 1). Most of the breeding chickens in the household flocks from which samples were taken were in generally good body condition with moderately developed concave breast muscle and less prominent keel (*p >* 0.05) across households.

#### Feather morphology, distribution, and plumage patterns

**FIGURE 5:**
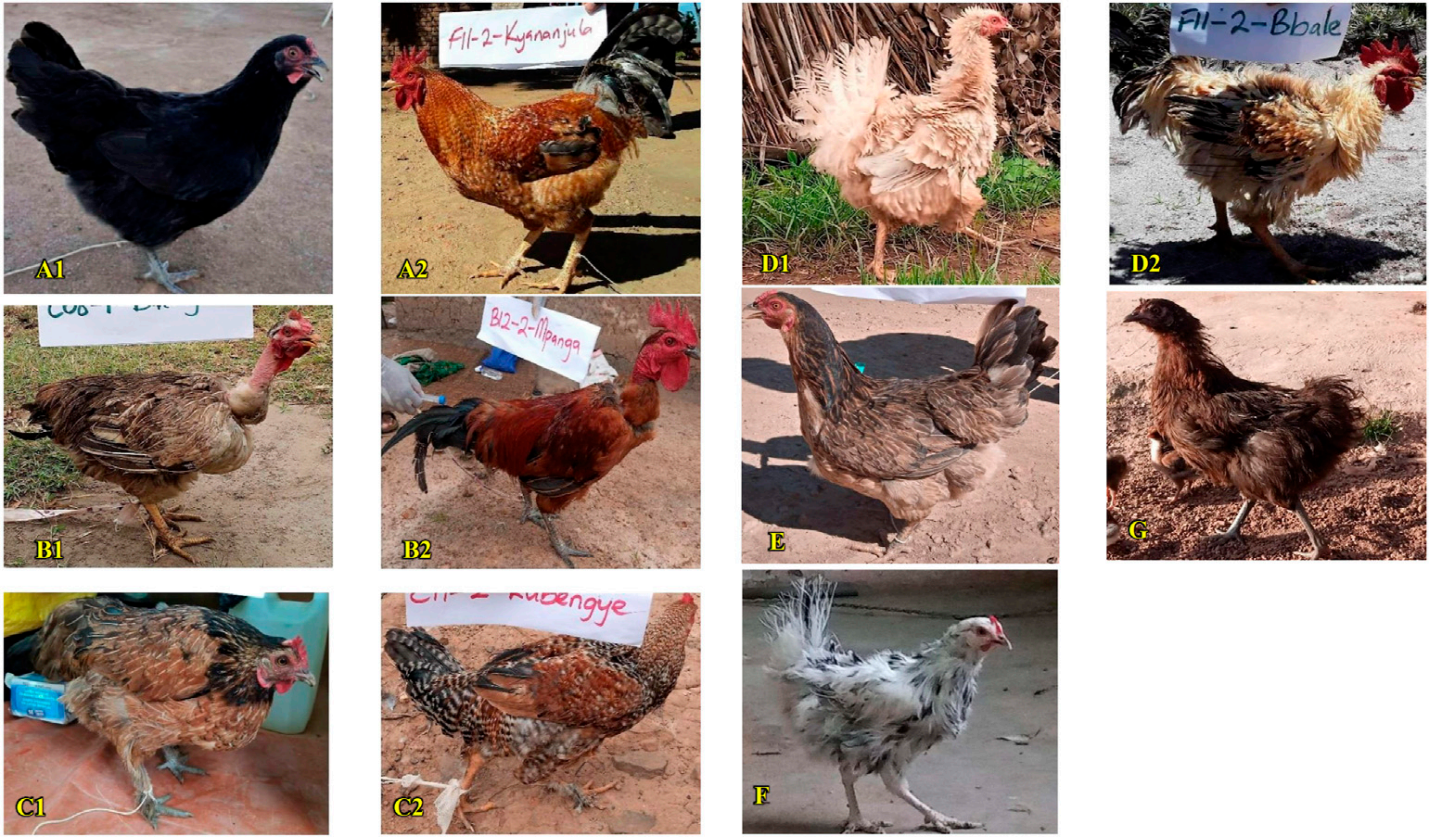
Feather distribution and structure in the indigenous chicken population in Uganda. **(Al, A2)** Normal feather distribution. **(Bl, B2)** Naked-neck. **(Cl, C2)** Ptilopody (feathered shanks/tarsus). **(Dl, D2)** Frizzle feather structure. **(E)** Smooth/neat plane feather. **(F)** Silky and **(G)** superficially silky feather structures.

The feather structure and distribution of the chickens varied significantly (*p <* 0.05) across the study locations. Most of the chickens across the regions (97.7% of hens and 95.5% of cocks) presented smooth/neat plane feather structures irrespective of sex (*p >* 0.05). However, a few isolated birds with frizzle, silky, and superficially silky feathers were also observed (Supplementary Table S4 and Figure 5). Normal feather structure was generally prevalent across the regions (90.3% of hens and 89.2% of cocks), with the sparse occurrence of naked neck trait and ptilopody (feathered shanks) in both sexes (*p >* 0.05) (Supplementary Table S4 and Figure 5).

The chickens displayed diverse plumage patterns (Supplementary Table S4 and Supplementary Figure S12) in both sexes across the regions (*p <* 0.001), most commonly the partridge (37.8%) and birchen (19.8%) patterns in cocks overall. In contrast, the hens generally presented mixed plumage patterns (25.2%), which were a blend of the eight- plumage patterns observed. The hens also presented high proportions of uniform (23.2%) and pencilled (21.5%) plumage patterns. The cocks showed the highest proportions of birchen (39.7%) plumage pattern in the Northern region but the lowest proportion (10.7%) in the Western region. Notably, the birchen pattern only existed (8.0%) in hens (*p <* 0.001) in the Northern region. Partridge was the predominant pattern in cocks in the Central, Western and Eastern regions, observed in 44.4%, 44.0%, and 34.9% of the birds, respectively. In hens, pencilled plumage pattern was dominant in the Central and Western regions, while uniform plumage occurred more in the Northern region. The Eastern region instead had a higher proportion of mixed plumage patterns in this study. Mottled and spotted patterns were the least frequent plumage across the regions.

#### Body colours of indigenous chickens in Uganda

**FIGURE 6:**
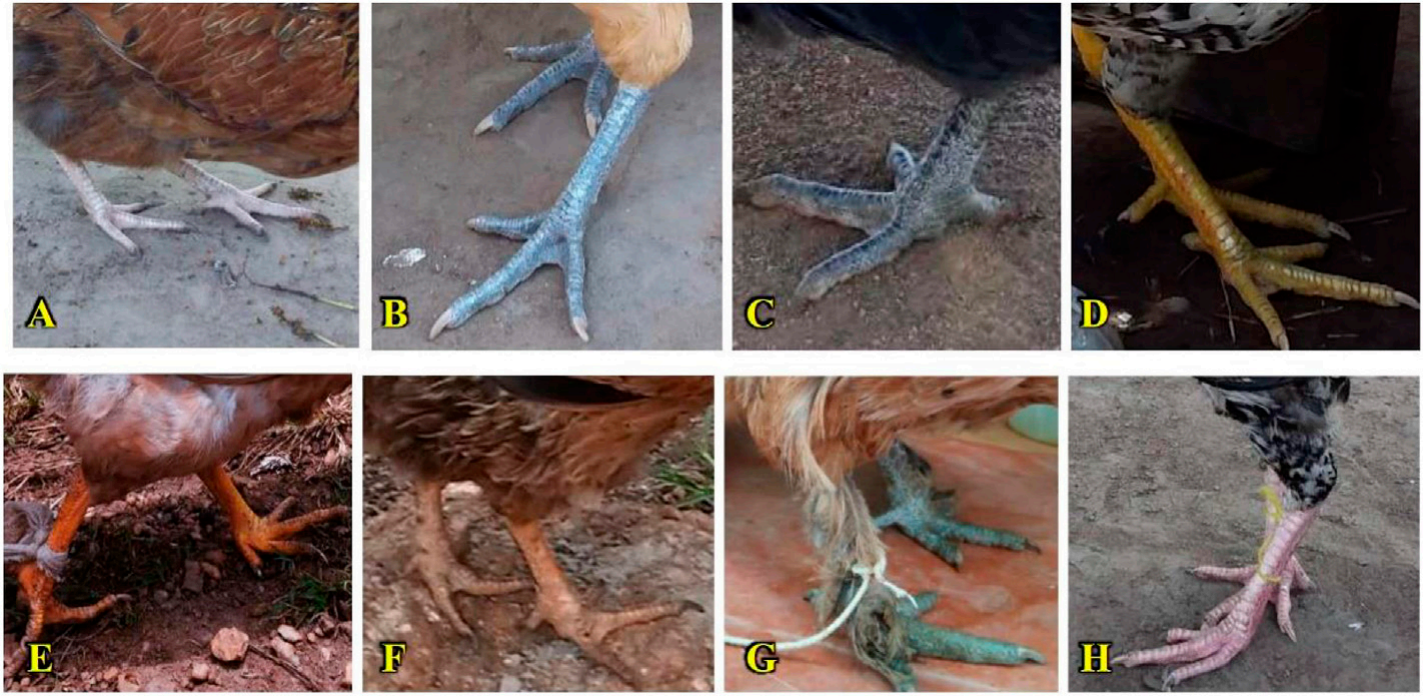
Shank (tarsus) colours. **(A)** White. **(B)** Grey/blue-grey. **(C)** Black/dark grey. **(D)** Yellow. **(E)** Orange. **(F)** Brown. **(G)** Green. **(H)** Pink.

The skin colours of the chickens differed (*p <* 0.001) with location irrespective of sex (*p >* 0.05) (Supplementary Table S5). The proportion of purplish-brown/dark-skinned chickens was slightly higher (49.0% of hens and 43.8% of cocks) than those with white (38.3% of hens and 42.4% of cocks) and yellow (12.8% of hens and 13.9% of cocks) skins. The dominant tarsus (shank) colour varied widely across regions and between sexes (*p <* 0.01). However, yellow tarsi were more frequent, followed by grey/blue-grey, black/dark-grey, white, and other less occurring tarsi colour variants including pink, green, brown, and orange, in varying proportions (Figure 6 and Supplementary Table S5). The eye (iris) colours also differed across regions (*p <* 0.01) irrespective of sex (*p >* 0.05). The most common eye colours were orange, brown, and yellow in both sexes, with comparable proportions (*p >* 0.05). Very low proportions of chickens across locations exhibited dark-green, pink, red, and cyan/blue eye colours. Black eye colour was rare, with only one occurrence in a hen in Western Uganda (Supplementary Table S5).

The location and sex of the chickens influenced the incidence of the earlobe and beak colours in the chicken populations (*p <* 0.01) (Supplementary Figure S13 and Supplementary Table S5). Large variations were observed in the earlobe colour diversity of the chickens, with instances of red and pink earlobes blemished with white, cyan, or yellow. This made it challenging to characterise them into distinct phenotypes. Consequently, the dominant earlobe colours were grouped into broader phenotypes from a pool of related ones. The hens presented pink and dark-red earlobes particularly in similar proportions (21%), followed by light red (16.8%), yellow (15.1%), cyan-blue (10.7%), and white (9.7%) earlobes, compared to the cocks, which mostly showed light (70.1%) or dark (13.2%) red earlobes, with other colours not common. Regionally, yellow (20.3% of hens and 20.3% of cocks) and dark red (18.9% of hens and 28.8% of cocks) earlobes were more pronounced in hens from Northern and Central respectively. Hens in Western Uganda showed quite a wide variability in earlobe colours, including white (29.1%), cyan/blue (19.0%), yellow (17.7%), and light red (16.5%) variants, as well as other low-occurring earlobe colours. Similarly, 28.7%, 27.6%, and 21.8% of hens from Eastern Uganda had dark red, light red, and pink earlobe variants, respectively. Green and grey earlobes variants were subtle across regions and in both sexes. The combs and wattles of indigenous chickens in Uganda were generally red (data not shown), except for a few variants with white and black wattles. The beak colours (Supplementary Figure S14 and Supplementary Table S5) in most cocks were grey, brown, and yellow (31.6%, 18.8%, and 18.4%, respectively), whereas the hens presented wide variations in beak colour, with 21.5% yellow, 19.5% brown to grey, and 18.1% brown-purple.

#### Head features of indigenous chickens in Uganda

All the descriptive head features (Table 6 and Supplementary Figure S15) were significantly (*p <* 0.05) associated with the sex of the chickens. However, the locations were not significantly associated with the presence of wattles and crests (*p >* 0.05). Hens presented mostly small earlobes compared to cocks, which had large, prominent earlobes. Oval-shaped, large earlobes were more predominant amongst cocks than in hens, which mostly mostly had the round-shaped variant across Uganda. The single-comb type was almost universal (>93%) in both sexes, with rose combs occurring in low proportions scattered across the regions. The double-comb variant occurred exclusively in the Northern region, while the pea comb occurred mainly in cocks from the Central and Western regions. Only a few birds had rudimentary or lacked combs. The presence of wattles was universal, especially in cocks. Crests, on the other hand, mainly existed at low frequencies with just a few occurring across locations in both hens (12.1%) and cocks (4.9%). Bearded chickens (Supplementary Figures S16E1, E2) also occurred (data not shown) but were rare. Hooked beaks generally occurred in both hens and cocks. However, straight beaks were observed more often in the chicken population from the Central and Western regions.

**TABLE 6:** Variations in the descriptive head features of indigenous chickens in Uganda.

Qualitative traits [n (%)]	Northern		Central		Western		Eastern		Uganda		χ^2^	
	*Hen* n = 75	*Cock* n = 73	*Hen* n = 58	*Cock* n = 54	Hen n = 78	*Cock* n = 75	*Hen* n = 87	*Cock* n = 86	*Hen* n = *298*	*Cock* n = *288*	*Sex*	*Region*
*Earlobe size*											304.3^***^	16.4^**^
Small^a^	61 (81.3)	13 (17.8)	57 (98.3)	18 (33.3)	77 (98.7)	27 (36.0)	83 (95.4)	7 (8.1)	279 (93.3)	65 (22.6)		
Large^b^	14 (18.7)	60 (82.2)	1 (1.7)	36 (66.7)	1 (1.3)	48 (64.0)	4 (4^b^6)	79 (91.9)	20 (6.7)	223 (77.4)		
*Earlobe shape*											161.8^***^	60.2^***^
Oval	33 (44.6)	66 (90.4)	4 (6.9)	32 (59.3)	5 (6.3)	43 (57.3)	27 (31.0)	78 (90.7)	69 (23.2)	219 (76.0)		
Round	41 (55.4)	7 (9.6)	54 (93.1)	22 (40.7)	74 (93.7)	32 (42.7)	60 (69.0)	8 (9.3)	229 (76.8)	69 (24.0)		
***Comb type***											13.5^**^	22.5^*^
Absent	2 (2.7)	2 (2.7)	NR	NR	4 (5.1)	NR	2 (2.3)	2 (2.3)	8 (2.7)	4 (1.4)		
Single	70 (93.3)	69 (94.5)	58 (100.0)	52 (96.3)	73 (93.6)	68 (90.7)	82 (94.3)	80 (93.0)	283 (95.0)	269 (93.4)		
Double	3 (4.0)	NR	NR	NR	NR	NR	NR	NR	3 (1.0)	NR		
Rose	NR	2 (2.7)	NR	1 (1.9)	1 (1.3)	6 (8.0)	3 (3.4)	4 (4.7)	4 (1.3)	13 (4.5)		
Pea	NR	NR	NR	1 (1.9)	NR	1 (1.3)	NR	NR	NR	2 (0.7)		
*Presence of wattles*											8.2^**^	6.0^ns^
Absent	2 (2.7)	NR	3 (5.2)	NR	1 (1.3)	NR	NR	NR	6 (2.0)	NR		
Present	73 (97.3)	73 (100.0)	55 (94.8)	54 (100.0)	77 (98.7)	75 (100.0)	87 (100.0)	86 (100.0)	292 (98.0)	288 (100)		
***Presence of crest***											9.8^**^	2.1^ns^
Absent	67 (89.3)	70 (95.9)	48 (82.8)	52 (96.3)	70 (89.7)	73 (97.3)	77 (88.5)	79 (91.9)	262 (87.9)	274 (95.1)		
Present	8 (10.7)	3 (4.1)	10 (17.2)	2 (3.7)	8 (10.3)	2 (2.7)	10 (11.5)	7 (8.1)	36 (12.1)	14 (4.9)		
***Beak shape***											4.6^*^	327.7^***^
Hooked	56 (74.7)	59 (80.8)	9 (15.5)	16 (29.6)	4 (5.1)	16 (21.3)	87 (100)	85 (98.8)	156 (52.3)	176 (61.1)		
Straight	19 (25.3)	14 (19.2)	49 (84.5)	38 (70.4)	74 (94.9)	59 (78.7)	NR	1 (1.2)	142 (47.7)	112 (38.9)		

^*^ *p* < 0.05; ^**^ *p* < 0.01; ^***^ *p* < 0.001; ns = non-significant; χ^2^ = chi-square test of fixed variables; n = chickens sampled; NR, not reported.

^a^ Rudimentary-form earlobes for each chicken category.

^b^ Prominent earlobes.

#### Skeletal variance among indigenous chickens of Uganda

**FIGURE 7:**
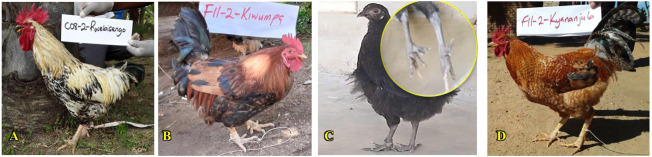
Skeletal variance. **(A)** Rumpless. **(B)** Dwarf. **(C)** Polydactyl. **(D)** Normal skeletal structure.

The skeletal variance among the chicken population (Supplementary Table S6) was significantly associated with region (*p <* 0.05) but not the sex of the birds. Generally, the chicken populations were skeletally normal across Uganda. Traces of polydactylous chickens (more than the normal four-digit toes) as well as dwarf and rumpless chickens were also observed in mainly the Northern, Central, and Western regions (Figure 7). The body frame of most of the chickens was medium (79.2% of hens and 60.1% of cocks), with significant (*p* < 0.05) differences across the regions in both sexes. Hens showed a higher proportion of medium body frames across the regions except in Western Uganda, where most hens had rudimentary frames. Cocks were more likely to have long body frames across the regions, while hens were more likely to have rudimentary frames (Supplementary Figure S17 and Supplementary Table S6). Higher proportions of hens with medium body conformation were observed in the Northern and Eastern regions than those in the Central and Western regions, which showed more blocky-compact body conformations. Hence, medium and blocky-compact conformation was more likely in hens, whereas tall-angular body shape was more likely in cocks across the regions (Supplementary Figure S18). Rudimentary to small spurs were mostly observed in hens across regions whilst cocks largely had well-projected spurs across the regions (Supplementary Figure S19). Similarly, short to medium tail lengths were observed in hens whereas the cocks developed medium to long tails (Supplementary Figure S17).

#### Allele and phenotypic frequencies of indigenous chicken strains in Uganda

The distributions and allele frequencies of the indigenous chicken strains were 0.13, 0.20, 0.29, 0.14, and 0.17 for frizzle, ptilopody, tufted crest, polydactyl, and rose comb chickens (Table 7). Generally, the occurrence of chickens exhibiting the genotype that characterised major traits were relatively low. However, the tufted crest, which was the most common (50; 8.53%) genotype, had an allele frequency of 0.29, which was statistically (*p >* 0.05) similar to the expected Mendelian proportion of 0.25. Likewise, the allele frequencies for ptilopody and rose comb had similar (*p >* 0.05) estimates to the Mendelian proportion of 0.25. However, the frizzling and polydactyl allele frequencies, 0.13 and 0.14 respectively; were significantly lower (*p <* 0.05) than the expected Mendelian frequency of 0.25.

**TABLE 7:** Occurrence and allele frequencies of rare major genotypes in the indigenous chicken population.

Strains [allele]	N	Observed frequency %	χ^2^	Expected frequency %	Calculated allele frequency	Expected allele frequency	χ^2^
Frizzle [F]	10	1.71^a^	*169.6* ^***^	25	0.13^a^	0.25	*7.680* ^**^
Other [f+]	576	98.29^b^		75	0.87^b^	0.75	
Ptilopody [Pti]	24	4.10^a^	*136.6* ^***^	25	0.20^a^	0.25	*1.333* ^*ns*^
Other [pti+]	562	95.90^b^		75	0.80^a^	0.75	
Crest [Cr]	50	8.53^a^	*84.8* ^***^	25	0.29^a^	0.25	*0.853* ^*ns*^
Other [cr+]	536	91.47^b^		75	0.71^a^	0.75	
Polydactyl^c^ [P]	11	1.88^a^	*167.1* ^***^	25	0.14^a^	0.25	*6.453* ^*^
Other [p+]	575	98.12^b^		75	0.86^b^	0.75	
Rose comb [R]	17	2.90^a^	*152.6* ^***^	25	0.17^a^	0.25	*3.413* ^*ns*^
Other [r+]	569	97.10^b^		75	0.83^a^	0.75	

^a^ Values followed by different superscripts in the same column differ significantly (*p <* 0.05).

^b^ Other = Individuals not displaying the trait.

^c^ Feathered shank trait; n = counts. χ^2^ = chi-squared estimate. ***p < 0.001, **p < 0.01, *p < 0.05, ^ns^ not significant.

#### Morphometric traits of the major strains (genotypes) among indigenous chickens in Ugandan

The body weight and linear measurements of the circumferences of the thorax and shanks; lengths of the thigh, shank, comb, and wattle; and the comb height were not significantly (*p >* 0.05) associated with genotypes (strains having major alleles) in either sex (Table 8). However, naked-necked cocks had significantly (*p <* 0.05) taller combs compared to rose comb cocks (5.05 ± 0.37 cm vs 2.46 ± 0.51 cm). Wattle length in both naked-neck hens and cocks was longest compared to the normal, frizzle, polydactyl, rose comb, and tufted crest strains. The shank (tarsus) length in both the hen and cock of rose comb chickens was larger than those in the other chicken strains. Numerically, shank length and comb height and length in hens were higher in the polydactylous and frizzle chicken strains respectively. Whereas in cocks, marginally higher shank length and comb height and length, were observed in the naked-neck and polydactylous strains compared to the other strains.

**TABLE 8:** Least-square means (LSM ±S.E) of morphometric measurements among the strains of the indigenous chicken population in Uganda.

Morphometric variables	Strains (major phenotypes)						
	Normal feather	Naked neck	Frizzle	Polydactyl	Ptilopody	Rose comb	Tufted crest
*Hens*							
Live weight (LW), kg	1.54 ± 0.02	1.55 ± 0.08	1.66 ± 0.16	1.27 ± 0.23	1.78 ± 0.10	1.58 ± 0.16	1.47 ± 0.06
Thorax circumference (TC), cm	33.61 ± 0.32	35.13 ± 1.14	35.13 ± 2.41	32.5 ± 3.41	36.80 ± 1.53	32.98 ± 2.41	34.11 ± 0.85
Thigh length (TL), cm	13.16 ± 0.09	13.59 ± 0.34	13.88 ± 0.71	12.50 ± 1.01	13.60 ± 0.45	13.75 ± 0.71	13.03 ± 0.25
Shank circumference (SC), cm	3.93 ± 0.04	3.90 ± 0.13	3.93 ± 0.27	3.75 ± 0.38	3.89 ± 0.17	4.15 ± 0.27	3.85 ± 0.10
Shank length (SL), cm	7.82 ± 0.07	8.03 ± 0.24	7.75 ± 0.52	8.50 ± 0.73	7.81 ± 0.33	8.15 ± 0.52	7.29 ± 0.18
Comb height (CH), cm	1.29 ± 0.05	1.28 ± 0.17	1.60 ± 0.35	0.40 ± 0.50	1.59 ± 0.22	0.75 ± 0.35	1.25 ± 0.12
Comb length (CL), cm	3.05 ± 0.06	3.17 ± 0.22	3.53 ± 0.46	2.50 ± 0.65	3.53 ± 0.29	2.43 ± 0.46	2.52 ± 0.16
Wattle length (WL), cm	1.21 ± 0.06	1.59 ± 0.19	1.15 ± 0.41	0.50 ± 0.58	1.17 ± 0.26	1.18 ± 0.41	1.17 ± 0.15
*Cocks*							
Live weight (LW), kg	2.22 ± 0.03	2.58 ± 0.13	2.33 ± 0.21	2.18 ± 0.21	2.35 ± 0.16	2.27 ± 0.18	2.32 ± 0.16
Thorax circumference (TC), cm	38.1 ± 0.41	39.56 ± 1.59	40.17 ± 2.52	37.58 ± 2.52	37.55 ± 1.86	37.25 ± 2.18	40.91 ± 1.86
Thigh length (TL), cm	16.24 ± 0.11	16.53 ± 0.41	15.00 ± 0.65	15.92 ± 0.65	16.77 ± 0.48	15.38 ± 0.56	16.59 ± 0.48
Shank circumference (SC), cm	4.76 ± 0.04	4.98 ± 0.17	4.82 ± 0.26	4.85 ± 0.26	4.85 ± 0.19	5.04 ± 0.23	4.68 ± 0.19
Shank length (SL), cm	9.77 ± 0.08	10.63 ± 0.31	9.08 ± 0.49	10.42 ± 0.49	9.67 ± 0.36	10.00 ± 0.42	9.86 ± 0.36
Comb height (CH), cm	3.84^a^ ^,^ ^b^ ± 0.10	5.05^a^ ± 0.37	3.48^a^ ^,^ ^b^ ± 0.59	4.25^a^ ^,^ ^b^ ± 0.59	3.85^a^ ^,^ ^b^ ± 0.44	2.46^b^ ± 0.51	4.21^a^ ^,^ ^b^ ± 0.44
Comb length (CL), cm	7.40 ± 0.14	8.45 ± 0.55	6.70 ± 0.87	8.67 ± 0.87	7.56 ± 0.64	5.83 ± 0.75	7.52 ± 0.64
Wattle length (WL), cm	3.74 ± 0.08	4.61 ± 0.33	3.77 ± 0.51	3.77 ± 0.51	3.59 ± 0.38	3.61 ± 0.45	3.83 ± 0.38

^a^ Means with different superscripts within rows differ significantly (*p <* 0.05).

^b^ Means with no superscripts within rows do not differ significantly (*p* > 0.05).

#### Correlations between morphometric traits of indigenous chicken strains in Uganda

The Pearson correlations between the morphometric traits of indigenous chicken strains in Uganda for both cocks and hens are shown in Supplementary Table S7. The correlation coefficients generally differed from zero (*p* < 0.05), except for comb height (CH) and shank circumference (SC), CH and shank length (SL), comb length (CL) and SL, and WL and TL in hens. The correlation coefficients for all pairs of morphometrics measured were much stronger in cocks than in hens, with only LW and TC and CH and TC being comparable.

## Discussion

This study sought to perform extensive and comprehensive across-country investigations to characterise the indigenous chicken production dynamics and the phenotypic and morphologic differentiation of chickens across the Ugandan landscape. Such large-scale characterisation reports are scarce, thus making this study foremost within the country. These results indicated that the flock description, production dynamics, performance, and phenotypic characteristics of indigenous chickens were similar to those reported elsewhere within the continent (Njenga, 2005; Adomako, 2009; Dana, 2011; Dessie et al., 2011; Marwa et al., 2016; Mahoro et al., 2017; Hirwa et al., 2019; Otecko et al., 2019).

### Demographic profile of indigenous chicken-rearing households in Uganda

The description of the demographic profile of the indigenous chicken-keeping households is key to determining the socio-economic features of the household, which is necessary for the implementation of sustainable improvement programmes. Across the studied households in Uganda, higher proportions of male chicken farmers were observed, except for the Central region, which showed higher numbers of female chicken keepers. For such a society more likely to be headed by a man, having women contend favourably in chicken rearing supports the claim that household chicken keeping is sometimes the only livestock species under the control of women (Kyarisiima et al., 2004; Dana, 2011; Mahoro et al., 2017). Gender roles imposed by the traditional setting in Northern Uganda and the capacity to exert control over income from household chicken production, especially by women who headed households, influence women’s participation in the chicken value chain (Akite et al., 2018). The responses obtained in this study were mainly from adults (≥22 years old) who were household heads, spouses, or other household members. Therefore, they were deemed reliable to infer the production characteristics of indigenous chickens in Uganda. Besides, the age range between 31 and 60 years of most of the farmers was consistent with earlier findings in Uganda (Kugonza et al., 2008; Kayitesi, 2015) and neighbouring Rwandan local chicken keepers (Mahoro et al., 2017; Hirwa et al., 2019). The similar mean distances from a farmer’s homestead to an all-weather road (33.3 km) and the market (5.6 km) across the regions indicated the use of representative samples, including those from the hinterlands. This further ensured that the sampling of related chickens was avoided. The medium average agrarian household size of 7.6 observed across the regional households was above the reported average of five in rural residents (UBOS, 2021). The households derived their livelihoods mostly from the sale of crop products and livestock and livestock products. A few households traded livestock and livestock products and participated in off-farm activities. About 3.3 million households in Uganda live in a subsistence economy, which is defined to include 62% subsistence-farming households engaged in agriculture mainly for household consumption and sale/barter according to the UBOS (2021). This emphasises the importance of household livestock and the role of indigenous chicken farming under the low input management in peoples’ livelihoods in Uganda.

### Ownership and flock description of household chickens in Uganda

The flock size averaging 19.80 ± 1.21 chickens across the regions in this study is consistent with earlier findings in Eastern Uganda (Kugonza et al., 2008), along with the increasing trend of small flock holdings. The low numbers of cocks in the flock but higher numbers for hens, growers, and chicks are also consistent with earlier reports (Kyarisiima et al., 2004; Ssewannyana et al., 2008; Nakkazi et al., 2014). The flock sizes held by women were small (1–10 chickens) compared to those of men. The opposite was true, as 53.6% of men kept 16 to over 20 chickens. The African Livestock Futures (Herrero et al., 2014) describes women’s small chicken flock size as a special policy problem emanating from their typically lower mobility due to domestic work. Thus, the women can only manage small flocks along with their chores. As such, women are more likely to lose on policies seeking to cushion large-scale enterprises like public subsidies, which could boost their markets and their corresponding income. Those that could benefit them as smallholders like animal health services, when underserved also result in a disproportionate loss. Therefore, women are usually not able to grow their flock size. Mature cocks, followed by mature hens or the other chicken categories were usually the first to exit the flock. Most farmers usually kept cocks for up to or over 1 year before they were sold. The exchange of chickens to acquire other livestock species was consistent with reports of the acquisition of cattle and goats through barter trading or the use of cash proceeds from chicken production (Iisa, 2003; Kugonza et al., 2008).

Indigenous chicken farmers in Uganda shared similar production objectives. The generation of income from sales and the consumption of chicken meat and eggs were considered the most important, consistent with previous reports in Uganda (Kugonza et al., 2008; Natukunda et al., 2011). The minor production objectives pertained to the benefits derived from by-products including chicken droppings as organic fertilizer, socio-economic/prestige and cultural heritage, gifts to visitors, and/or barter trading to obtain other livestock species and leisure purposes.

### Indigenous chicken production, management system, and performance in Uganda

The mixed crop-livestock systems fall under one of the broad classifications of livestock production systems in Africa, mainly in places with high rainfall and crop production potential (AU-IBAR, 2019). This could explain the predominance of mixed crop-livestock farming across Uganda, as reported previously (Kugonza, 2008). The scavenging feeding management across indigenous chicken-keeping households in Uganda, with only a few households providing supplementation, is consistent with earlier reports in Uganda (Kugonza et al., 2008; Natukunda et al., 2011; MAAIF, 2019) and findings elsewhere (Moreda et al., 2013; Mahoro et al., 2017; Hirwa et al., 2019). Scavenging chicken production is popular among resource-challenged rural communities in most parts of the developing world to derive their livelihoods (Melesse, 2014).

The feed resources available to the indigenous chickens were mainly pasturage, from which they foraged on grasses, seeds, lush leaves, and other plant resources near and around the homestead. Other scavenged feed resources were kitchen refuse or agro-by-products, with little grain concentrates. Ssewannyana et al. (2008) found that indigenous chickens are valued for their ability to scavenge in Uganda. Uncontrolled mating characterises a scavenging feeding system, which limits the production of indigenous chickens due to inbreeding. Intensively fed indigenous chickens performed better than those kept under semi-scavenging conditions (Magala et al., 2012; Nakkazi et al., 2015). Interestingly, water was not consciously provided to the chickens. The chickens must, therefore, search for water as they scavenge near and around their homestead, as most households had good clear water resources within the scavenging radius of their flock.

Housing for both adult and young chickens varied from predominantly bespoke enclosures constructed on the side of the main house, sheds, and human dwelling units to less common shelters like above-ground perches/kitchens and stalls (Supplementary Figure S7–S9). A similar situation occurs in Rwanda, where most farmers house their flocks in enclosures (Hirwa et al., 2019). The occasional observation of chicken flocks housed in human-dwelling units and stalls in some households is consistent with reports of chickens not being provided with specific housing in Uganda (Kyarisiima et al., 2004; Natukunda et al., 2011). Like in many African countries, housing is either specifically fabricated for indigenous chickens or the chickens seek shelter in the natural surroundings under the prevailing scavenging management (FAO, 2008).

The results of the present study generally revealed a mix of different episodes of disease challenges in indigenous chicken flocks across the regions. Most were once-a-while disease challenges occurring at random and yearly/seasonal episodes. Flocks in which disease episodes were rare, especially in Western Uganda, might be due to the adaptive survival of the chickens to agro-ecologic conditions, which impact disease incidence and severity. Host genetics also impact resistance in diverse ways (Khobondo et al., 2015; Richardson, 2016). Berghof et al. (2019) posited differences in immunity and disease resistance because of growth differences in individuals. As such, the body weight deviations in the chickens from the Western region might have conferred resilience in the flock to some extent. Most indigenous farmers across households in Uganda treat their sick chickens, while only a quarter leave their sick chickens to recover on their own or die without any treatment during disease episodes. Meanwhile, a small proportion of farmers across Uganda seldom have disease situations in their flock, which they associated with their use of phyto-prophylactic remedies. Newcastle disease, as the major disease challenging indigenous chicken farming in Uganda in this study, corroborates earlier reports in Uganda (Kugonza et al., 2008; Natukunda et al., 2011), with significant minor diseases including coccidiosis, and a host of others. Contrary to the case in Uganda, coccidiosis is as most significant disease challenge in Rwandan indigenous chicken production, while Newcastle disease constituted a third major disease (Hirwa et al., 2019). Healthcare services for indigenous chickens are mainly curative treatments during the disease incidence, while vaccinations are also administered. A total of 13.2% of chicken keepers did not provide any health service to their flock. Deworming and ectoparasite control are less commonly practiced. Likewise, providers of healthcare services for indigenous chicken production are limited, as most farmers resort to self-services with or without professional advice. The use of local or herbal remedies as treatments against chicken diseases has been the main practice by most indigenous chicken farmers in Uganda (Kyarisiima et al., 2004; Natukunda et al., 2011).

Record-keeping, which is relevant for farm evaluation for key decision-making among most indigenous chicken farmers, was non-existent. Recognition of chickens is, therefore, mostly based on visual cues such as plumage colours or patterns and chicken types to differentiate chicken flocks.

Regarding the production performance of the hens, most farmers across Uganda had an average of 3.83 ± 0.29 laying hens, which was higher than the estimate in the Western region. The average age at first egg production of 5.20 ± 0.03 months ranged between 5.1 and 7.0 months, within the reported range of 20–21weeks for hens managed under an extensive system (FAO, 2003), as reported in Eastern Uganda (Kugonza et al., 2008). An average of 13.41 ± 0.20 eggs/hen/clutch was produced, resulting in 40.22 ± 0.60 eggs/hen/year. These findings matched those reported previously (Mogesse, 2007; Mahoro et al., 2017; Hirwa et al., 2019). However, the estimates were below the reported range of 50–60 eggs per annum of 10–12 eggs per clutch in the Domestic Animal Diversity information system (DAD-IS, 2022). The egg production potential of indigenous chickens, as reviewed by Padhi (2016) is low compared to dual-purpose breeds or commercial laying chickens, which produce about six times that of indigenous chickens (Farooq et al., 2002). Low egg production in indigenous chickens is associated with broodiness, which involves incubating their eggs to hatch embryos, as well as caring for their hatchlings. This period is accompanied by frequent nesting, reduced feed and water intake, increased body temperature, and, ultimately, cessation of laying during the broody periods. However, management interventions to preclude broodiness increase the laying ability of broody hens (Kugonza et al., 2008; Hossen, 2010; Jiang et al., 2010). Additionally, the average mature live body weights of chickens to be sold were 1.535 ± 0.025 kg and 2.256 ± 0.026 kg for hens and cocks, respectively, within the ranges of 1.03–2.05 kg and 1.25–2.86 kg reported for hens and cocks in Uganda (Kyarisiima et al., 2004).

### Phenotypic characteristics of the indigenous chickens in Uganda

The description of livestock breed characteristics is necessary to guide decision-making and is valuable for strategic development and breeding programmes (FAO, 2007). The general observation of the good body condition of most of the breeding chickens across households affirmed the ability of indigenous chickens to thrive well under the prevailing low-input conditions (Dessie et al., 2011).

#### Feather morphology of the indigenous chickens in Uganda

Feather morphology is often used to group chicken populations into sub-groups due to its importance in evolutionary biology and the socioeconomics of farmers. This trait influences preferences for a particular type of chicken, which determined its importance across the surveyed households. Most farmers in Uganda kept chickens with smooth-neat plane feather structures whilst frizzles, silkies, and chickens with superficial feathering were less common. Consequently, most of the indigenous chickens of both sexes had normal feather distributions. Special phenotypes (strains), including naked necks, feathered-shanks (ptilopody), and frizzle chickens, were present at low proportions similarly in both sexes, corroborating reports of the predominance of normal-feathered chickens in the chicken genetic resources in Rwanda (Hirwa et al., 2019), Ethiopia (Fistum, 2016), Ghana (Mensah, 2016; Brown et al., 2017), Nigeria (Yakubu, 2010), and Algeria (Dahloum et al., 2016). The low proportion of naked neck, feathered-shank (ptilopody), and frizzle traits, despite their favourable effects on production and tropical adaptation (Adomako, 2009; Mensah, 2016), is linked to socio-cultural/religious reasons (Njenga, 2005; Desta, 2020). Naked neck and frizzle-feathered phenotypes are negatively selected since they do not fetch a premium market price on grounds of undesirable aesthetic value (Yakubu, 2010; Desta et al., 2013). Moreover, anecdotal reports and personal interactions with the farmers in this study suggested that such chicken strains are ordinarily for home consumption and ritual sacrifices, as reported elsewhere (Njenga, 2005; Desta et al., 2013); hence, considered fetish. Moreover, the frizzle gene is reportedly detrimental in unfavourable environmental conditions in its homozygous state and causes internal organ abnormalities (Fathi et al., 2013), suggesting their very low frequencies. We observed a high variation in plumage patterns across the regions. Most of the hens in Eastern Uganda did not have a defined plumage pattern. However, the hens with patterns that could be described were of similar frequencies as the pencilled (in Western and Central regions), and uniform (in the Northern region) patterns of white, and black plumage colours. Cocks more often showed a partridge pattern, followed by the birchen pattern, with a host of other undefined patterns. Similar observations were made by Ssewannyana et al. (2008) who described the feathering in most indigenous chickens in Uganda as having no definite patterns. The low frequencies of some of the plumage patterns, and colours might be due to the preference of such chickens exclusively for sociocultural/religious ritual practices (Desta, 2020) and the lack of particular consumer demand for certain plumage patterns, consequently leading to a decline in population genetic structures. Whereas the largely non-descript hen plumage is perhaps due to farmers’ understanding of multiple plumage colours as camouflage against aerial predators (Besbes et al., 2007) and as part of the hen’s mothering ability (Kyarisiima et al., 2004).

#### Body colours of indigenous chickens in Uganda

The proportions of dark-skinned (purplish-brown/dark) and white-skinned chickens were similar, while that of yellow skin colouration was lowest among the observed skin phenotypes. This is contrary to earlier reports of subtle (1%) dark skin colour phenotype in Ugandan indigenous chickens (Ssewannyana et al., 2008) and the dominance (69%) of yellow-skinned chickens in the Rwandan indigenous breeds (Hirwa et al., 2019) and the Ethiopian chicken population (Desta et al., 2013; Negassa et al., 2014). Skin colour variations are influenced by a combination of genes and modifiers that influence melanin pigmentation and carotenoid deposition in the skin, in addition to environmental factors such as diet and physiological state (Jin et al., 2016). Dark skin colour correlates with melanin pigmentation, caused by the absence of sex-linked dermal melanin-inhibiting mutation (*Id*) and some plumage colour-influencing genes like the extension (*E*) locus alleles. Additionally, the fibromelanosis (*Fm*) locus gene together with dermal melanin *id* ^*+*^ causes melanised pigments in the skin and connective tissues (Jacob, 2022). White skin colour, however, is associated with the autosomal dominant white (*W*) locus allele and the non-extended black (*e*) allele or the combination of the *Fm* gene and *Id* mutation, which results in no visible skin colouration (Jin et al., 2016).

The white-skin gene reportedly originated from red jungle fowl (believed to be the ancient ancestor of domestic chickens), while the yellow-skin gene is from the grey jungle fowl (Eriksson et al., 2008), suggesting a dual origin of the trait. The recessive yellow skin (*w*) allele is related to carotenoid pigmentation (Jin et al., 2016).

Regarding shank/tarsi colours, the yellow dominant (primary) phenotypes were the most common in both sexes across the locations, indicating carotenoid deposition in the skin of the shanks (Gunnarsson, 2009). Grey/blue-grey shank was the second most frequent phenotype; however, hens were more likely to have grey-to-black shanks compared to cocks. The white shank phenotype ranked third in occurrence in both sexes. These findings are consistent with those of Ssewannyana et al. (2008) regarding the order of dominant shank colours among indigenous chickens in Uganda. The dominance of yellow tarsi in chickens has been reported elsewhere (Desta et al., 2013; Negassa et al., 2014; Hirwa et al., 2019). Contrarily, white tarsi are predominant in the Algerian chicken population (Dahloum et al., 2016). The pink and green-shank phenotypes in the present study were more frequently observed in cocks than in hens.

The variability of skin colours of the chicken body and shank is influenced by sex, genotype, and physiological state and is particularly prominent in laying hens (Eriksson et al., 2008). Dark-pigmented or dull (dark) skins are observed in poor layers; moreover, during the laying period, yellow pigments from the body of laying hens are diverted to the egg yolks (Singh, 2022). Hence, the combined effect of these factors and the subjective colour determination used in the present study may explain the higher frequency of yellow shanks in the hens, which otherwise commonly exhibited dull skins.

The predominant eye colour was orange, followed by yellow and brown. Dark-green, pink, dark-red, and cyan/blue variants of eye colour were rarely observed. These findings confirm an earlier report of the dominance of orange-eyed chickens in Uganda (Ssewannyana et al., 2008). A similar finding was also reported in Algerian indigenous chickens (Dahloum et al., 2016).

Regarding earlobe colour, our findings were contrary to those of Ssewannyana et al. (2008), who reported no particular colour occurring in the earlobes of hens in Uganda. The hens in the present study showed wide variations in prominent earlobe colours. Our observations of red or pink earlobe blemished with white, yellow, or cyan/blue are consistent with observations in Ethiopia (Desta et al., 2013). The red (light and dark variants) dominant earlobe colour variant in this study is consistent with reports in Ethiopian village chicken populations (Dana et al., 2010; Desta et al., 2013). The observed proportions of white earlobes were much lower than in previous reports in Uganda (Ssewannyana et al., 2008), Algeria (Dahloum et al., 2016), and Rwanda (Hirwa et al., 2019). Earlobe colour traits are reportedly sex-linked and polygenic (Wragg et al., 2012). Incidentally, the pink, green, cyan, and grey earlobe colours observed have not been previously characterised in Uganda. Thus, this study highlights the large variability of earlobe colours among indigenous Ugandan chickens. The hens were more likely than the cocks to have earlobe colours other than light red.

Similarly, the beak colours of the chickens varied widely. The hens mostly showed yellow (21.5%), brown (21.5%), grey (19.5%), and purple/brown-purple beaks, whereas cocks mainly showed grey (31.6%), yellow (18.4%), and brown (18.8%) beaks. Generally, the grey beak occurred more frequently in aggregate, followed by brown and yellow in equal proportions (25.4%, 20.1%, and 20.0% respectively). The proportion of green beak colour was higher than that in Rwandan chickens (Hirwa et al., 2019) whilst it was not reported in the Ethiopian population. The yellow to brownish beaks could be due to carotenoid pigmentation from xanthophylls in feed whilst the grey to dark beaks occur due to high melanin concentrations.

#### Head features of indigenous chickens in Uganda

The association of all descriptive head features of the Ugandan indigenous chickens (Supplementary Figure S15 and Table 6) with the sex category may be corroborative evidence of the sex-linked nature of earlobe shape and size, comb type as well as the presence of wattles and crests. The hens in general had typically small round, earlobes, and a single-type comb with small wattles. Cocks on the other hand typically presented large oval-shaped earlobes with single-type combs as well as large floppy wattles.

Despite the low incidence, hens across the regions were more likely than cocks to have tufted crests. This is consistent with the report by Desta et al. (2013) but contrary to the report of more tufted crest cocks than hens by Wang et al. (2012). Tuft crests in chickens are caused by a non-sex-linked dominant gene and it is considerably more noticeable in hens than in cocks. Therefore, having more tufted crest hens than cocks could be due to the farmer breeding practices of culling cocks earlier from their flock across the regions.

Comb type is regulated by two dominant genes, *R* and *P* for rose and pea combs, respectively. The absence of these dominant genes results in a single-comb type (Jacob, 2022). Therefore, the rose comb trait occurring at low proportions indicates a low frequency of the gene, consistent with reports elsewhere (Desta et al., 2013; Negassa et al., 2014; Dahloum et al., 2016; Brown et al., 2017).

The occurrence of hooked beaks supports the general observance of the curved beak in Rwandan indigenous chickens (Hirwa et al., 2019). Beak shapes play a vital role in scavenging for feed resources around the homestead. In the wild, avian beak conformation is influenced by adaption to behaviours such as preening, probing for food, feeding, killing small prey/pests, manipulating objects, and feeding offspring (Jacob, 2022). However, no studies have detailed the evolution and morphometrics of the beak of domestic chickens (Iqbal and Moss, 2021).

The low proportion of pea comb in the study may be related to its irrelevance in tropical climates as it is an adaptive trait for colder climates (Lee, 2009). However, comb and wattles have a significant function in reducing body temperature. This is consistent with the nearly universal occurrence of wattles (98.0% of hens and 100% of cocks, *p <* 0.001) and single-type comb (95.0% of hens and 93.4% of cocks), which was similar (*p >* 0.05) across regions. The majority of indigenous chickens bearing combs with only a few combless hens agrees with an earlier report in Uganda (Ssewannyana et al., 2008), although, rose, double, and pea combs were not featured in previous reports. The low proportions of the rose contrasted with the report of its dominance in Ethiopian indigenous cocks (Negassa et al., 2014). The dominance of the single comb type in both sexes, followed by rose combs, was also reported within the Kaffa Zone of Ethiopia (Tadele et al., 2018).

#### Skeletal variance in the Ugandan indigenous chicken population

A significant (*p <* 0.001) association was observed in the skeletal variance of chickens across the regions (Supplementary Table S6 and Figure 7; S17-19), which could be attributed to the disproportionately low frequency of polydactyl, dwarf, and rumpless traits. Polydactyl and rumpless phenotypes were restricted to Northern and Western Uganda, suggesting their localised acceptance. The skeletal variance of most Ugandan chickens, which was mainly normal in both sexes and with medium body frame and conformation, supports earlier reports of 62% medium body size and 25% small body size among Ugandan indigenous chickens (Ssewannyana et al., 2008). The hens mainly had medium to small or rudimentary body frames whilst cocks showed medium to long body frames across the regions. Chickens with larger body frames grow faster and yield more meat. Regarding spur size, spurs were not noticeable or were rudimentary (39.9%) on some hens with most (59%) having small spurs. The incidence of medium and large spurs was higher (*p <* 0.001) in cocks across the region. This contrasted with the report of a nearly universally absent spur in the Ethiopian indigenous chicken population (Fistum, 2016). Reviews of earlier studies on the inheritance of spur described its incidence as a secondary sex characteristic inhibited by the ovarian hormones in most hens. As such, hens with removed ovaries all grew spurs (Hutt, 1949). The sex-influenced nature of spur incidence is supported in other studies (Fairfull and Gowe, 1986; Oguntunji and Ayorinde, 2009; Egena et al., 2011). The tail length, which is made up of feathers for balance while walking and a steering mechanism while in flight, was mainly medium across the regions. The cocks were more likely to possess a long tail than the hens across the regions, suggesting the sex-linked nature of the trait.

#### Allele and phenotypic frequencies of major traits in the Ugandan indigenous chicken population

Despite the considerably low distribution of the major chicken strains in the Ugandan indigenous chicken population, the allele frequencies of the tufted crests (0.29), ptilopody (0.20), and rose comb (0.17) strains were consistent with the expected Mendelian proportions of 0.25; indicating their preference for selection by the farmers who kept them. This finding is contrary to the lower-than-expected allele frequencies of the tufted crest, ptilopody, and rose comb in the Algerian (Dahloum et al., 2016), Ghanaian (Mensah, 2016; Brown et al., 2017), and Nigerian indigenous chicken populations (Ikeobi et al., 2001). Meanwhile, the low frequencies of frizzle and polydactyl alleles, despite their association with heat stress adaptation and higher body weight, respectively, were suggestive of their endangerment, partly due to selection against their population and their neglect in breeding programmes in Uganda.

#### Morphometric traits of indigenous chicken strains in Uganda

The chicken strains did not significantly differ in body weight and linear measurements within the sex categories, except for taller comb height in the naked-neck cocks compared to the rose comb cocks. The marginally longest wattle length in naked-neck hens and cocks compared to the normal, frizzle, polydactyl, rose comb, and tufted crest strains could be the effect of the naked-neck gene on wattle length as a heat dissipation trait. The naked-neck gene interacts with the environment, thereby impacting the productive performance of the strain at high temperatures (Chen et al., 2008). Additionally, the shank circumferences were largest among the rose comb hens and cocks compared to the rest of the strains, consistent with the report by Adekoya et al. (2013). This suggests local physiologic adaptation to the prevailing production system in Uganda like in other African countries; however, the mechanism is unclear since the rose comb gene is considered to be of European origin (Ikeobi et al., 2001). Amongst hens, the feathered-shank (ptilopody) strain was marginally heavier, with a larger thorax circumference and longer comb length compared to the normal, naked-neck, frizzle, polydactyl, and rose comb strains. However, thigh length was on the upper limit in the frizzle chickens compared to the rest of the strains. Meanwhile, amongst the cocks, the naked-neck strain showed marginally heavier body weight compared to the rest of the strains, while the thorax circumference and thigh length were on the upper limit for the tufted crest and feathered shank strains. The estimates of the effect of the chicken strains on the body weight and linear measurements recorded in this study are comparable to those in previous reports (Mensah, 2016; Brown et al., 2017; Machete et al., 2017). In contrast, higher body weight has been reported in naked-neck chickens compared to the other chicken strains (Njenga, 2005; Birteeb et al., 2016). The favourable marginal estimates for the chicken strains under the prevailing low input condition could be explored further for their potential under improved keeping conditions.

The general strong phenotypic correlation coefficients for all pairs of morphometric variables (Supplementary Table S7) for the chicken strains in this study, except for CH and SC, CH and SL, CL and SL, and WL and TL of the hens corroborate the reports in most studies (Egena et al., 2011; Dahloum et al., 2016; Hirwa et al., 2019; Otecko et al., 2019). In addition, the correlation coefficients for all pairs of morphometric traits were much stronger in cocks than in hens, with only LW and TC and CH and TC pairs comparable, which follows among other various selective forces and the evolution of sexual dimorphism in chickens (Karubian and Swaddle, 2001). This provides a good proxy for the estimation of the live body weight of chickens based on other morphometric values, especially those that indicate intrinsic body size.

## Conclusion

The results of this study revealed a great pool of production and phenotypic diversity in the genetic resources of Ugandan indigenous chicken typically reared in extensive scavenging, mixed crop-livestock production system to support household livelihoods; even in smallholdings of a little over 20 chickens. The vital roles of indigenous chickens in Uganda were further emphasised in a society of only 20.8% female-headed households in which 40.6% of women were responsible for keeping indigenous chickens. This forms part of a balanced system mainly focused on income generation from sales and household food sources. The production performance in terms of egg production of the hens was about 40.3 ± 0.6 eggs/year, while the weights of mature chickens at sale averaged 1.535 kg and 2.256 kg, respectively, for hens and cocks. The eggs produced in most indigenous-chicken-keeping undertakings were usually consumed within the household or incubated to hatch the replacement stock. Despite their low production performance, partly from the challenges imposed by the husbandry practices, indigenous chickens in Uganda were hardy and in good body condition to offer multipurpose functions, including serving as capital for the acquisition of other livestock flocks, mainly goats. As such, improvements in management practices and healthcare and supplementary feeding, in addition to selection within the indigenous chickens for increased productivity and conservation under the prevailing environment, could help increase their productivity.

Wide phenotypic variation was observed in all the traits studied as well as a generally strong phenotypic correlation between all pairs of morphometric variables measured, especially for those of the cocks. However, only marginally higher estimates in the morphometric traits of the chicken strains were detected, which did not clearly infer higher performance, despite the notion of better production performance over the normal counterparts. The allele frequencies were higher among those characterising the major chicken strains including the tufted crest, rose comb, and ptilopody. This demonstrated their local acceptance and prospects for sustainable utilisation and conservation worthiness in Uganda. In contrast, the low gene frequencies of frizzle and polydactyl genes despite being associated with adaptability to a low-input management system and tropical environment, suggest their low preference and risk of being lost from the chicken genetic resources in Uganda. This situation calls for a scientific drive to ensure that such traits of adaptive essence are maintained to ensure their sustainable development, utilisation, management, and conservation. The use of molecular genetics techniques will be useful in confirming the phenotypic diversity.

## Data Availability

The original contributions presented in the study are included in the article/Supplementary Material. Further inquiries can be directed to the corresponding author.
