# Supplementary material for: Uganda chicken genetic resources: I. phenotypic and production characteristics
Source: Front Genet. 2023 Jan 24;13:1033031. doi: 10.3389/fgene.2022.1033031 (PMC9902952; doi:10.3389/fgene.2022.1033031)
Supplement: Supplementary file 1 [file Table6.DOC]

**Table S6:** Variations in skeletal variance in indigenous chickens in Uganda

| **Qualitative traits**  **[n (%)]** | **Northern** | | **Central** | | **Western** | | **Eastern** | | **Uganda** | | *χ^2^* | | |
| --- | --- | --- | --- | --- | --- | --- | --- | --- | --- | --- | --- | --- | --- |
|  | ***Hen***  n=75 | ***Cock***  n=73 | ***Hen***  n=58 | ***Cock***  n=54 | ***Hen***  n=78 | ***Cock***  n=75 | ***Hen***  n=87 | ***Cock***  n=86 | ***Hen***  n=***298*** | ***Cock***  n=***288*** | ***Sex*** | ***Region*** |  |
| ***Skeletal variance*** | | | | | | | | | | | 2.7^ns^ | 32.1^***^ | |
| Rumpless | 1(1.3) | NR | NR | NR | NR | 2(2.6) | NR | NR | 3(1.0) | NR |  |  |  |
| Dwarf | NR | NR | 1(1.7) | 1(1.9) | NR | 1(1.3) | NR | NR | 2(0.3) | 1(0.7) |  |  |  |
| Polydactyl | 4(5.3) | 6(8.2) | NR | NR | NR | 1(1.3) | NR | NR | 5(1.3) | 6(2.4) |  |  |  |
| Normal | 70(93.3) | 68(91.8) | 48(98.3) | 53(98.1) | 76(97.4) | 73(97.3) | 87(100) | 86(100) | 291(97.3) | 280(96.9) |  |  |  |
| ***Body frame*** | | | | | | | | | | | 126.1^***^ | 136.2^***^ | |
| Rudimentary | NR | NR | 12(20.7) | 1(1.9) | 46(59.0) | 11(14.7) | NR | 2(2.3) | 58(19.5) | 14(4.9) |  |  |  |
| Medium | 74(98.7) | 39(53.4) | 44(75.9) | 31(57.4) | 32(41.0) | 42(56.0) | 86(98.9) | 61(70.9) | 236(79.2) | 173(60.1) |  |  |  |
| Long | 1(1.3) | 34(46.6) | 2(3.4) | 22(40.7) | NR | 22(29.3) | 1(1.1) | 23(26.7) | 4(1.3) | 101(35.1) |  |  |  |
| ***Body conformation/ shape*** | | | | | | | | | |  | 131.3^**^ | 147.4^***^ | |
| Medium | 63(84.0) | 28(38.4) | 27(46.6) | 9(16.7) | 11(14.1) | 7(9.3) | 49(56.3) | 27(31.4) | 150(50.3) | 71(24.7) |  |  |  |
| Blocky-compact | 9(12.0) | NR | 30(51.7) | 25(46.3) | 67(85.9) | 44(58.7) | 35(40.2) | 32(37.2) | 141(47.3) | 101(35.1) |  |  |  |
| Tall-angular | 3(4.0) | 45(61.6) | 1(1.7) | 20(37.0) | NR | 24(32.0) | 3(3.4) | 27(31.4) | 7(2.3) | 116(40.3) |  |  |  |
| ***Spur size*** |  |  |  |  |  |  |  |  |  |  | 230.1^**^ | 19.1^*^ |  |
| Rudimentary | 21(28.0) | 5(6.8) | 14(24.1) | NR | 43(55.1) | 3(4.1) | 41(47.1) | 6(7.0) | 119(39.9) | 14(4.9) |  |  |  |
| Small | 51(68.0) | 22(30.1) | 43(74.1) | 24(44.4) | 33(42.3) | 32(42.7) | 41(47.1) | 31(36.0) | 168(56.4) | 109(37.8) |  |  |  |
| Medium | 3(4.0) | 29(39.7) | NR | 20(37.0) | 1(1.3) | 28(37.3) | 3(3.4) | 33(38.4) | 7(2.3) | 110(38.2) |  |  |  |
| Large | NR | 17(23.3) | 1(1.7) | 10(18.5) | 1(1.3) | 12(16.0) | 2(2.3) | 16(18.6) | 4(1.3) | 55(19.1) |  |  |  |
| ***Tail length*** | | | | | | | | | | | 98.3^***^ | 89.9^***^ | |
| Short | 9(12.0) | 9(12.3) | 34(58.6) | 9(16.7) | 40(51.3) | 10(13.3) | 13(14.9) | 8(9.3) | 96(32.2) | 36(12.5) |  |  |  |
| Medium | 52(69.3) | 16(21.9) | 24(41.4) | 30(55.6) | 38(48.7) | 46(61.3) | 72(82.8) | 54(62.8) | 186(62.4) | 146(50.7) |  |  |  |
| Long | 14(18.7) | 48(65.8) | NR | 15(27.8) | NR | 19(25.3) | 2(2.3) | 24(27.9) | 16(5.4) | 106(36.8) |  |  |  |
| ^*^*P<*0.05; ^**^*P<*0.01; ^***^*P<*0.001; ns = non-significant; χ^2^ = Chi – square test of fixed variables; n = Chickens sampled; %= Relative frequency; NR = not reported. | | | | | | | | | | | | | |
