# Supplementary figures and images for: Uganda chicken genetic resources: I. phenotypic and production characteristics
Source: Front Genet. 2023 Jan 24;13:1033031. doi: 10.3389/fgene.2022.1033031 (PMC9902952; doi:10.3389/fgene.2022.1033031)

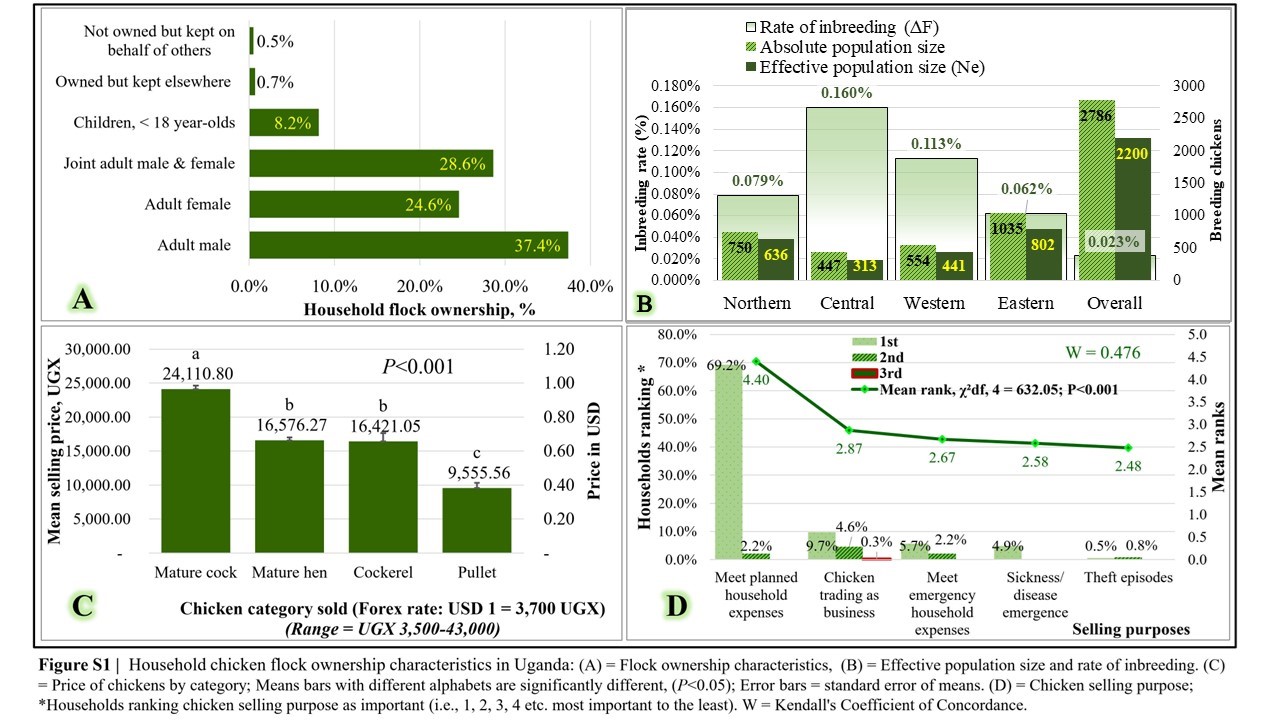

Supplement: Supplementary file 2 [file Presentation1.zip › Supplementary Figures 1 to19/Supplementary Figure S1.JPG]

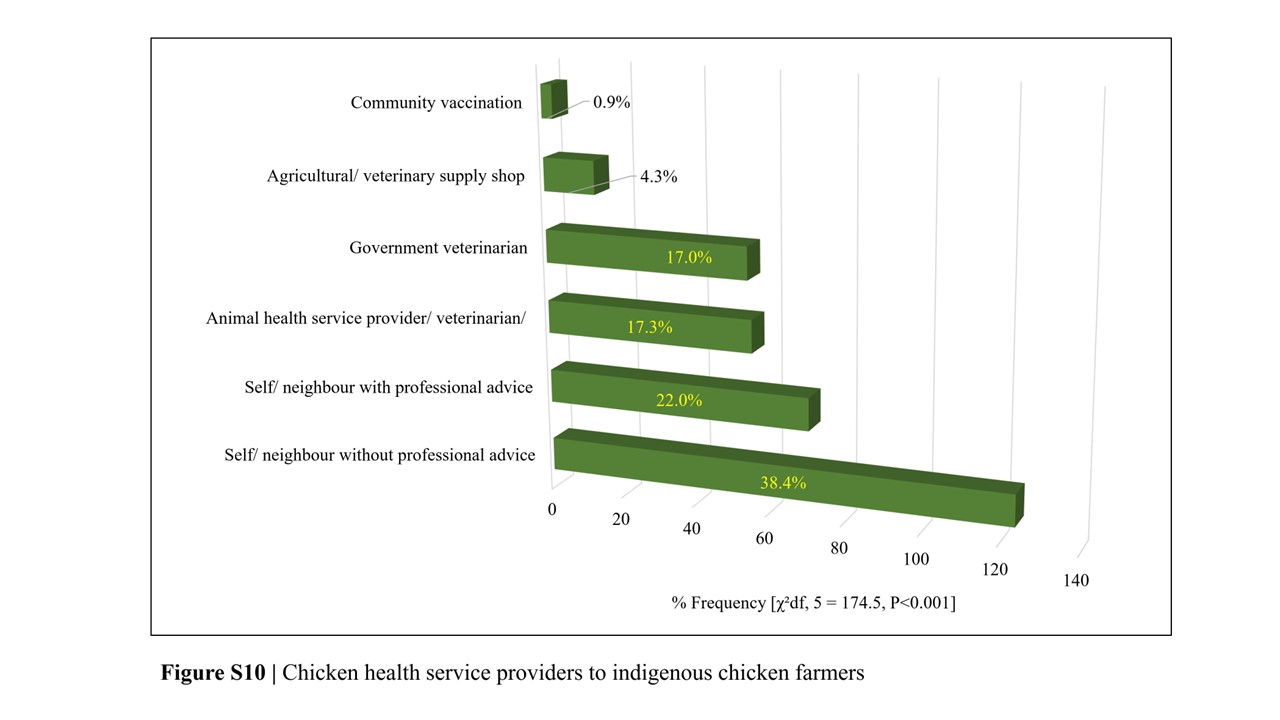

Supplement: Supplementary file 2 [file Presentation1.zip › Supplementary Figures 1 to19/Supplementary Figure S10.JPG]

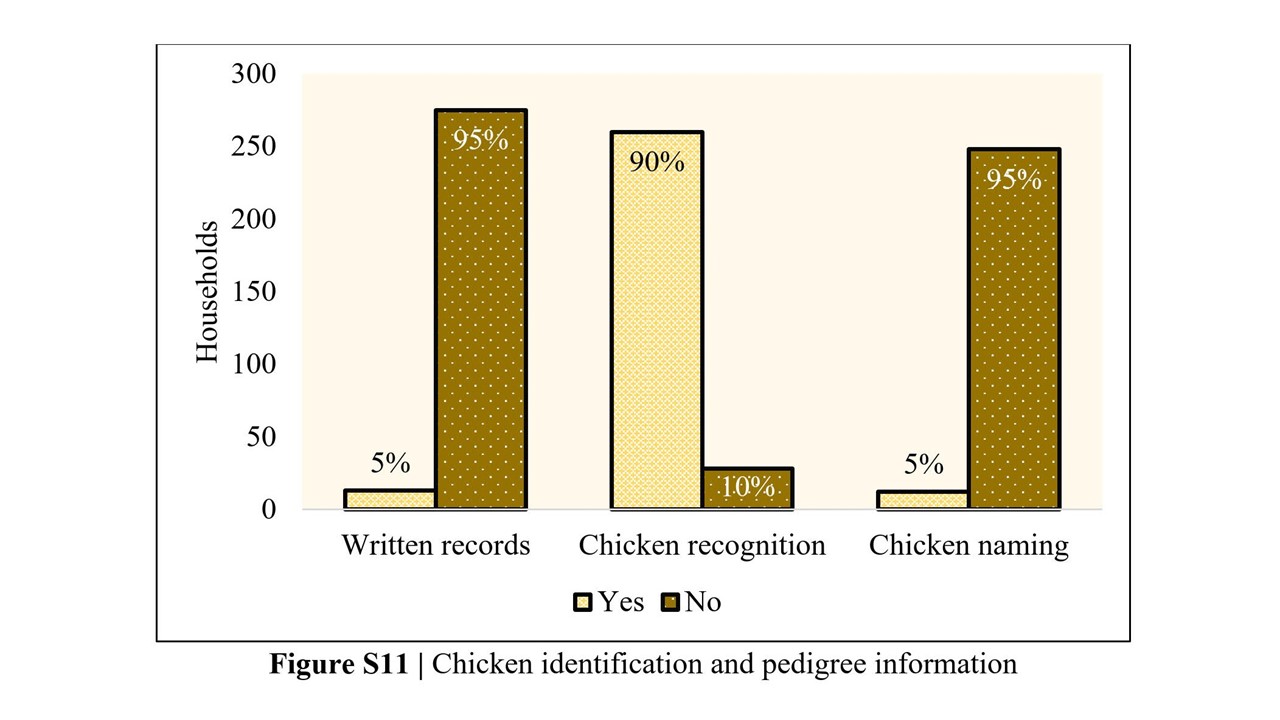

Supplement: Supplementary file 2 [file Presentation1.zip › Supplementary Figures 1 to19/Supplementary Figure S11.JPG]

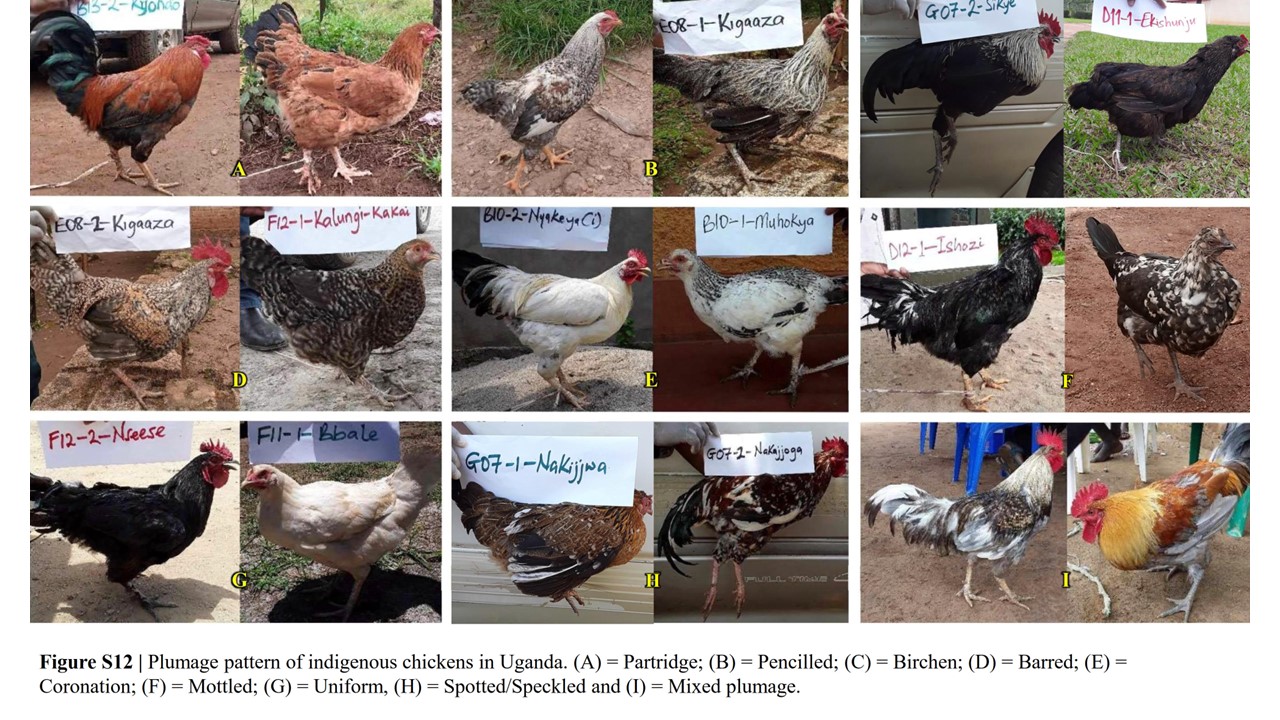

Supplement: Supplementary file 2 [file Presentation1.zip › Supplementary Figures 1 to19/Supplementary Figure S12.JPG]

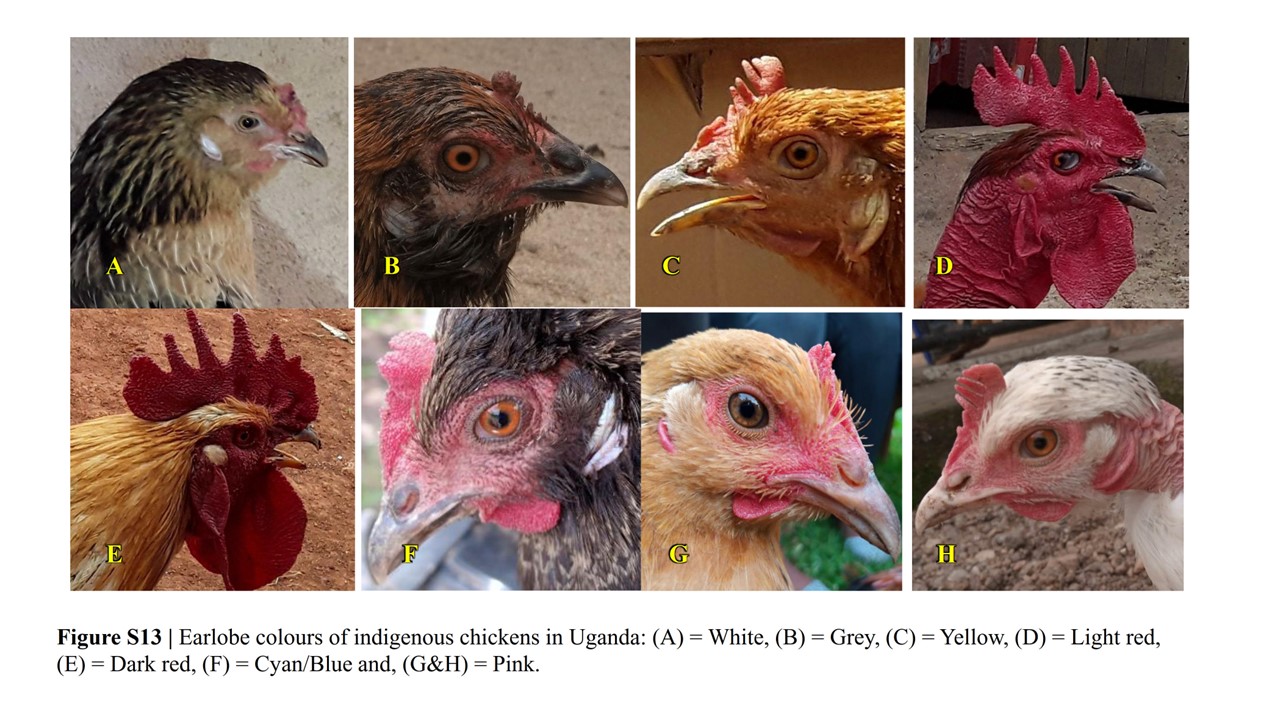

Supplement: Supplementary file 2 [file Presentation1.zip › Supplementary Figures 1 to19/Supplementary Figure S13.JPG]

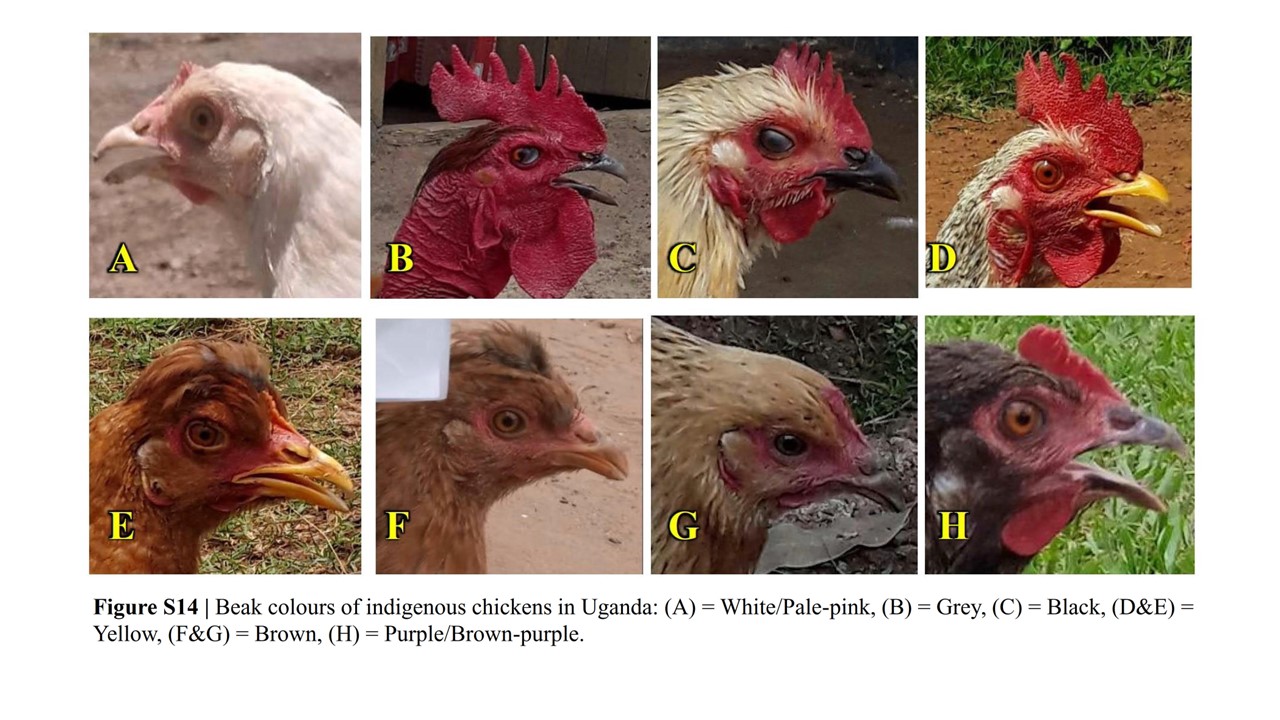

Supplement: Supplementary file 2 [file Presentation1.zip › Supplementary Figures 1 to19/Supplementary Figure S14.JPG]

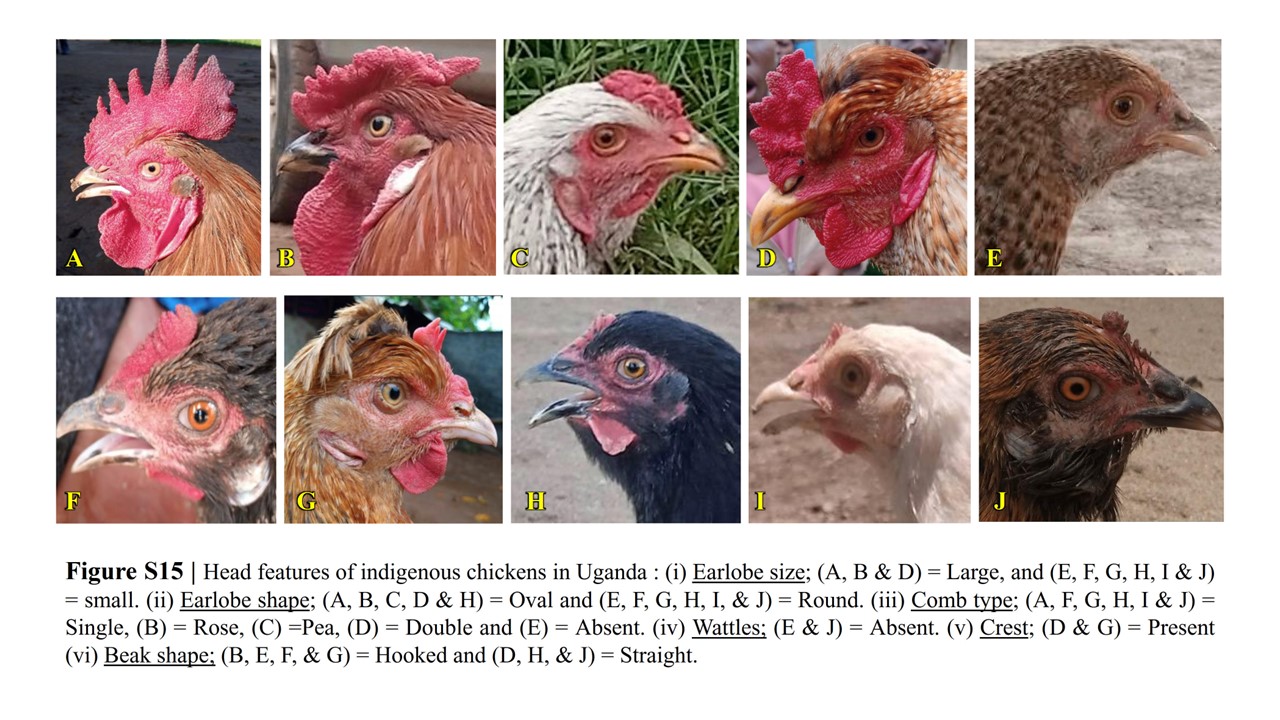

Supplement: Supplementary file 2 [file Presentation1.zip › Supplementary Figures 1 to19/Supplementary Figure S15.JPG]

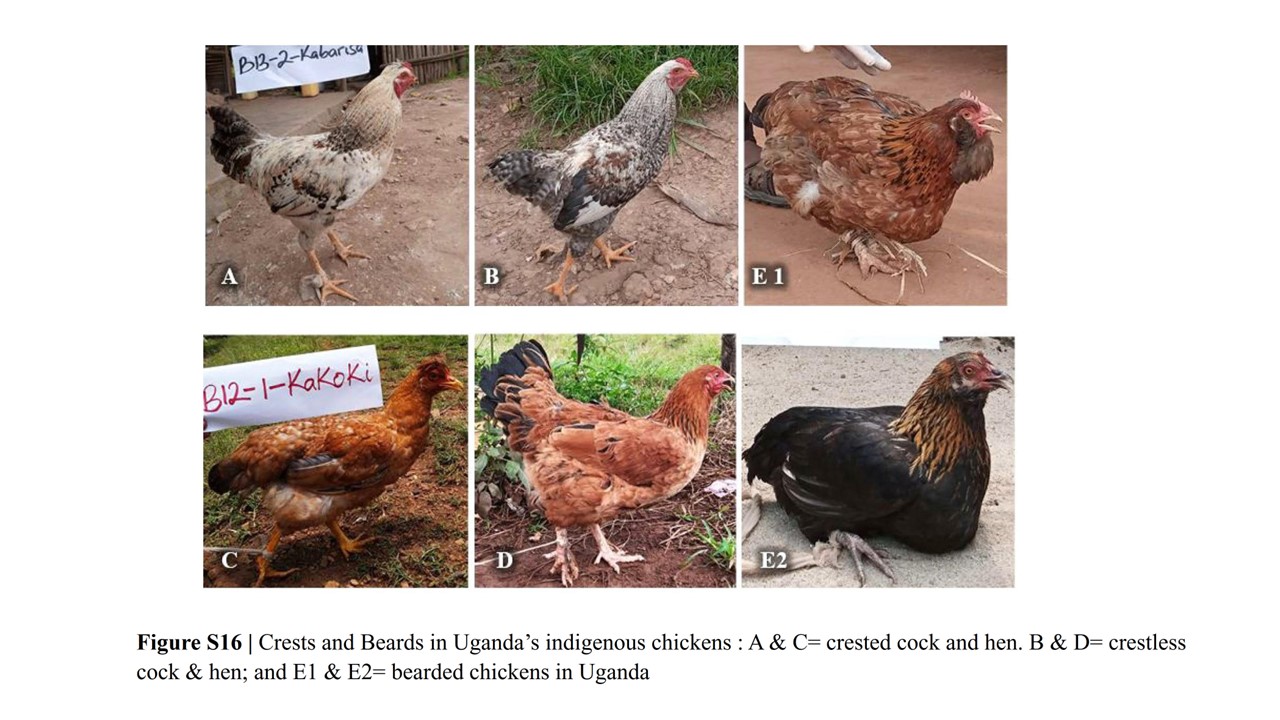

Supplement: Supplementary file 2 [file Presentation1.zip › Supplementary Figures 1 to19/Supplementary Figure S16.JPG]

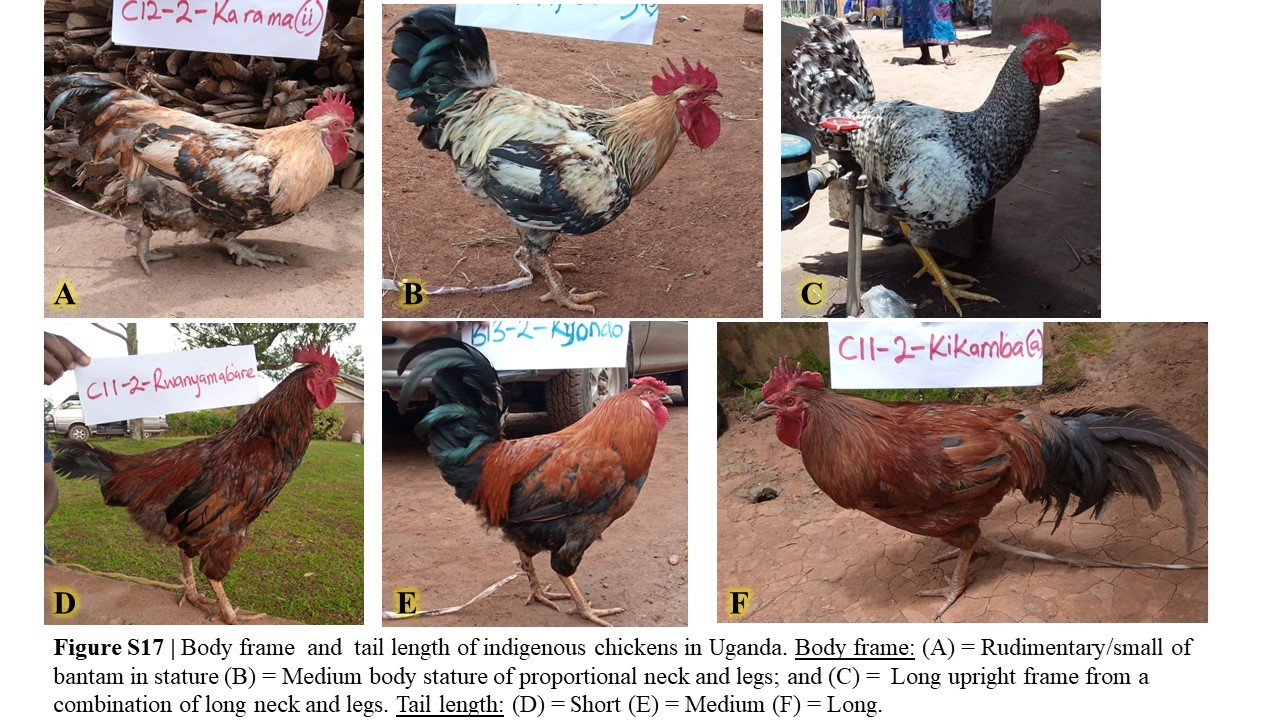

Supplement: Supplementary file 2 [file Presentation1.zip › Supplementary Figures 1 to19/Supplementary Figure S17.JPG]

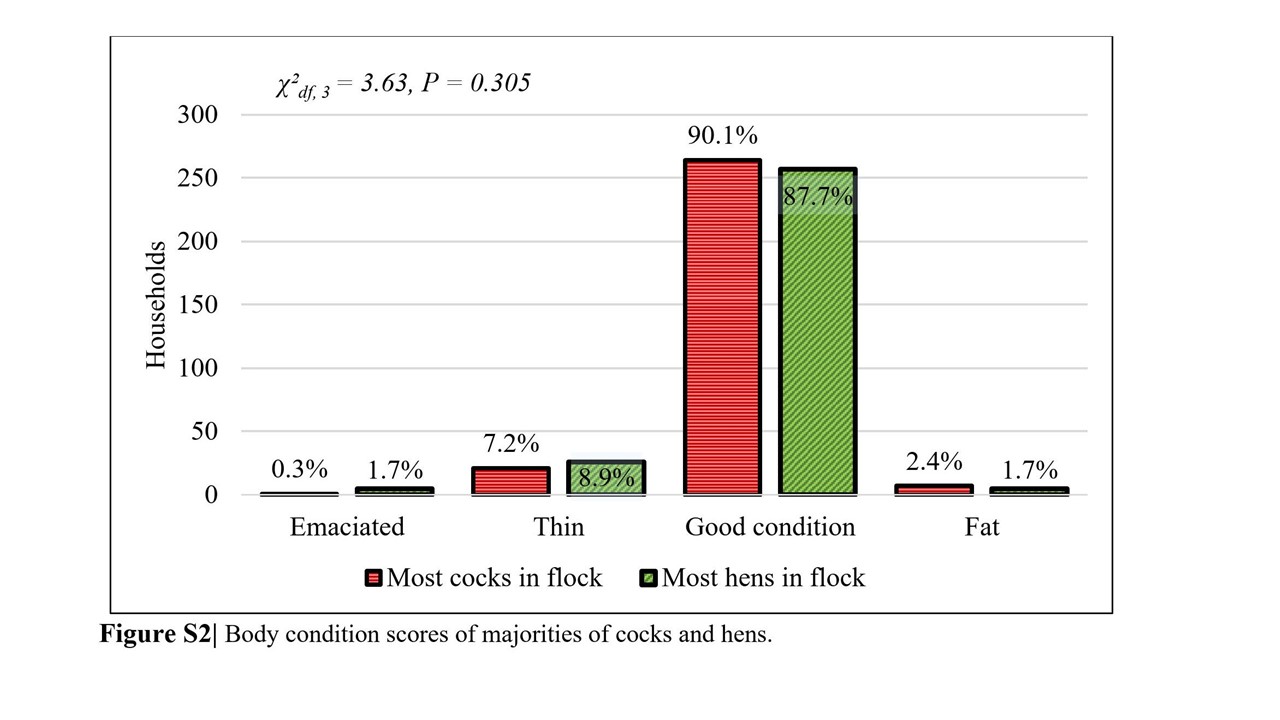

Supplement: Supplementary file 2 [file Presentation1.zip › Supplementary Figures 1 to19/Supplementary Figure S2.JPG]

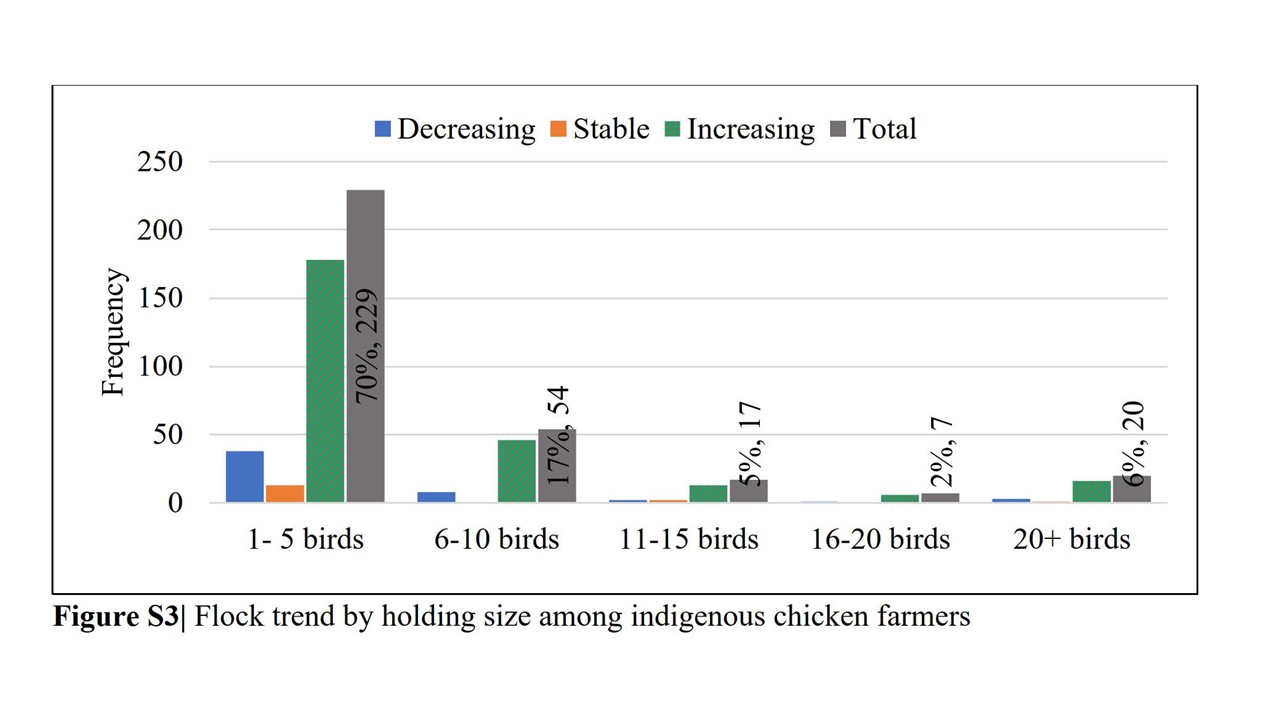

Supplement: Supplementary file 2 [file Presentation1.zip › Supplementary Figures 1 to19/Supplementary Figure S3.JPG]

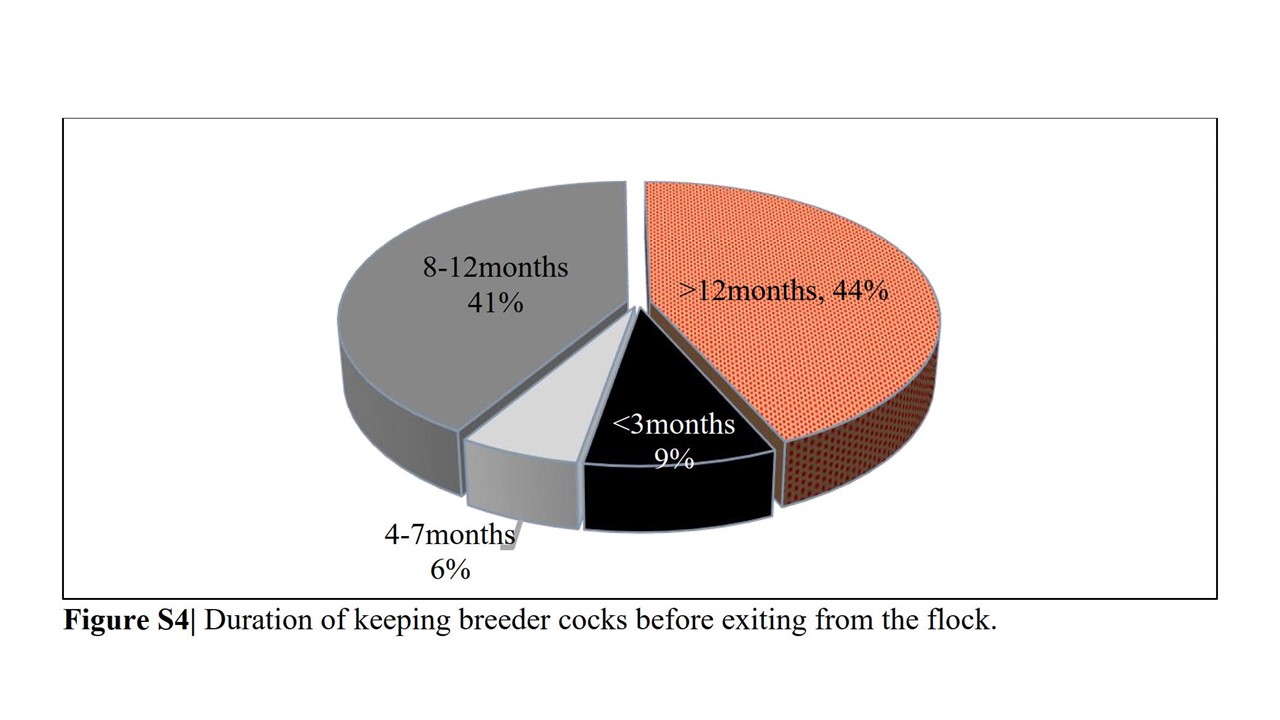

Supplement: Supplementary file 2 [file Presentation1.zip › Supplementary Figures 1 to19/Supplementary Figure S4.JPG]

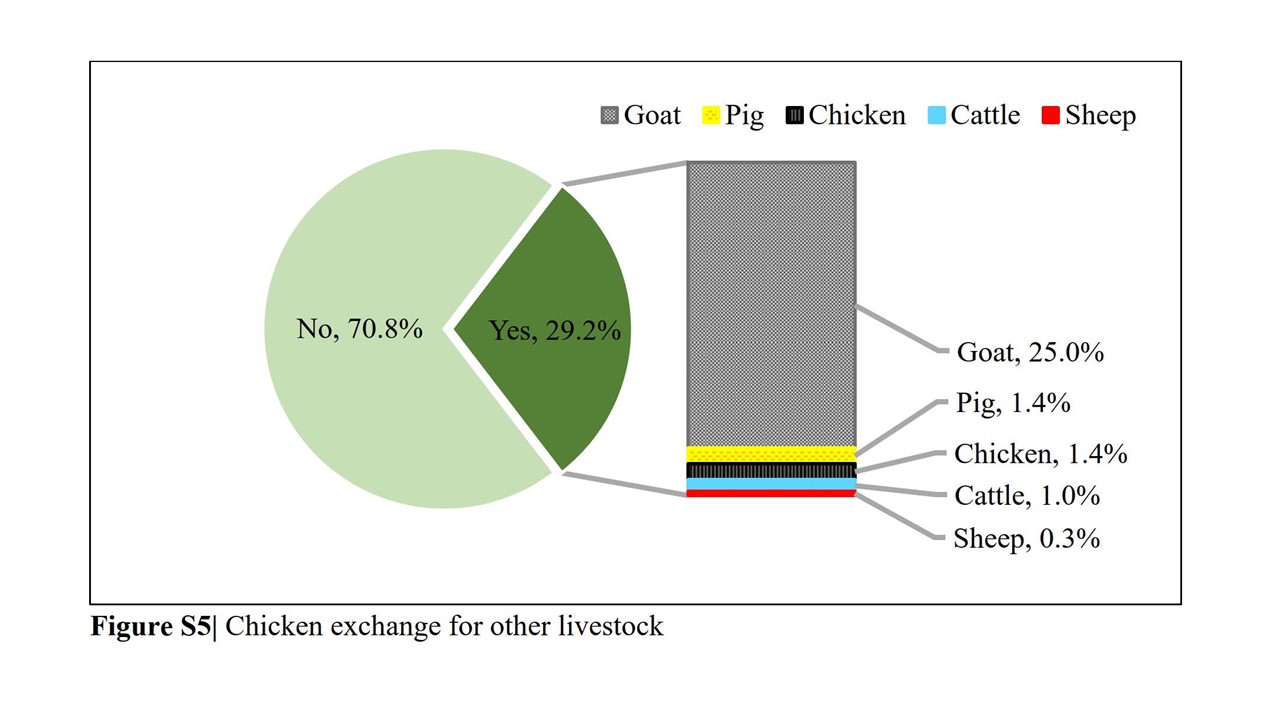

Supplement: Supplementary file 2 [file Presentation1.zip › Supplementary Figures 1 to19/Supplementary Figure S5.JPG]

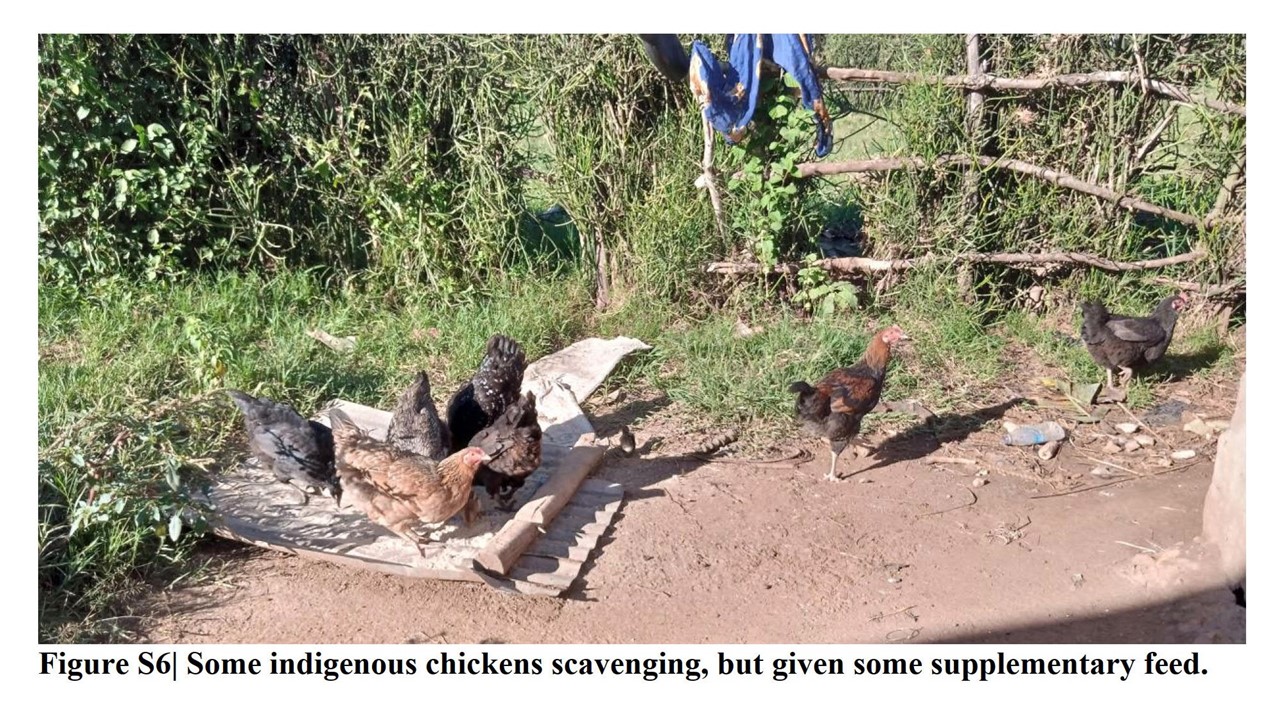

Supplement: Supplementary file 2 [file Presentation1.zip › Supplementary Figures 1 to19/Supplementary Figure S6.JPG]

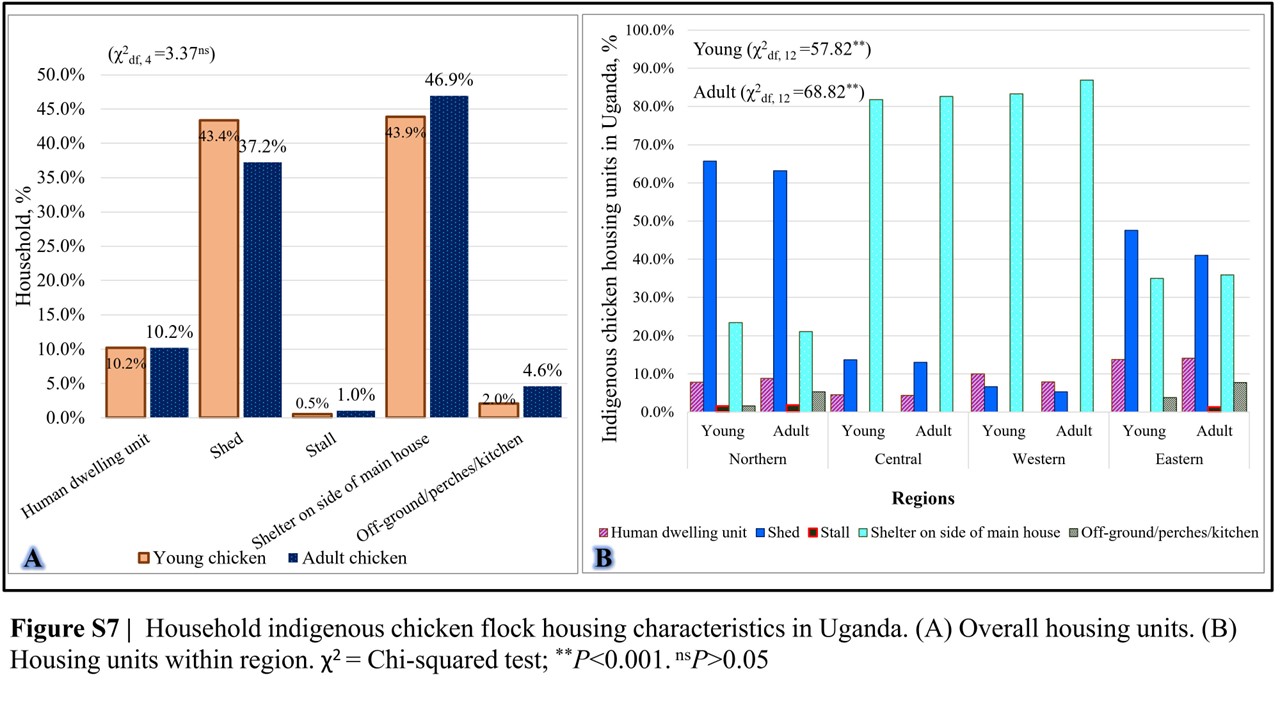

Supplement: Supplementary file 2 [file Presentation1.zip › Supplementary Figures 1 to19/Supplementary Figure S7.JPG]

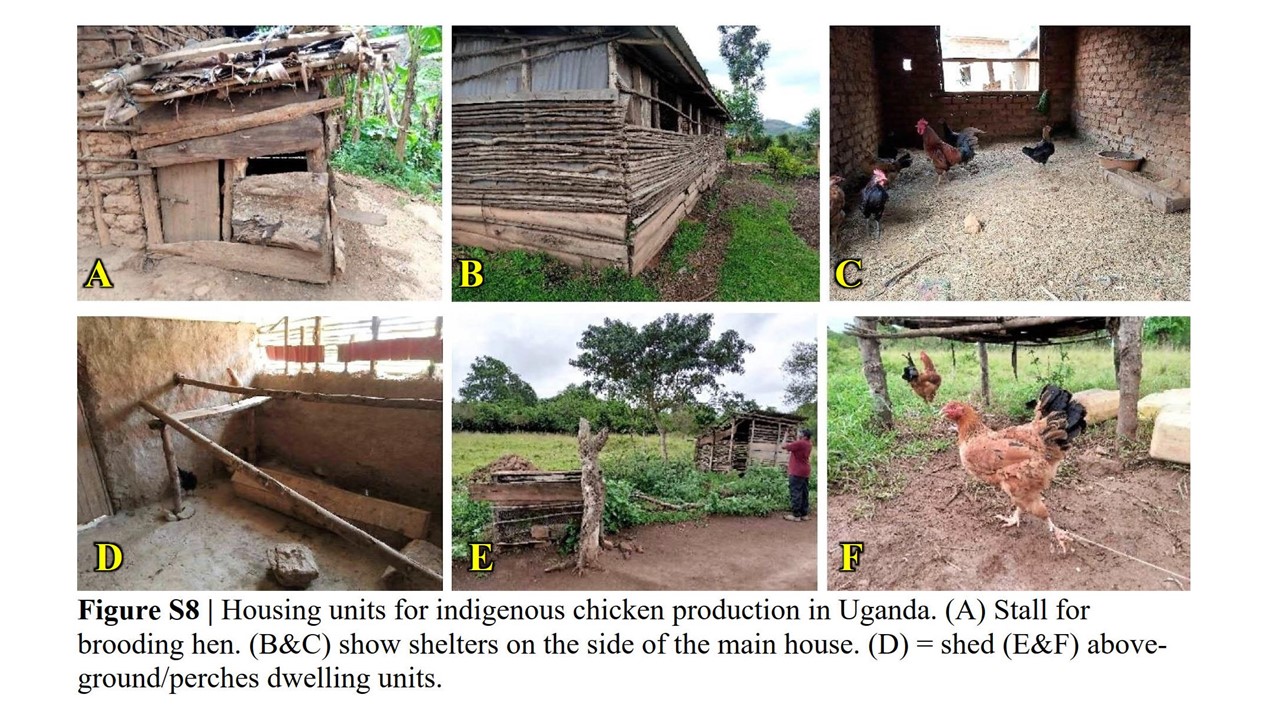

Supplement: Supplementary file 2 [file Presentation1.zip › Supplementary Figures 1 to19/Supplementary Figure S8.JPG]

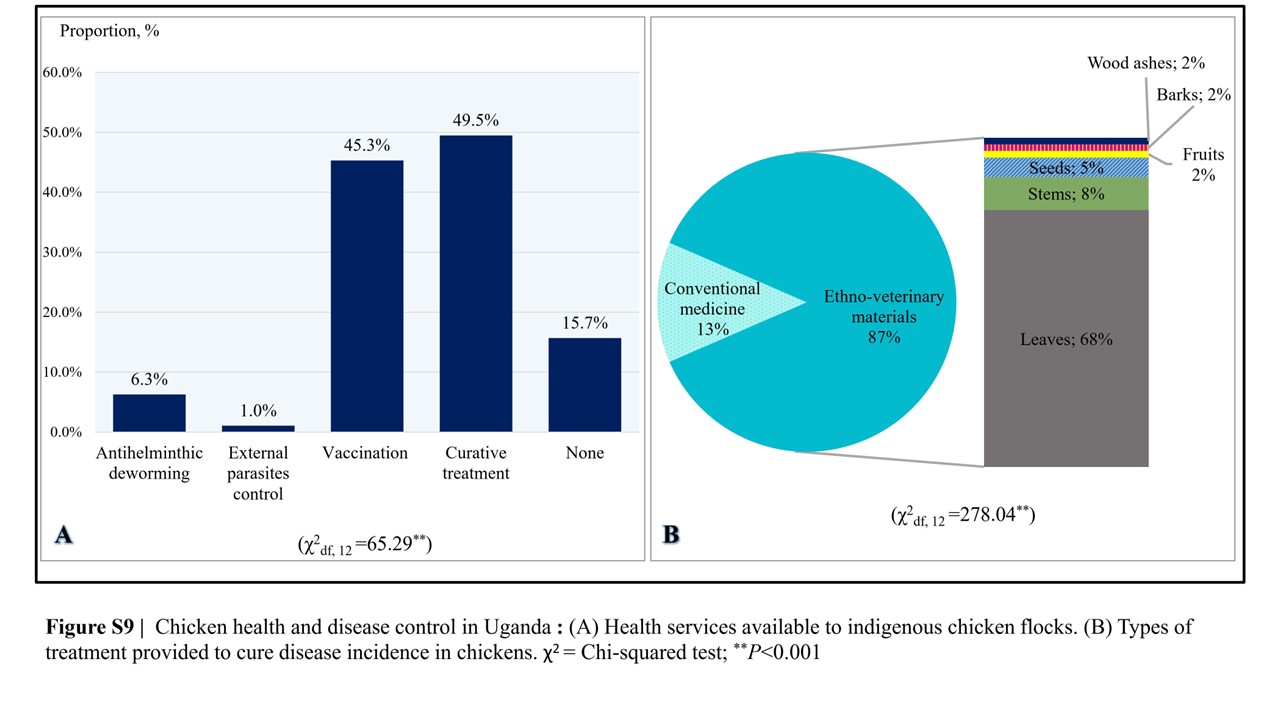

Supplement: Supplementary file 2 [file Presentation1.zip › Supplementary Figures 1 to19/Supplementary Figure S9.JPG]

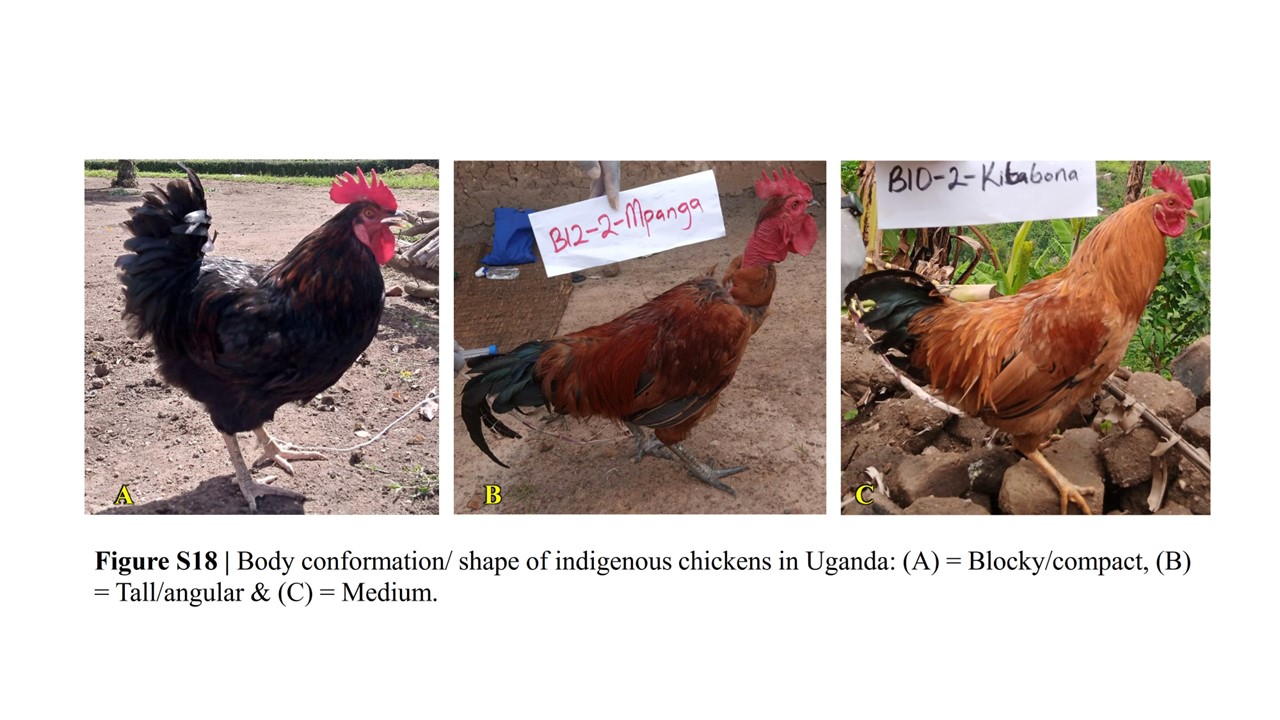

Supplement: Supplementary file 2 [file Presentation1.zip › Supplementary Figures 1 to19/Supplementary Figure18.JPG]

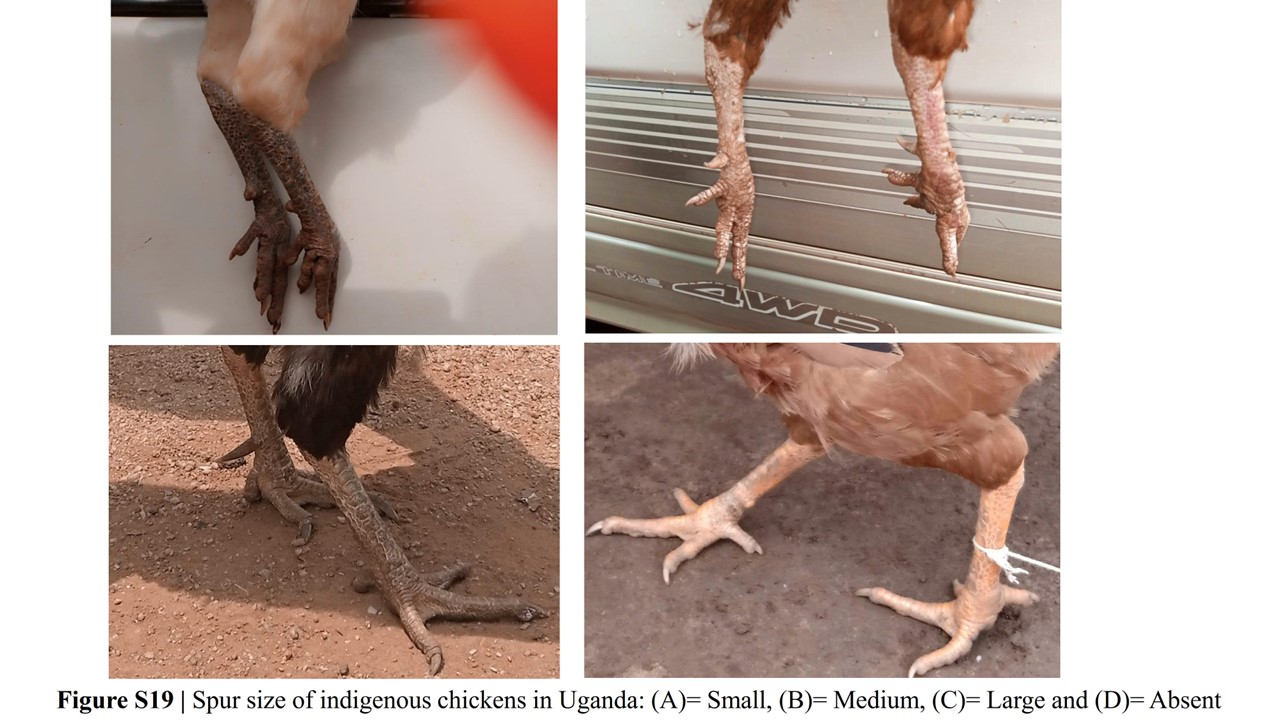

Supplement: Supplementary file 2 [file Presentation1.zip › Supplementary Figures 1 to19/Supplementary Figure19.JPG]
