# Supplementary material for: Uganda chicken genetic resources: I. phenotypic and production characteristics
Source: Front Genet. 2023 Jan 24;13:1033031. doi: 10.3389/fgene.2022.1033031 (PMC9902952; doi:10.3389/fgene.2022.1033031)

## Slide 1
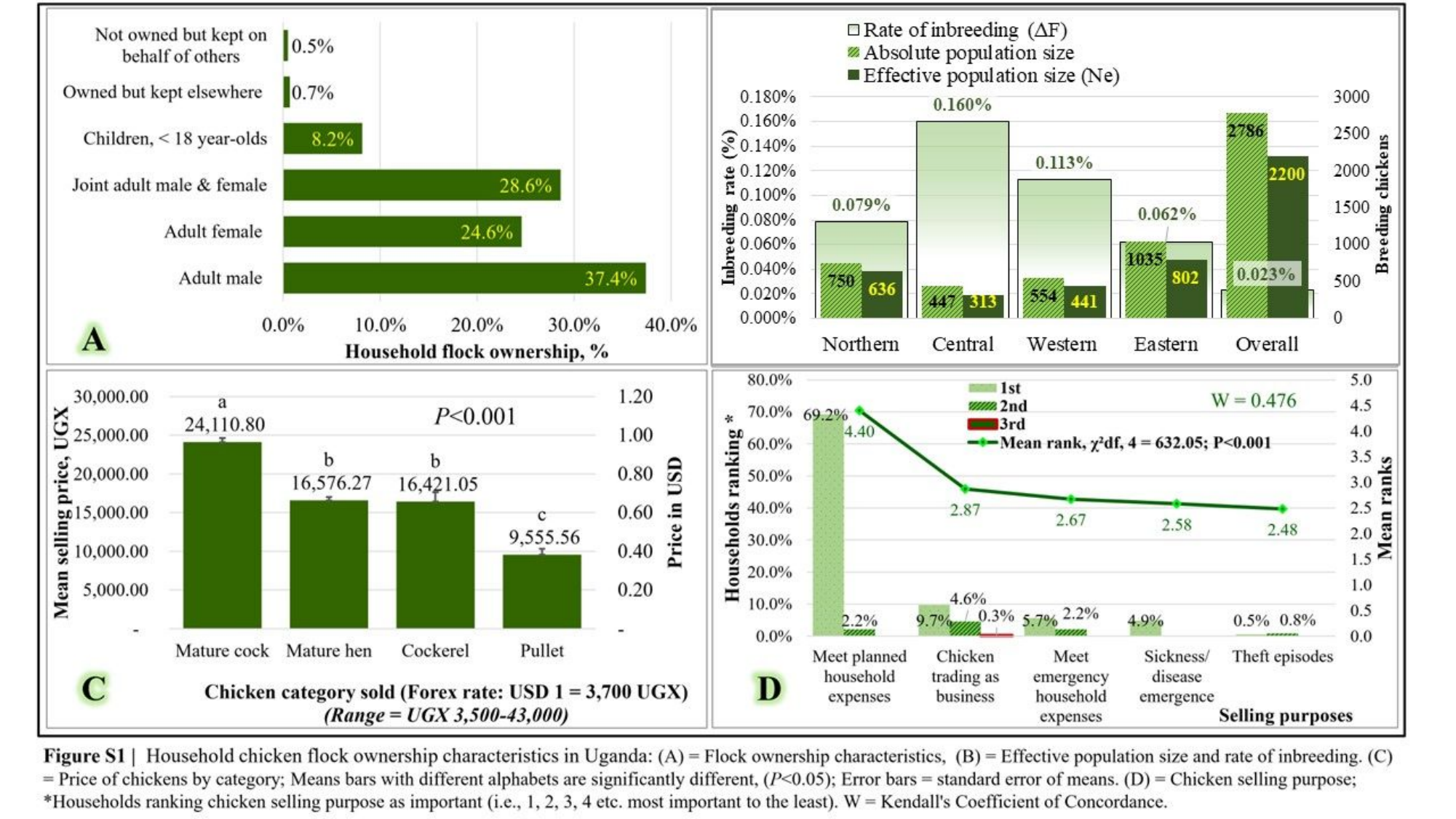

## Slide 2
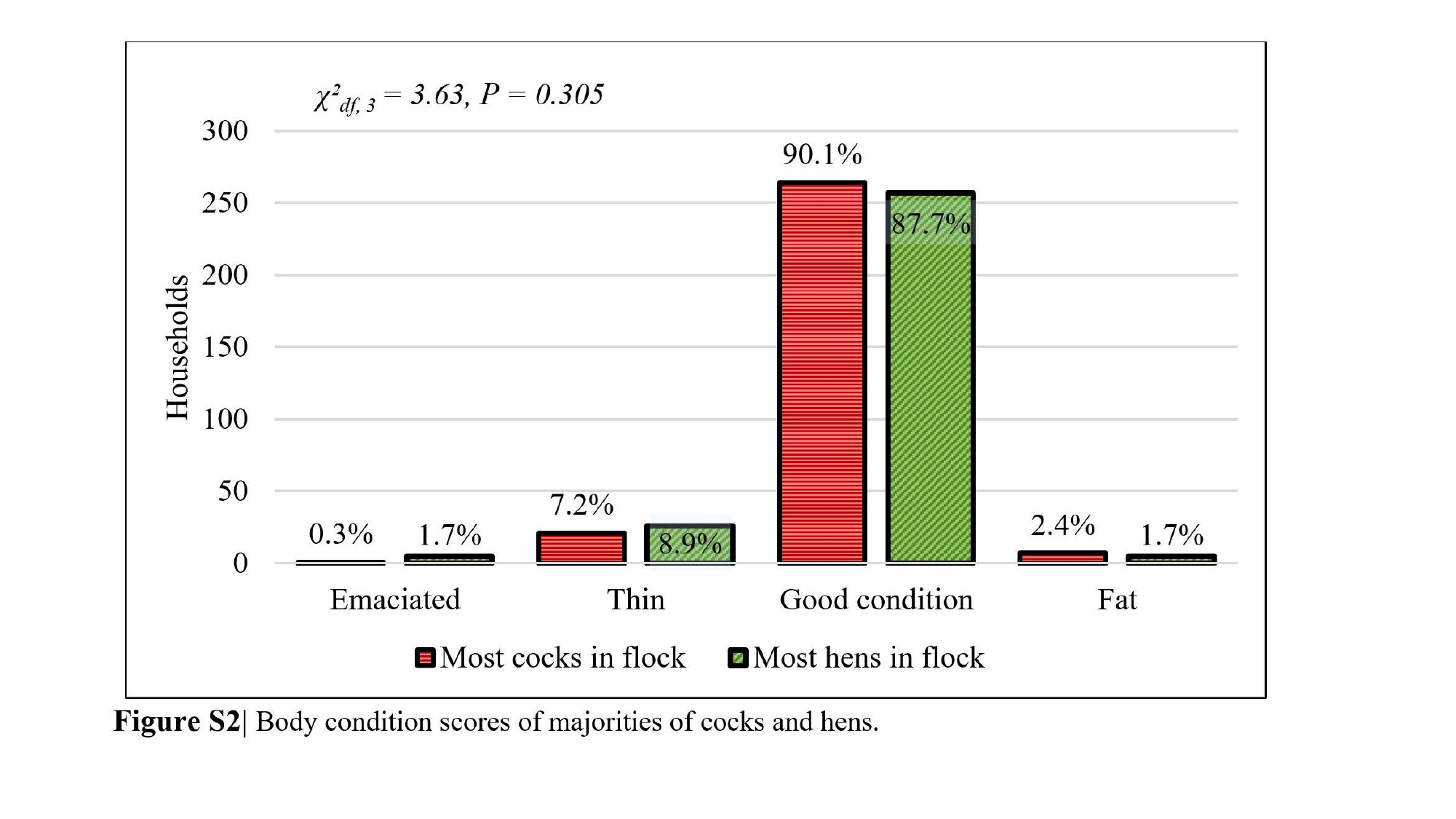

## Slide 3
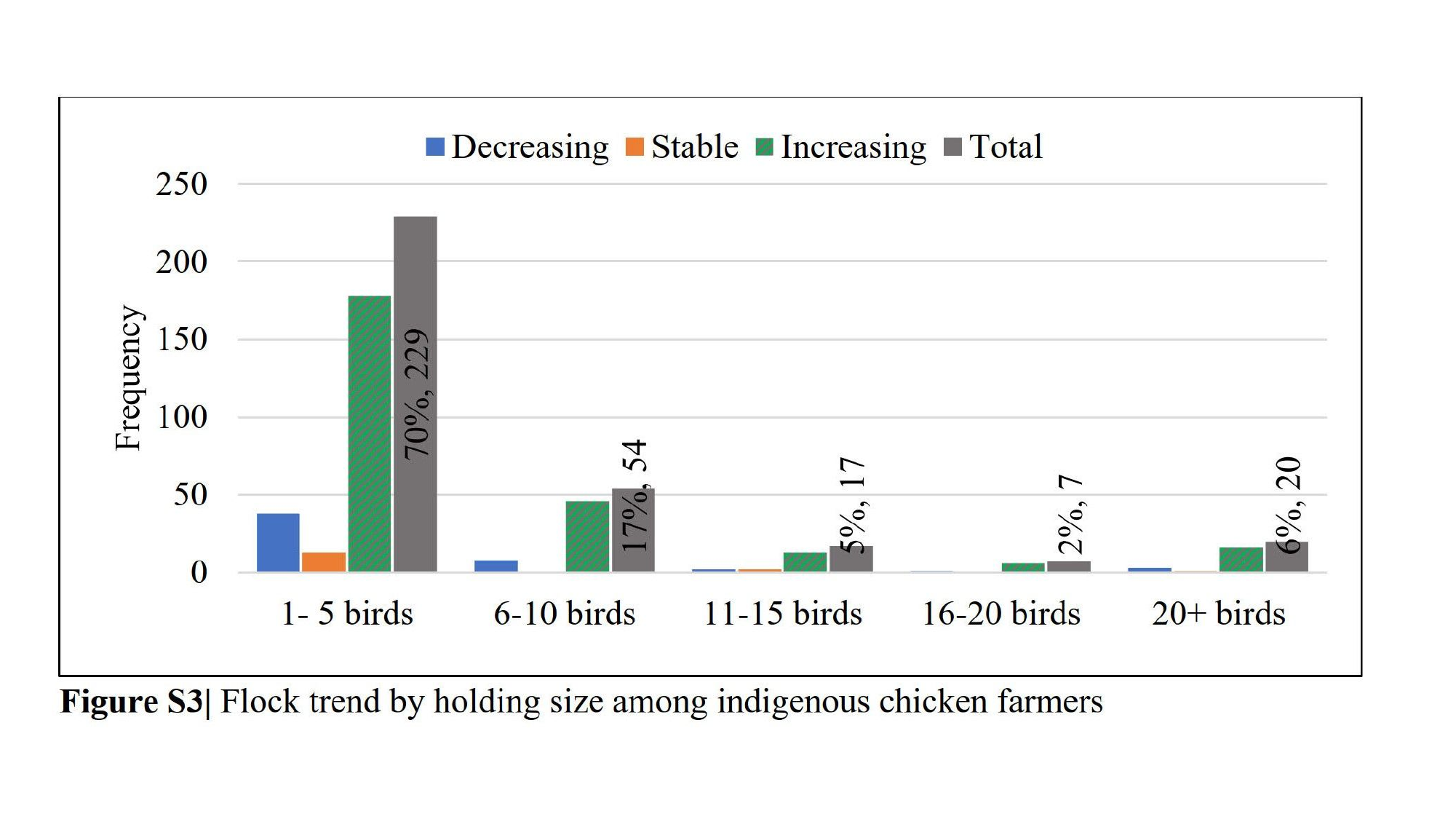

## Slide 4
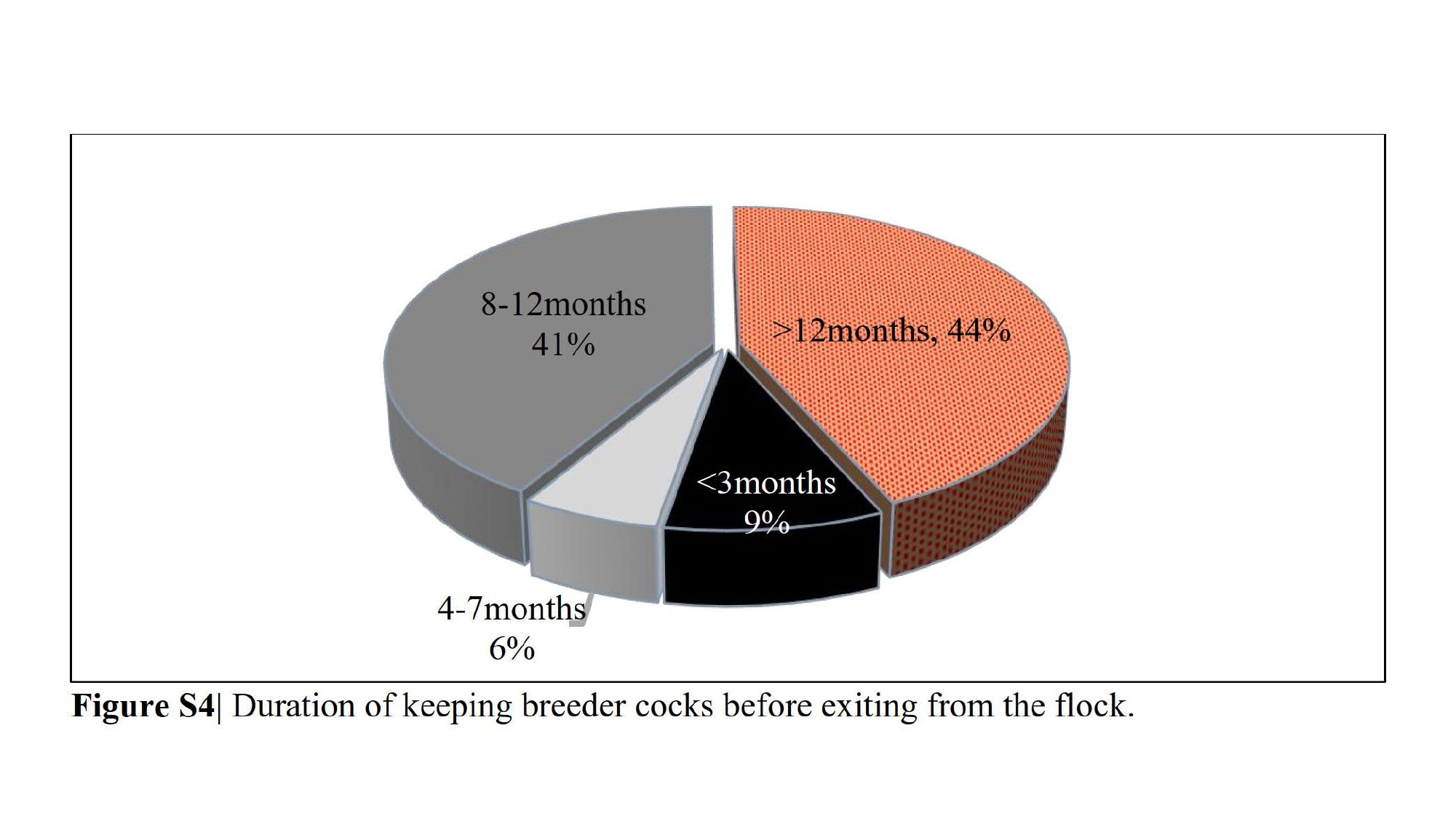

## Slide 5
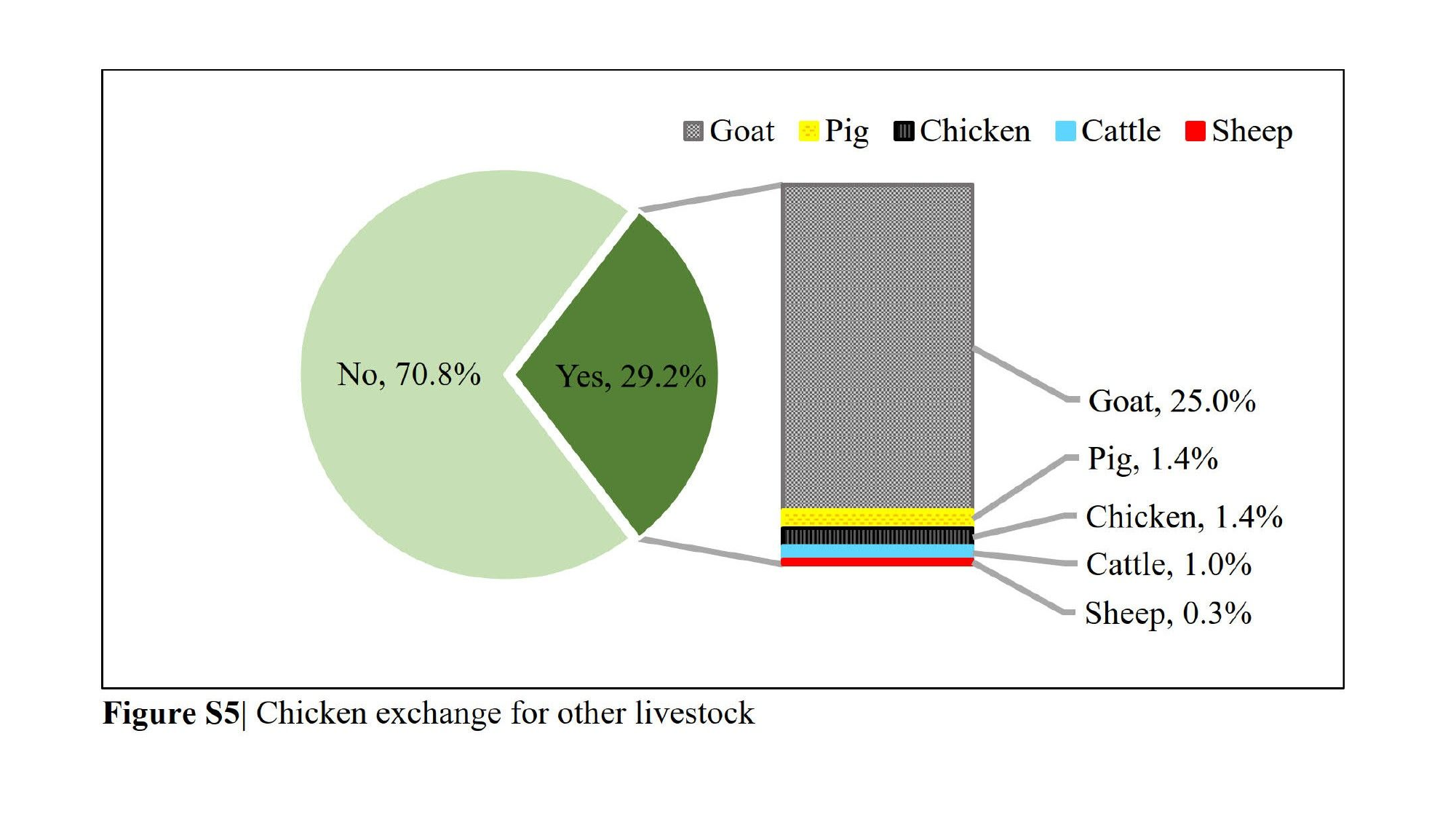

## Slide 6
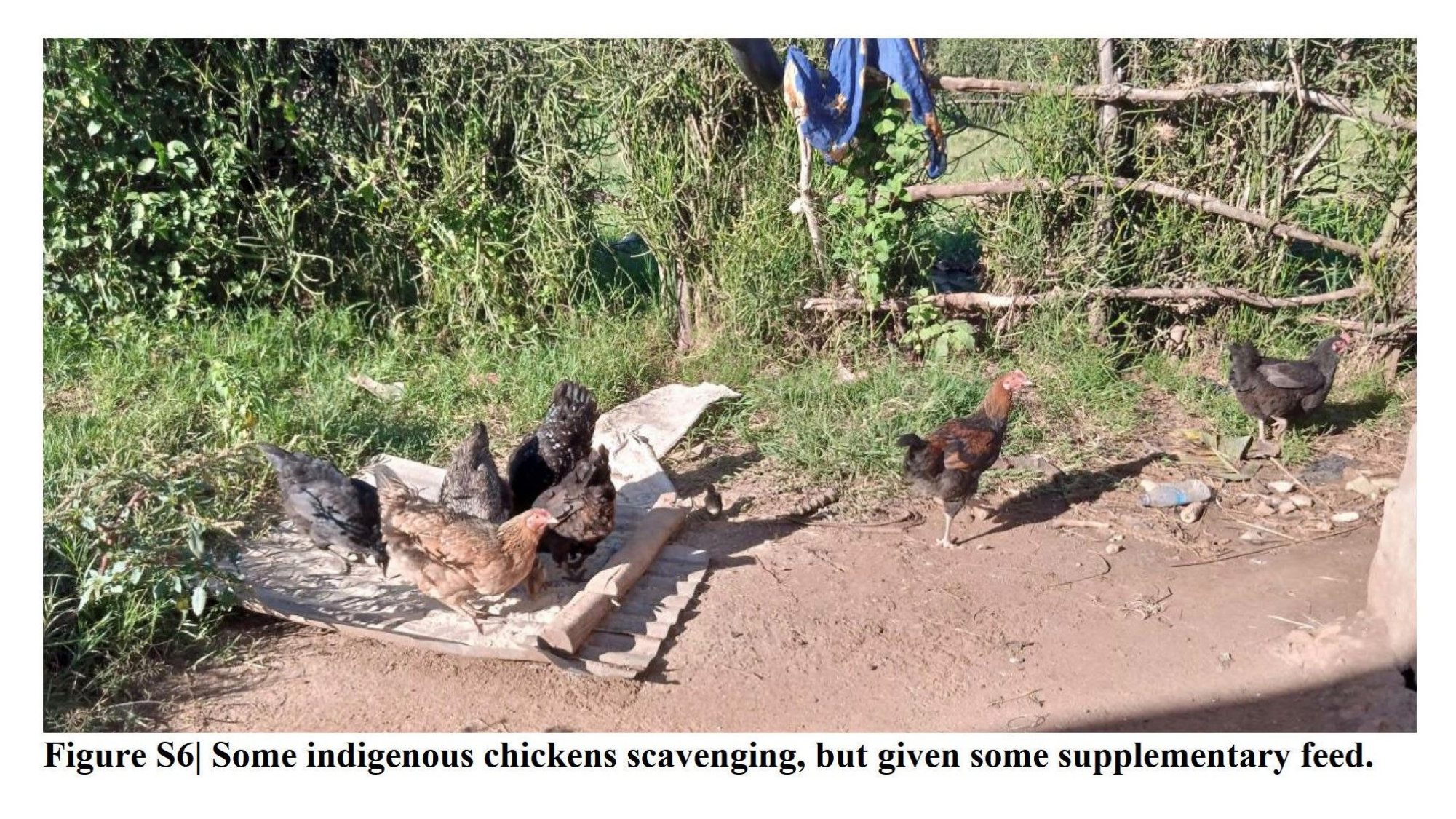

## Slide 7
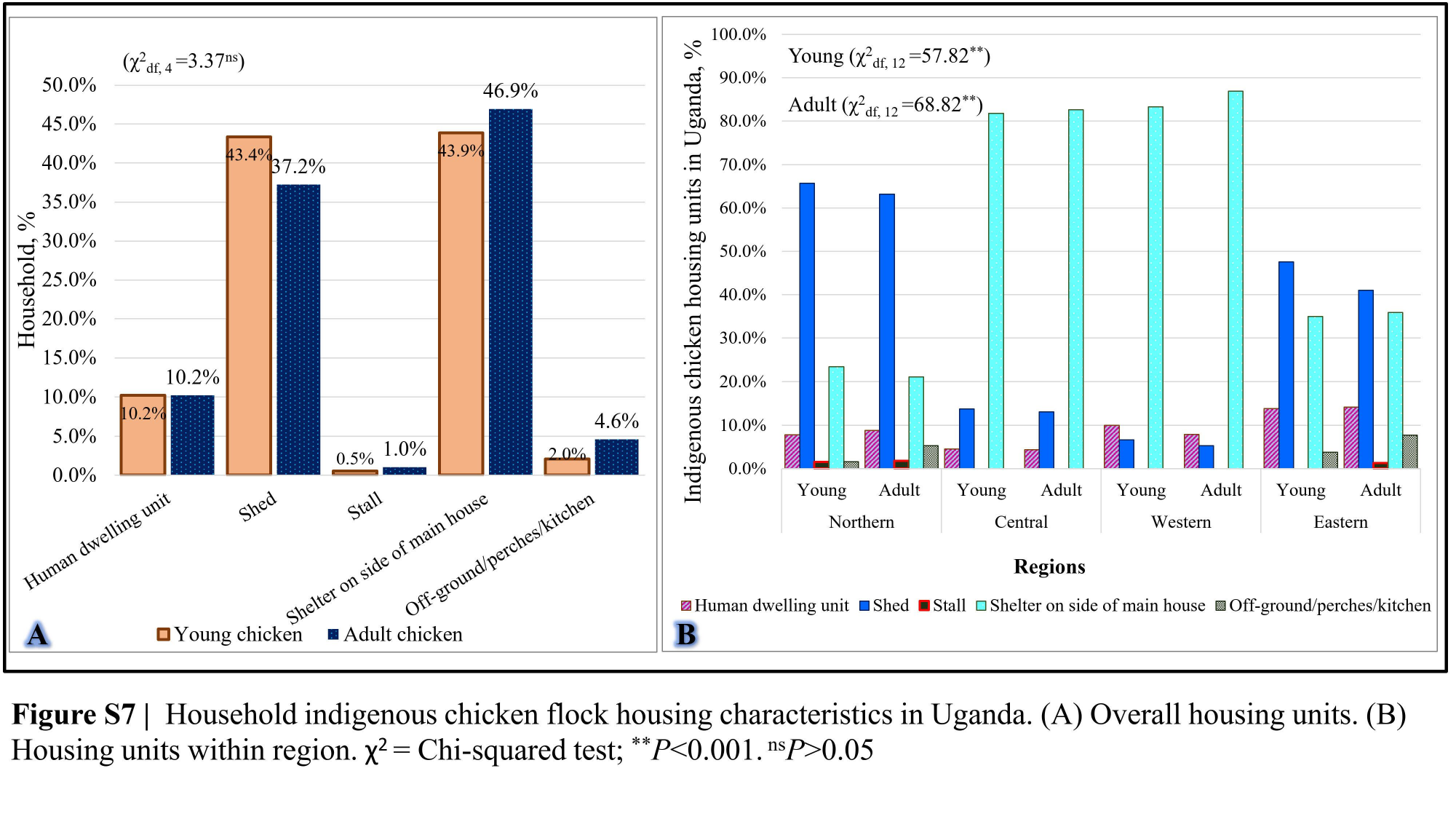

## Slide 8
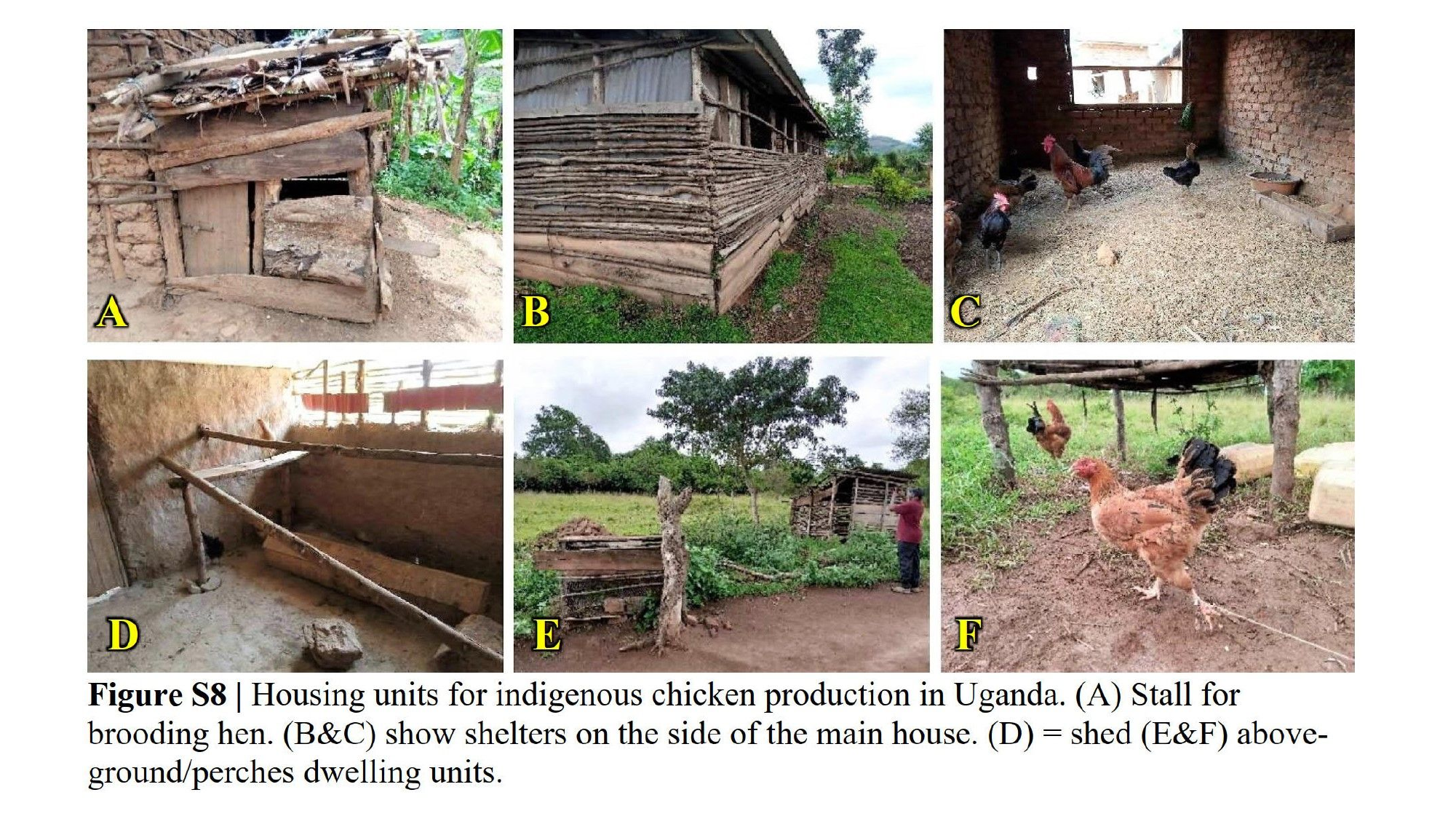

## Slide 9
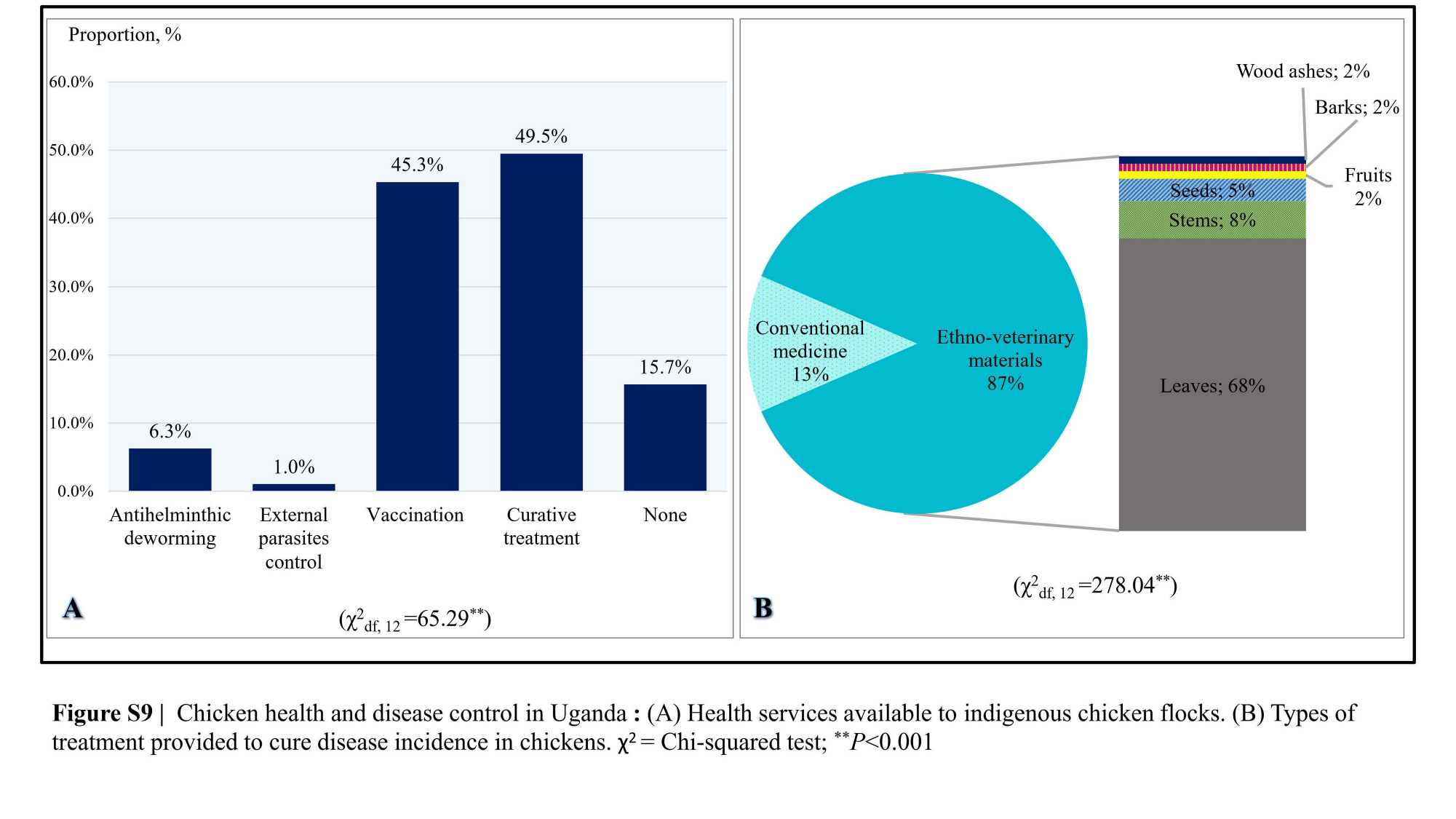

## Slide 10
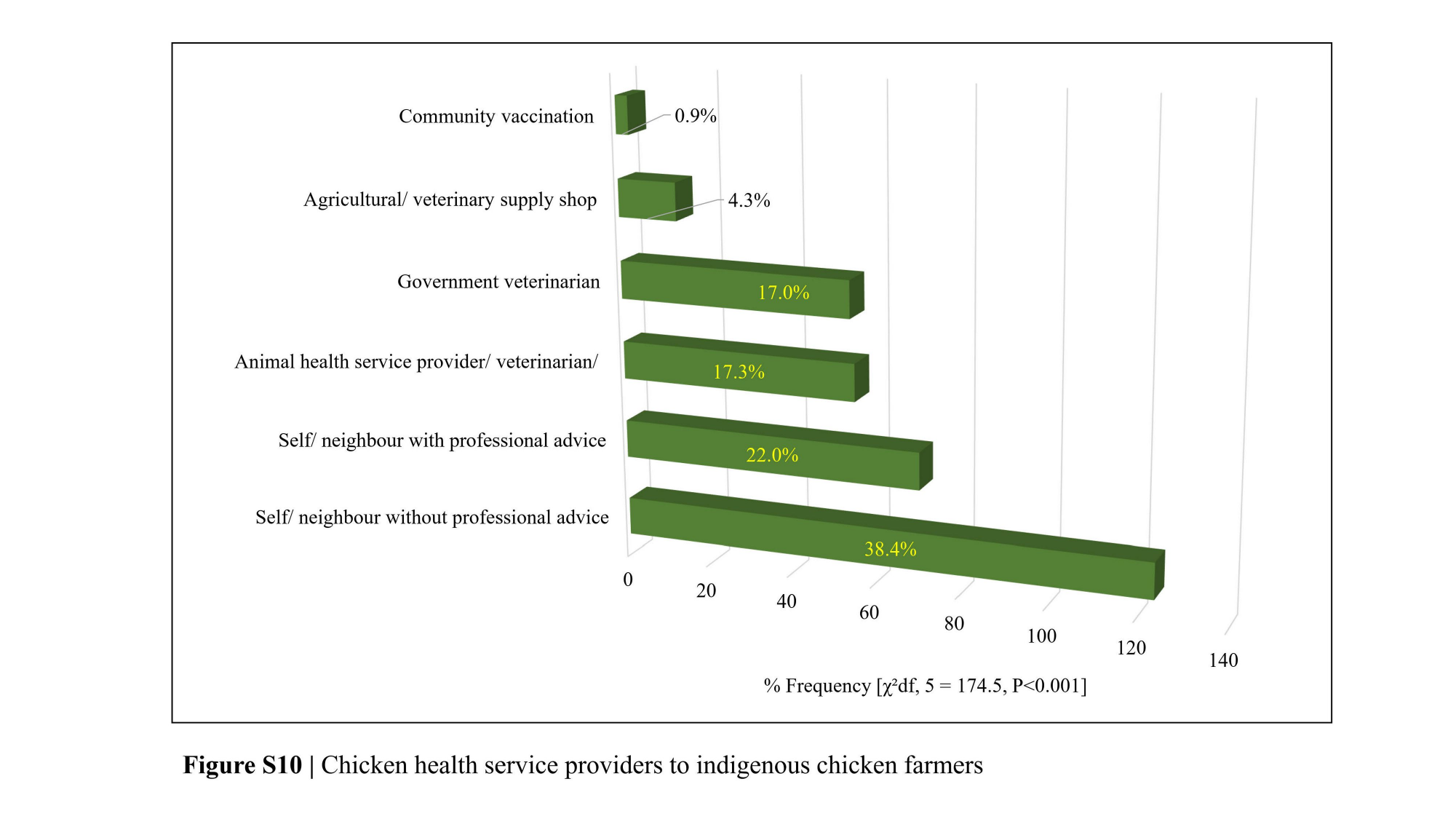

## Slide 11
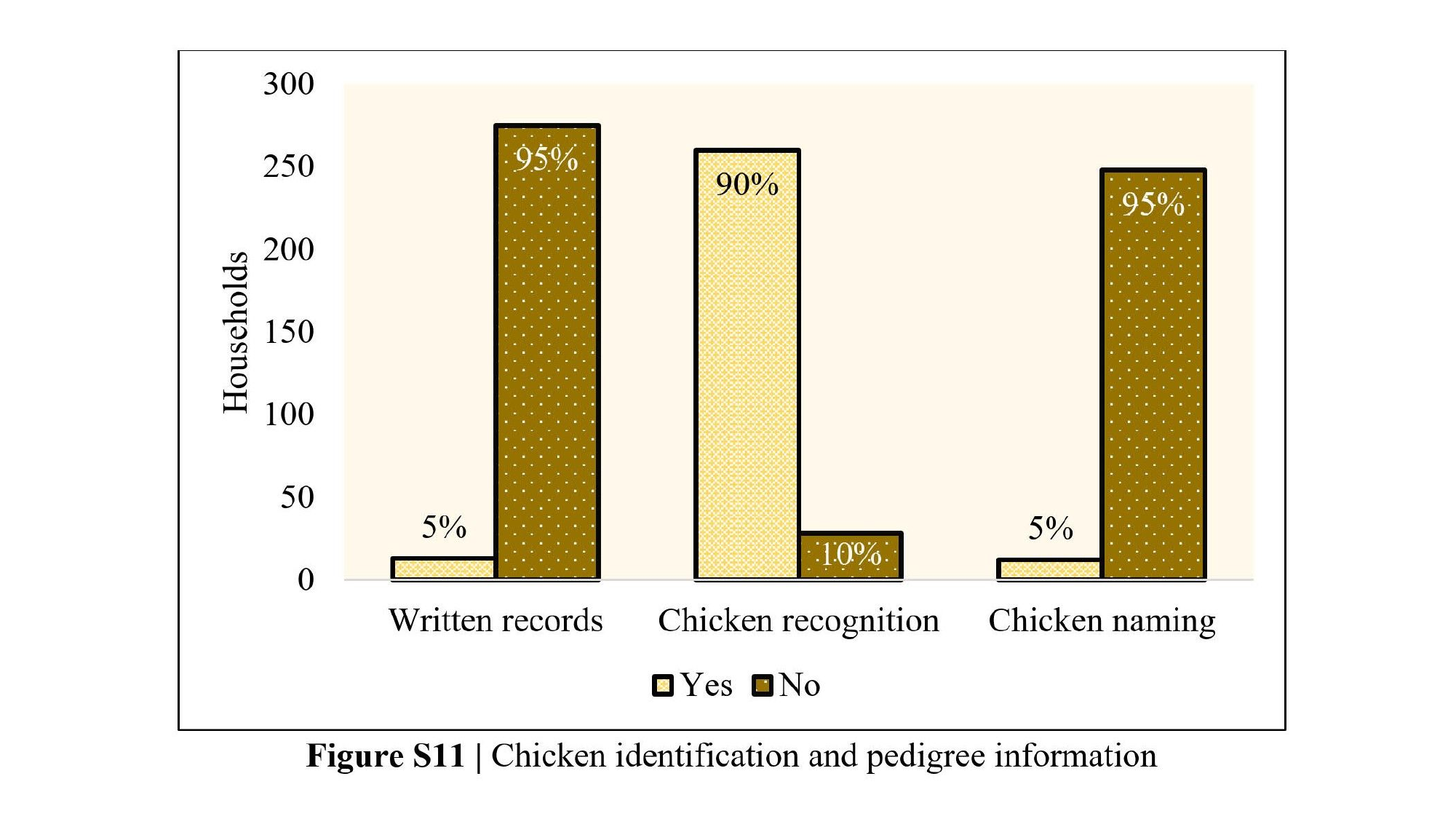

## Slide 12
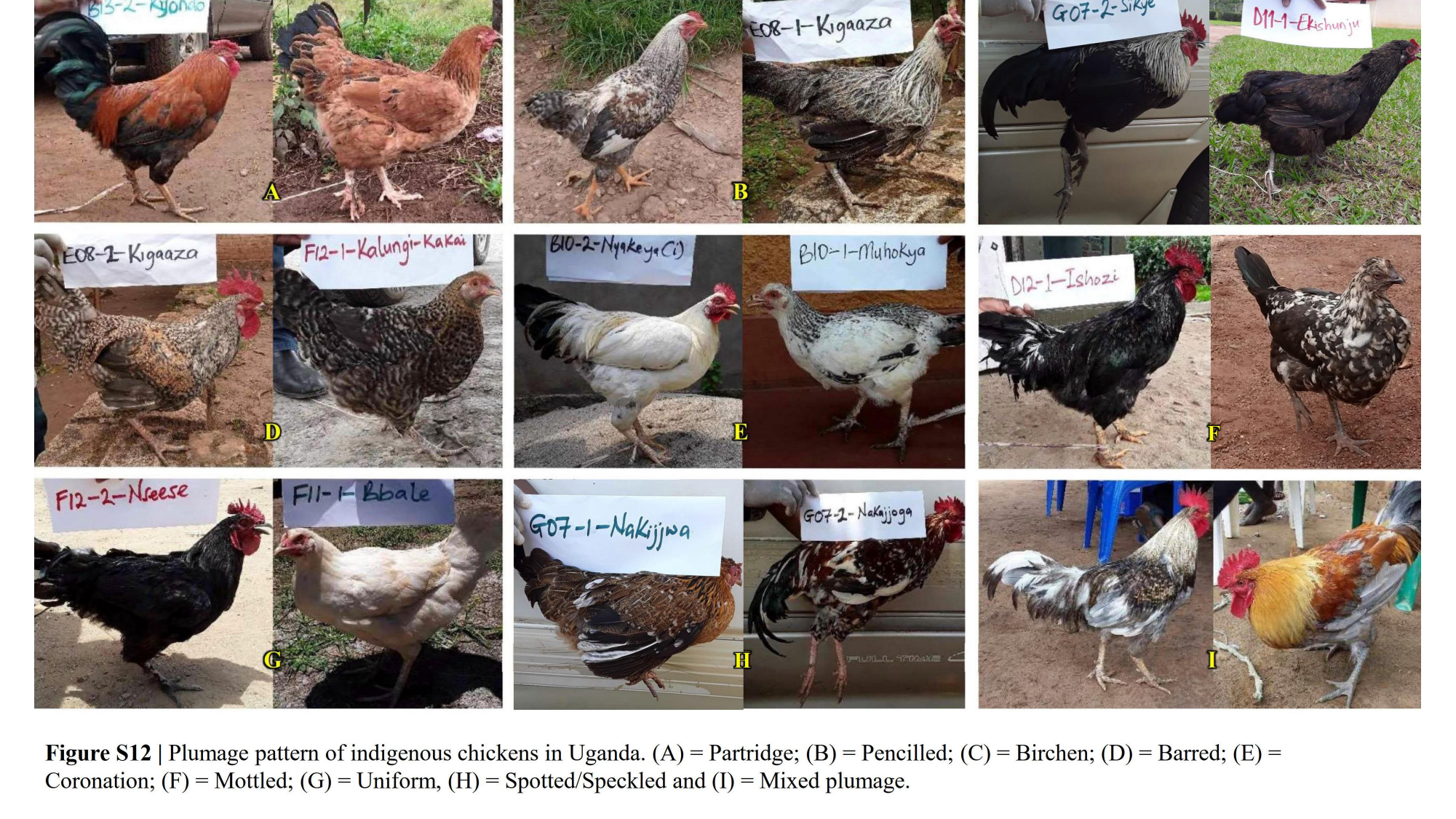

## Slide 13
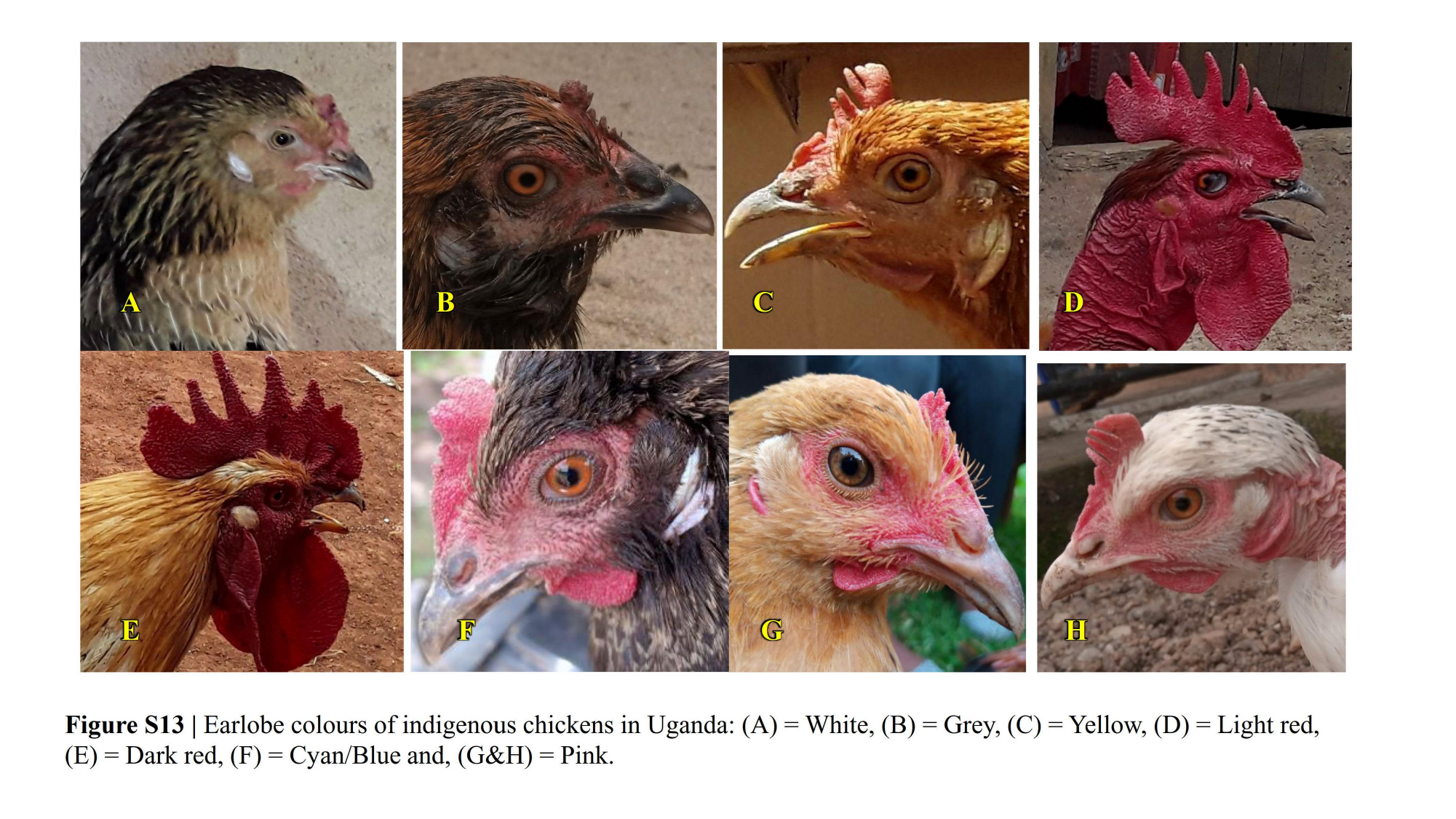

## Slide 14
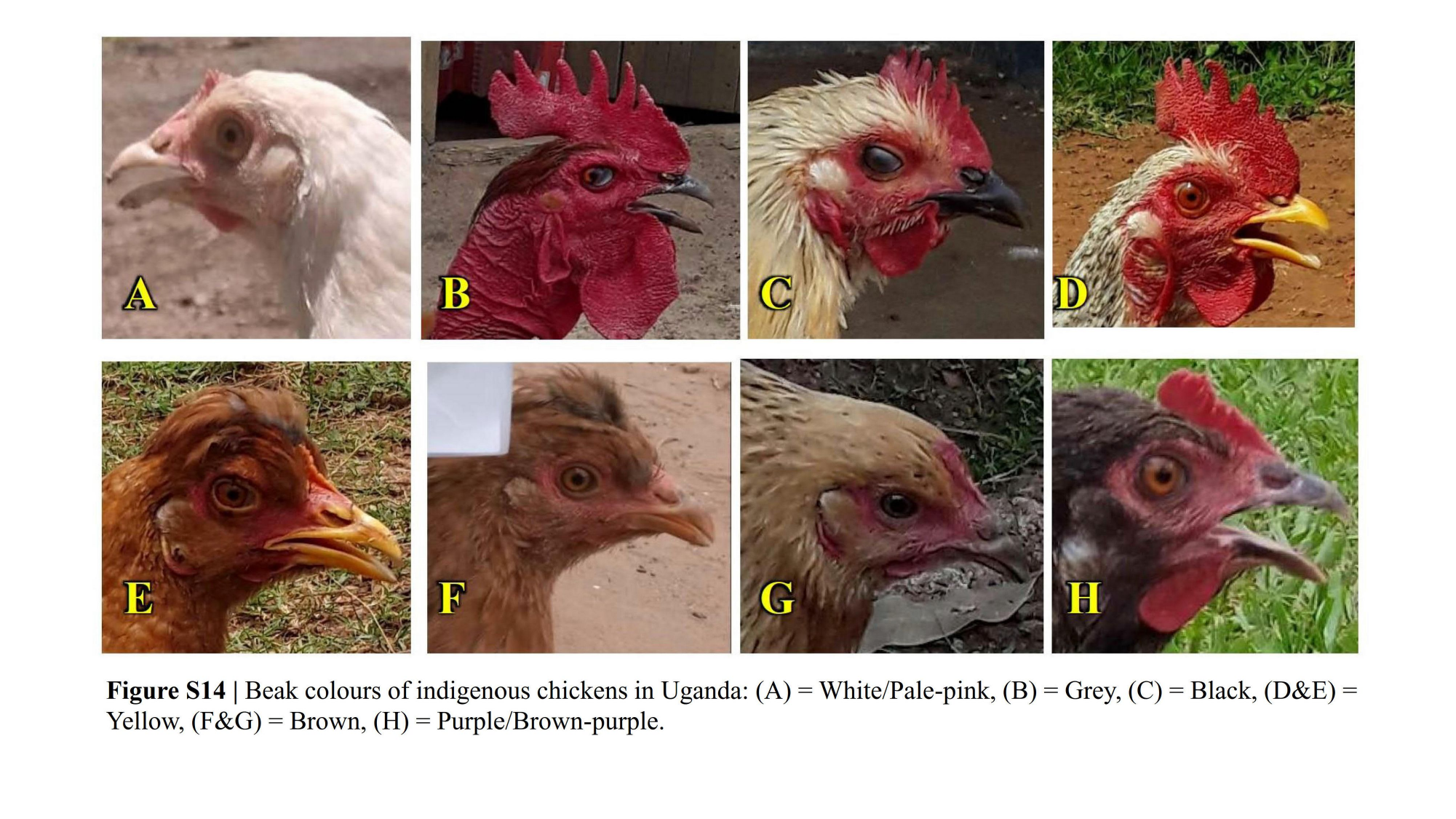

## Slide 15
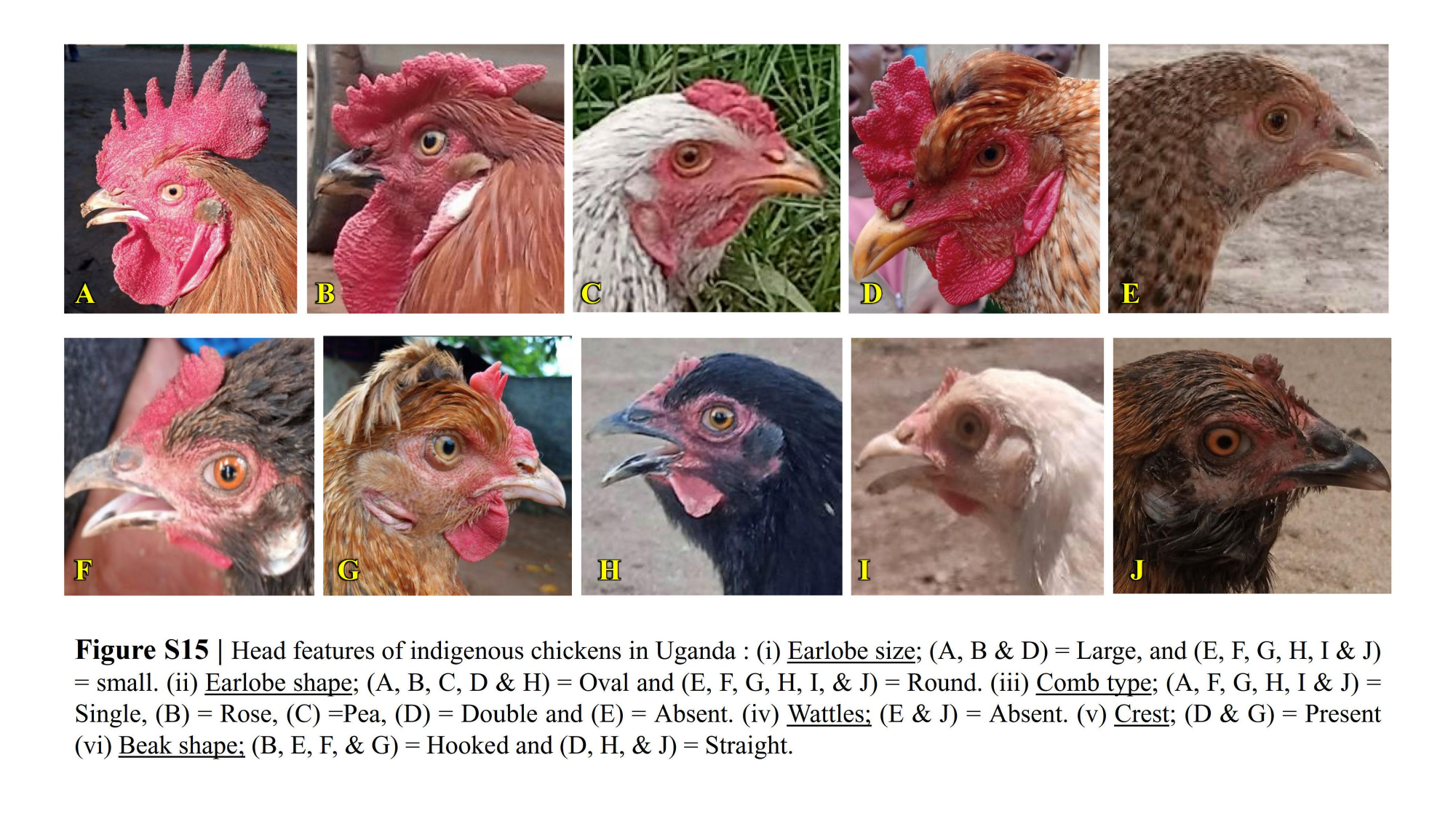

## Slide 16
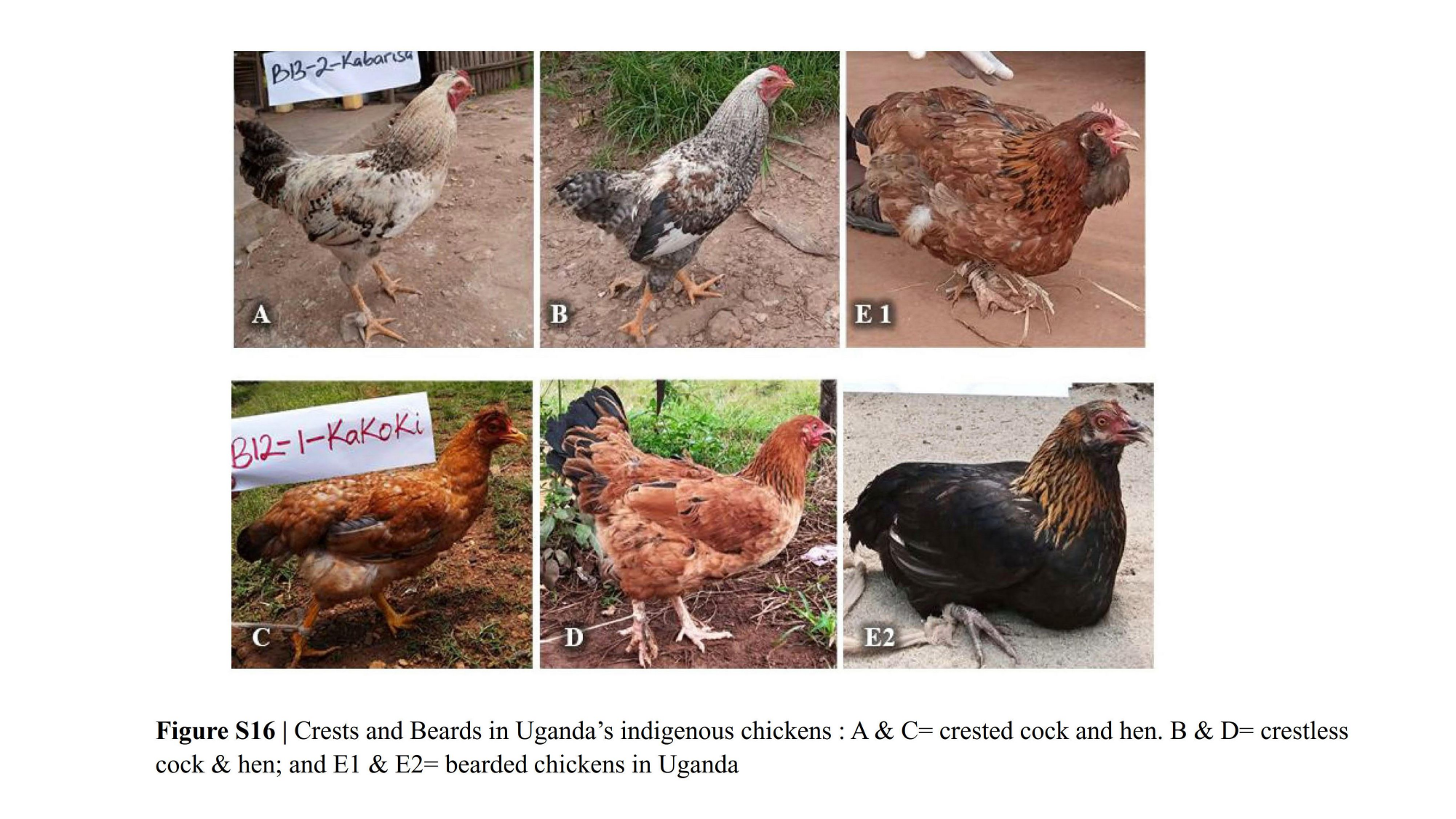

## Slide 17
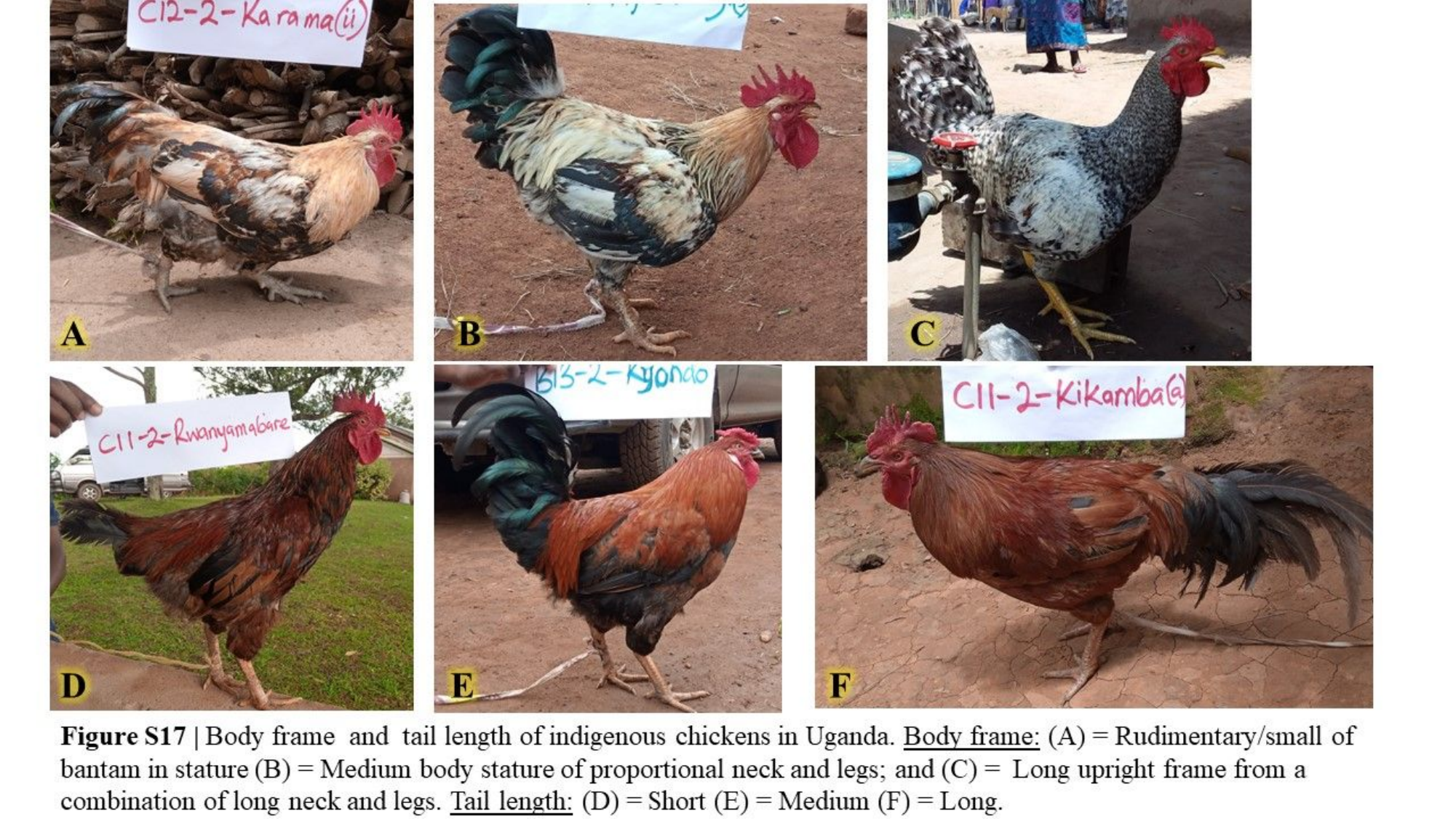

## Slide 18
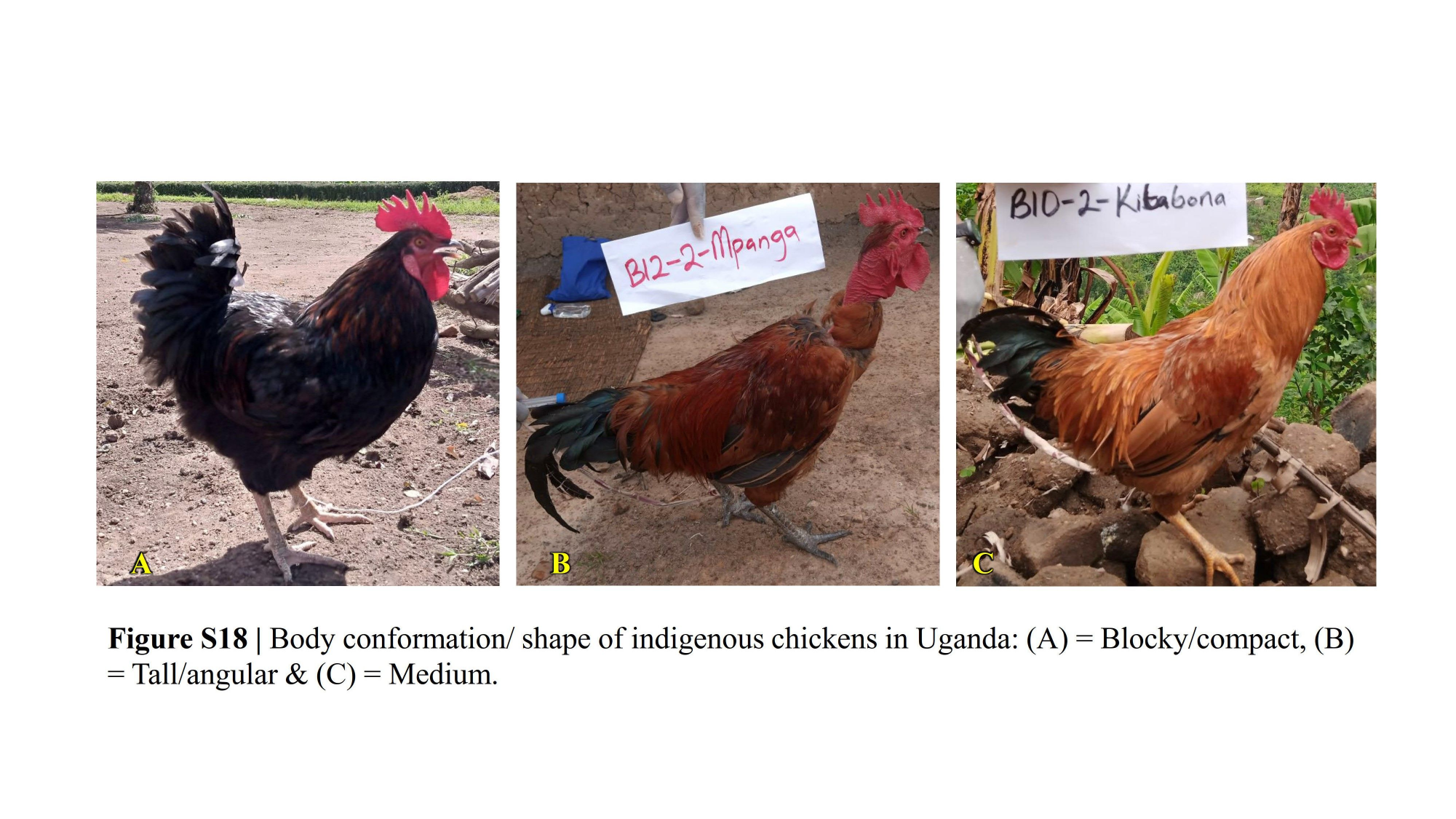

## Slide 19
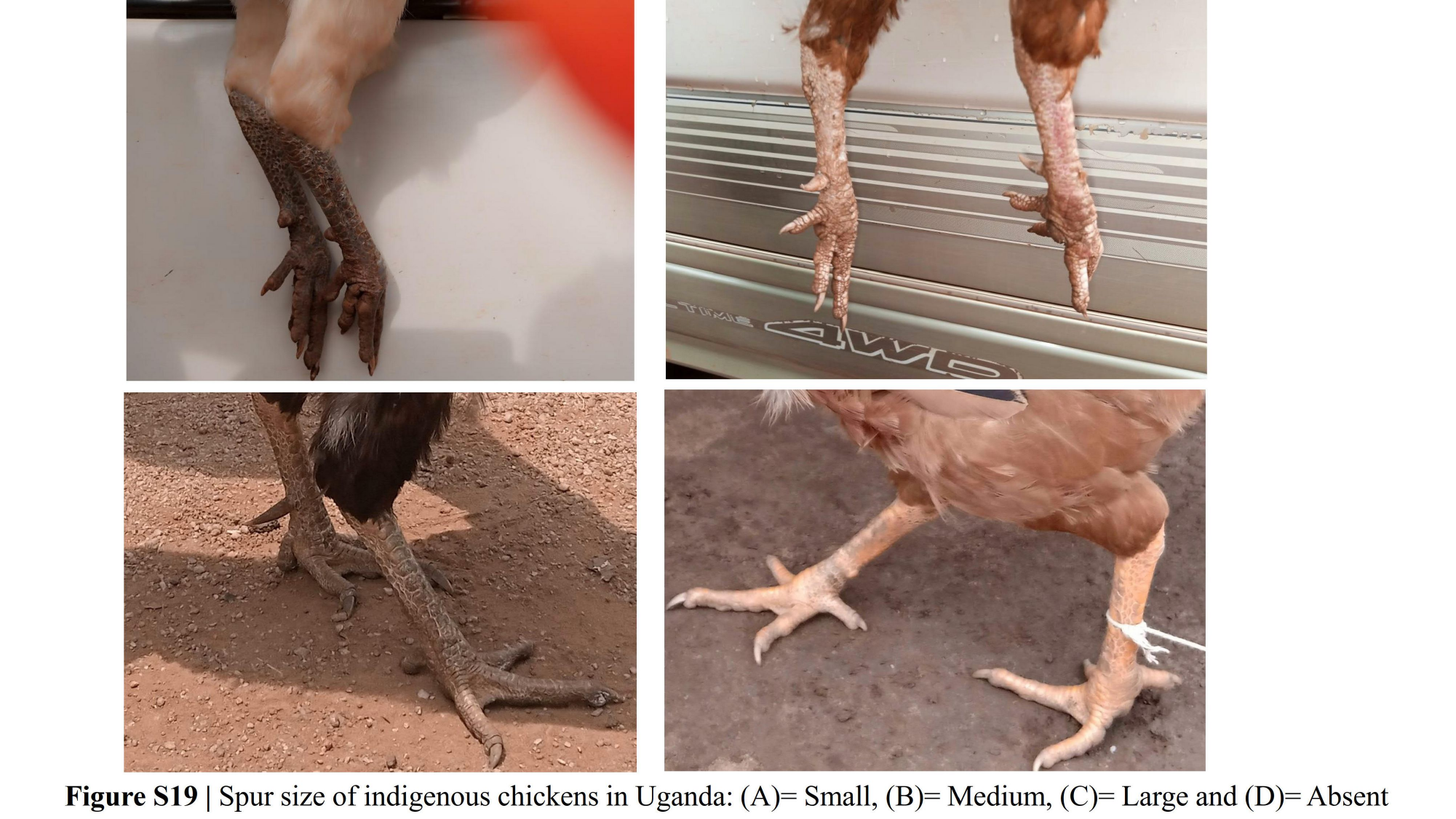

Supplement: Supplementary file 3 [file Presentation1.PPTX]
