# Supplementary material for: Uganda chicken genetic resources: I. phenotypic and production characteristics
Source: Front Genet. 2023 Jan 24;13:1033031. doi: 10.3389/fgene.2022.1033031 (PMC9902952; doi:10.3389/fgene.2022.1033031)
Supplement: Supplementary file 4 [file Table7.DOC]

**Table S7.** Pearson correlations between morphometric traits of indigenous chicken strains in Uganda

| **Trait** | **LW** | **TC** | **TL** | **SC** | **SL** | **CH** | **CL** | **WL** |
| --- | --- | --- | --- | --- | --- | --- | --- | --- |
| Live weight (LW), kg | - | 0.454** | 0.381** | 0.390** | 0.345** | 0.431** | 0.438** | 0.292** |
| Thorax circumference (TC), cm | 0.457** | - | 0.329** | -0.116* | 0.117* | 0.446** | 0.277** | 0.152** |
| Thigh length (TL), cm | **0.591**** | 0.430** | - | 0.140* | 0.304** | 0.177** | 0.125* | 0.091ns |
| Shank circumference (SC), cm | **0.648**** | 0.161** | 0.426** | - | 0.174** | 0.016ns | 0.149* | 0.213** |
| Shank length (SL), cm | **0.520**** | 0.322** | 0.416** | 0.311** | - | 0.048ns | 0.023ns | -0.069ns |
| Comb height (CH), cm | **0.551**** | 0.483** | 0.331** | 0.311** | 0.286** | - | 0.724** | 0.326** |
| Comb length (CL), cm | **0.624**** | 0.352** | 0.381** | 0.394** | 0.290** | **0.798**** | - | 0.327** |
| Wattle length (WL), cm | **0.668**** | 0.431** | 0.434** | 0.420** | 0.294** | **0.506**** | **0.598**** | - |
| ^*^*P<*0.05; ^**^*P<*0.01; ns = non-significant. Diagonal below divide for cocks (n = 281) while Diagonal above divide for hens (n = 298) | | | | | | | | |
