# Supplementary material for: Uganda chicken genetic resources: I. phenotypic and production characteristics
Source: Front Genet. 2023 Jan 24;13:1033031. doi: 10.3389/fgene.2022.1033031 (PMC9902952; doi:10.3389/fgene.2022.1033031)
Supplement: Supplementary file 5 [file Table3.DOC]

**Table S3:** Health and disease in indigenous chicken flocks across Uganda

| **Variable** | **Farmer-households [n(%)]** | | | | **Overall**  **(Uganda) n=293** | **χ²** |
| --- | --- | --- | --- | --- | --- | --- |
|  | **Northern**  **n=74** | **Central**  **n=49** | **Western**  **n=84** | **Eastern**  **n=86** |  |  |
| ***Disease frequency in the chicken flock*** | | | | | | *58.2^**^* |
| Never/ seldom | 6(8.1) | 14(28.6) | 36(42.9) | 15(17.4) | 71(24.2) |  |
| Monthly | 12(16.2) | 4(8.2) | 2(2.4) | 5(5.8) | 23(7.8) |  |
| Yearly (Seasonal) | 24(32.4) | 19(38.8) | 33(39.3) | 19(22.1) | 95(32.4) |  |
| Once in a while (randomly) | 32(43.2) | 12(24.5) | 13(15.5) | 47(54.7) | 104(35.5) |  |
| ***Chicken treatment dynamics*** | | | | | | *56.8^**^* |
| Treated or else it dies | 34(45.9) | 2(4.1) | 11(13.1) | 29(33.7) | 76(25.9) |  |
| Recover on its own | 7(9.5) | 9(18.4) | 15(17.9) | 15(17.4) | 46(15.7) |  |
| Treated only when very sick | 27(36.5) | 33(67.3) | 52(61.9) | 30(34.9) | 142(48.5) |  |
| Usually dead before treated | 3(4.1) | - | - | 8(9.3) | 11(3.8) |  |
| Never sick/Phyto-prophylaxis | 3(4.1) | 5(10.2) | 6(7.1) | 4(4.7) | 18(6.1) |  |
| Total | 74 | 49 | 84 | 86 | 293 |  |
| ***Chicken health services^1^*** | | | | | | 65.29*^**^* |
| Antihelminthic deworming | 1(5.6) | 7(38.9) | 4(22.2) | 6(33.3) | 18(6.3) |  |
| External parasites control | 2(66.7) | - | - | 1(33.3) | 3(1.0) |  |
| Vaccination | 47(36.2) | 9(6.9) | 11(8.5) | 63(48.5) | 130(45.3) |  |
| Curative treatment | 33(23.2) | 29(20.4) | 46(32.4) | 34(23.9) | 142(49.5) |  |
| None | 11(24.4) | 6(13.3) | 18(40.0) | 10(22.2) | 45(15.7) |  |
| ^**^*P<0.001*; (-) = Not reported. ^1^Chicken health services row percentages are based on the sum of responses for each category; while row total percentages are based on overall responses (i.e.; Households chicken health services are more than one type). | | | | | | |
