# Supplementary material for: Uganda chicken genetic resources: I. phenotypic and production characteristics
Source: Front Genet. 2023 Jan 24;13:1033031. doi: 10.3389/fgene.2022.1033031 (PMC9902952; doi:10.3389/fgene.2022.1033031)
Supplement: Supplementary file 6 [file Table1.DOC]

**Table S1:** Demographic profile of indigenous chickens sampled across Uganda

| **Region/subregion** | **Districts** | **Total** | **Chicken category n(%)** | | **χ²** | **P-value** |
| --- | --- | --- | --- | --- | --- | --- |
|  |  |  | **Hen** | **Cocks** |  |  |
| *Northern* | | 148 | 75(50.7) | 73(49.3) | 0.056 | 0.972 |
| Acholi | Omoro | 24 | 12 | 12 |  |  |
| Acholi | Amuru | 24 | 12 | 12 |  |  |
| Lango | Apac | 24 | 12 | 12 |  |  |
| Lango | Lira | 26 | 13 | 13 |  |  |
| West Nile | Maracha | 24 | 12 | 12 |  |  |
| West Nile | Yumbe | 26 | 13 | 13 |  |  |
| *Central* | | 97 | 49(50.5) | 48(49.5) | 0.008 | 0.929 |
| Buganda North | Mubende | 14 | 8 | 6 |  |  |
| Buganda North | Nakaseke | 14 | 7 | 7 |  |  |
| Buganda North | Nakasongola | 14 | 7 | 7 |  |  |
| Buganda North | Luwero | 13 | 7 | 6 |  |  |
| Buganda South | Rakai/Kyotera | 14 | 7 | 7 |  |  |
| Buganda South | Masaka | 14 | 7 | 7 |  |  |
| Buganda South | Ssembabule | 14 | 7 | 7 |  |  |
| *Western* |  | 168 | 88(52.4) | 80(47.6) | 0.044 | 0.998 |
| Bunyoro | Hoima | 15 | 8 | 7 |  |  |
| Bunyoro | Kiryandongo | 14 | 7 | 7 |  |  |
| Bunyoro | Kibaale | 14 | 7 | 7 |  |  |
| Tooro | Kabarole | 14 | 8 | 6 |  |  |
| Tooro | Ntoroko | 14 | 7 | 7 |  |  |
| Tooro | Kasese | 14 | 7 | 7 |  |  |
| Kigezi | Kabale, | 14 | 8 | 6 |  |  |
| Kigezi | Rukungiri | 14 | 7 | 7 |  |  |
| Ankole | Mbarara | 15 | 7 | 8 |  |  |
| Ankole | Kiruhura | 12 | 6 | 6 |  |  |
| Ankole | Buhweju | 14 | 8 | 6 |  |  |
| Ankole | Isingiro | 14 | 7 | 7 |  |  |
| *Eastern* | | 173 | 87(50.3) | 86(49.7) | 0.006 | 0.997 |
| Busoga | Kamuli | 17 | 8 | 9 |  |  |
| Busoga | Iganga | 22 | 11 | 11 |  |  |
| Busoga | Jinja | 30 | 16 | 14 |  |  |
| Busoga | Bugiri | 18 | 9 | 9 |  |  |
| Teso-Bukedi | Serere | 16 | 8 | 8 |  |  |
| Teso-Bukedi | Amuria | 16 | 8 | 8 |  |  |
| Teso-Bukedi | Kumi | 14 | 7 | 7 |  |  |
| Teso-Bukedi | Budaka | 8 | 4 | 4 |  |  |
| Elgon | Kapchorwa | 16 | 8 | 8 |  |  |
| Elgon | Mbale | 16 | 8 | 8 |  |  |
| *Total* | *35* | *586* | *298* | *288* |  |  |
| Regions are clustered by district groups of sub-regions. χ² = Chi-squared test (0.178) of *P*-value 0.981 for the overall frequency of the hen/cock chicken categories sampled. | | | | | | |

| **Agro-Ecological Zone AEZ** | **Annual rainfall (mm)** | **Altitude**  **(m ASL)** | **Annual temperature (°C)** |
| --- | --- | --- | --- |
| North Eastern Drylands NED | 745 | 351 – 1,524 | 12 – 33 |
| North Eastern Savannah Grasslands NESG | 1,197 | 975 – 1,524 | 15 - 33 |
| North Western Savannah Grasslands NWSG | 1340 | 351 – 1,341 | 15 - 25 |
| Para-Savannah Grasslands PS | 1,259 | 351 – 1,341 | 18 – 33 |
| Kyoga Plains KP | 1,200 – 1,450 | 914 – 1,800 | 15 – 33 |
| Lake Victoria Crescent LVC | 1,200 – 1,450 | 1,000 – 1,800 | 15 – 30 |
| Western Savannah Grasslands WSG | 1,270 | 621 – 1,585 | 15 – 30 |
| Pastoral Rangelands PR | 1,270 | 129 – 1,524 | 13 – 30 |
| South Western Farmlands SWF | 1,120 – 1,223 | 129 – 1,524 | 13 – 30 |
| Western Highland Ranges WHR | 1,400 | 1,299 – 3,962 | 8 – 28 |
|  |  |  |  |

Adopted from MAAIF (2010).
