# Supplementary material for: Uganda chicken genetic resources: I. phenotypic and production characteristics
Source: Front Genet. 2023 Jan 24;13:1033031. doi: 10.3389/fgene.2022.1033031 (PMC9902952; doi:10.3389/fgene.2022.1033031)
Supplement: Supplementary file 7 [file Table5.DOC]

**Table S5:** Variations in body colours of indigenous chickens in Uganda

| **Qualitative traits**  **[n (%)]** | **Northern** | | **Central** | | | **Western** | | **Eastern** | | **Uganda** | | *χ^2^* | |
| --- | --- | --- | --- | --- | --- | --- | --- | --- | --- | --- | --- | --- | --- |
|  | ***Hen***  n=75 | ***Cock***  n=73 | ***Hen***  n=58 | ***Cock***  n=54 | | ***Hen***  n=78 | ***Cock***  n=75 | ***Hen***  n=87 | ***Cock***  n=86 | ***Hen***  n=***298*** | ***Cock***  n=***288*** | ***Sex*** | ***Region*** |
| ***Skin colour*** | | | | |  | | | | | | | 1.6^ns^ | 135.4^***^ |
| White ^a^ | 26(34.7) | 36(49.3) | 41(70.7) | 31(57.4) | | 36(46.2) | 33(44.0) | 11(12.6) | 22(25.6) | 114(38.3) | 122(42.4) |  |  |
| Yellow ^b^ | 6(8.0) | 4(5.5) | 6(10.3) | 10(18.5) | | 22(28.2) | 21(28.0) | 4(4.6) | 5(5.8) | 38(12.8) | 40(13.9) |  |  |
| Dark | 43(57.3) | 33(45.2) | 11(19.0) | 13(24.1) | | 20(25.6) | 21(28.0) | 72(82.8) | 59(68.6) | **146(49.0)** | **126(43.8)** |  |  |
| ***Dominant shank colour*** | | | | | | | | | | | | *29.6^***^* | 77.3^***^ |
| White | 3(4.0) | 4(5.5) | 8(13.8) | 7(13.0) | | 13(16.7) | 15(20.0) | 9(10.3) | 15(17.4) | 33(11.1) | 41(14.2) |  |  |
| Grey/Blue-grey | 24(32.0) | 22(30.1) | 8(13.8) | 6(11.1) | | 7(9.0) | 5(6.7) | 31(35.6) | 9(10.5) | 70(23.5) | 42(14.6) |  |  |
| Black/ dark-grey | 15(20.0) | 6(8.2) | 15(25.9) | 9(16.7) | | 24(30.8) | 8(10.7) | 5(5.7) | 4(4.7) | 59(19.8) | 27(9.4) |  |  |
| Yellow | 18(24.0) | 25(34.2) | 14(24.1) | 17(31.5) | | 25(32.1) | 36(48.0) | 29(33.3) | 39(45.3) | **86(28.9)** | **117(40.6)** |  |  |
| Orange | NR | NR | 2(3.4) | 1(1.9) | | NR | 3(4.0) | 1(1.1) | 4(4.7) | 3(1.0) | 8(2.8) |  |  |
| Brown | 8(10.7) | 2(2.7) | 1(1.7) | 2(3.7) | | 2(2.6) | 1(1.3) | 2(2.3) | 3(3.5) | 13(4.4) | 8(2.8) |  |  |
| Green | 3(4.0) | 7(9.6) | 5(8.6) | 6(11.1) | | 3(3.8) | 1(1.3) | 6(6.9) | 6(7.0) | 17(5.7) | 20(6.9) |  |  |
| Pink | 4(5.3) | 7(9.6) | 5(8.6) | 6(11.1) | | 4(5.1) | 6(8.0) | 4(4.6) | 6(7.0) | 17(5.7) | 25(8.7) |  |  |
| ***Colour of eyes (Iris colour)*** | | | | | | | | | | |  | 10.4^ns^ | 200.4^***^ |
| Black | NR | NR | NR | NR | | 1(1.3) | NR | NR | NR | 1(0.3) | NR |  |  |
| Yellow | 7(9.3) | 17(23.3) | 21(36.2) | 20(37.0) | | 11(14.1) | 17(22.7) | 19(21.8) | 17(19.8) | 58(19.5) | 71(24.7) |  |  |
| Orange | 19(25.3) | 16(21.9) | 21(36.2) | 22(40.7) | | 53(67.9) | 52(69.3) | 11(12.6) | 21(24.4) | 104(34.9) | 111(38.5) |  |  |
| Red | 1(1.3) | 1(1.4) | NR | 2(3.7) | | NR | NR | 1(1.1) | NR | 2(0.7) | 3(1.0) |  |  |
| Brown | 45(60.0) | 36(49.3) | 9(15.5) | 8(14.8) | | 3(3.8) | 2(2.7) | 46(52.9) | 40(46.5) | 103(34.6) | 86(29.9) |  |  |
| Dark-green | 3(4.0) | 1(1.4) | 4(6.9) | 1(1.9) | | 6(7.7) | 1(1.3) | 7(8.0) | 5(5.8) | 20(6.7) | 8(2.8) |  |  |
| Cyan/Blue | NR | 1(1.4) | NR | NR | | 1(1.3) | NR | NR | 1(1.2) | 1(0.3) | 2(0.7) |  |  |
| Pink | NR | 1(1.4) | 3(5.2) | 1(1.9) | | 3(3.8) | 3(4.0) | 3(3.4) | 2(2.3) | 9(3.0) | 7(2.4) |  |  |
| **P<0.05; **P<0.01; ***P<0.001; ns = non-significant; χ^2^ = Chi-square test of fixed variables;* n = *Chickens sampled; NR = not reported*  ^a.^ Including greyish-white and grey-pink/light-pink.  ^b.^ Including orange/pink-orange  ^c.^ Including purplish-brown | | | | | | | | | | | | | |

**Table S4 continued:** Variations in body colours of indigenous chickens in Uganda

| **Qualitative traits**  **[n (%)]** | **Northern** | | **Central** | | **Western** | | **Eastern** | | **Uganda** | | *χ^2^* | |
| --- | --- | --- | --- | --- | --- | --- | --- | --- | --- | --- | --- | --- |
|  | ***Hen***  n=75 | ***Cock***  n=73 | ***Hen***  n=58 | ***Cock***  n=54 | ***Hen***  n=78 | ***Cock***  n=75 | ***Hen***  n=87 | ***Cock***  n=86 | ***Hen***  n=***298*** | ***Cock***  n=***288*** | ***Sex*** | ***Region*** |
| ***Colour of earlobe*** | | | | | | | | | | | 190.0^***^ | 130.1^***^ |
| White | 2(2.7) | 1(1.4) | 4(6.9) | 4(7.4) | 22(28.2) | 8(10.7) | 1(1.1) | 1(1.2) | 29(9.7) | 14(4.9) |  |  |
| Grey | 3(4.0) | 1(1.4) | 2(3.4) | 2(3.7) | 3(3.8) | NR | 1(1.1) | NR | 9(3.0) | 3(1.0) |  |  |
| Yellow | 15(20.0) | 4(5.5) | 12(20.7) | 6(11.1) | 14(17.9) | 6(8.0) | 4(4.6) | 3(3.5) | **45(15.1)** | 19(6.6) |  |  |
| Light red | 7(9.3) | 45(61.6) | 6(10.3) | 32(59.3) | 13(16.7) | 56(74.7) | 24(27.6) | 69(80.2) | **50(16.8)** | **202(70.1)** |  |  |
| Dark red | 14(18.7) | 21(28.8) | 17(29.3) | 5(9.3) | 5(6.4) | 1(1.3) | 25(28.7) | 11(12.8) | **61(20.5)** | **38(13.2)** |  |  |
| Green | 2(2.7) | 1(1.4) | NR | 1(1.9) | 1(1.3) | 1(1.3) | 6(6.9) | NR | 9(3.0) | 3(1.0) |  |  |
| Cyan/Blue | 4(5.3) | NR | 6(10.3) | 1(1.9) | 15(19.2) | 3(4.0) | 7(8.0) | NR | 32(10.7) | 4(1.4) |  |  |
| Pink | 28(37.3) | NR | 11(19.0) | 3(5.6) | 5(6.4) | NR | 19(21.8) | 2(2.3) | **63(21.1)** | 5(1.7) |  |  |
|  |  |  |  |  |  |  |  |  |  |  |  |  |
| ***Colour of beak*** | | | | | | | | | | | 40.3^***^ | 145.1^***^ |
| White/pale pink | 4(5.3) | 6(8.2) | 5(8.6) | 5(9.3) | 6(7.7) | 6(8.0) | 9(10.3) | 5(5.8) | 24(8.1) | 22(7.6) |  |  |
| Grey | 18(24.0) | 34(46.6) | 18(31.0) | 23(42.6) | 21(26.9) | 27(36.0) | 1(1.1) | 7(8.1) | **58(19.5)** | **91(31.6)** |  |  |
| Black | 1(1.3) | 3(4.1) | 1(1.7) | 2(3.7) | NR | 1(1.3) | 6(6.9) | 22(25.6) | 8(2.7) | 28(9.7) |  |  |
| Yellow | 22(29.3) | 14(19.2) | 9(15.5) | 9(16.7) | 11(14.1) | 18(24.0) | 22(25.3) | 12(14.0) | **64(21.5)** | **53(18.4)** |  |  |
| Brown | 14(18.7) | 6(8.2) | 10(17.2) | 7(13.0) | 8(10.3) | 9(12.0) | 32(36.8) | 32(37.2) | **64(21.5)** | **54(18.8)** |  |  |
| Dark-green | 4(5.3) | 4(5.5) | 2(3.4) | 5(9.3) | 6(7.7) | 8(10.7) | 12(13.8) | 5(5.8) | 24(8.1) | 22(7.6) |  |  |
| Purple/brown-purple | 16(16.0) | 6(8.2) | 13(22.4) | 3(5.6) | 24(30.8) | 6(8.0) | 5(5.7) | 3(3.5) | **54(18.1)** | 18(6.2) |  |  |
| Cyan/Blue | NR | NR | NR | NR | 2(2.6) | NR | NR | NR | 2(0.7) | NR |  |  |
| **P<0.05; **P<0.01; ***P<0.001; ns = non-significant; X^2^ = Chi – square test of fixed variables;* n = *Chickens sampled; NR = not reported* | | | | | | | | | | | | |
