# Supplementary material for: Uganda chicken genetic resources: I. phenotypic and production characteristics
Source: Front Genet. 2023 Jan 24;13:1033031. doi: 10.3389/fgene.2022.1033031 (PMC9902952; doi:10.3389/fgene.2022.1033031)
Supplement: Supplementary file 8 [file Table2.DOCX]

**Table S*2*:** Summary description of the 10 AEZs of Uganda

| **Agro-Ecological Zone (AEZ)** | **Annual rainfall (mm)** | **Altitude**  **(m ASL)** | **Annual temperature (℃)** |  |
| --- | --- | --- | --- | --- |
| North Eastern Drylands | 745 | 351 – 1,524 | 12 – 33 |  |
| North Eastern Savannah Grasslands | 1,197 | 975 – 1,524 | 15 - 33 |  |
| North Western Savannah Grasslands | 1340 | 351 – 1,341 | 15 - 25 |  |
| Para-Savannah Grasslands | 1,259 | 351 – 1,341 | 18 – 33 |  |
| Kyoga Plains | 1,200 – 1,450 | 914 – 1,800 | 15 – 33 |  |
| Lake Victoria Crescent | 1,200 – 1,450 | 1,000 – 1,800 | 15 – 30 |  |
| Western Savannah Grasslands | 1,270 | 621 – 1,585 | 15 – 30 |  |
| Pastoral Rangelands | 1,270 | 129 – 1,524 | 13 – 30 |  |
| South Western Farmlands | 1,120 – 1,223 | 129 – 1,524 | 13 – 30 |  |
| Western Highland Ranges | 1,400 | 1,299 – 3,962 | 08 – 28 |  |
| ASL = Above sea level. Adopted from MAAIF (2010). | | | | |
