# Supplementary material for: Uganda chicken genetic resources: I. phenotypic and production characteristics
Source: Front Genet. 2023 Jan 24;13:1033031. doi: 10.3389/fgene.2022.1033031 (PMC9902952; doi:10.3389/fgene.2022.1033031)
Supplement: Supplementary file 9 [file Table4.DOCX]

**Table S4:** Variations in feather morphology, distribution and plumage patterns of indigenous chickens in Uganda

| **Qualitative traits**  **[n (%)]** | **Northern** | | **Central** | | **Western** | | **Eastern** | | **Uganda** | | *χ^2^* | |
| --- | --- | --- | --- | --- | --- | --- | --- | --- | --- | --- | --- | --- |
|  | **Hen**  **n=75** | **Cock**  **n=73** | **Hen**  **n=58** | **Cock**  **n=54** | **Hen**  **n=78** | **Cock**  **n=75** | **Hen**  **n=87** | **Cock**  **n=86** | **Hen**  **n=298** | **Cock**  **n=288** | **Sex** | **Region** |
| ***Feather structure*** | | | | | | | | | | | *3.6^ns^* | *29.2^**^* |
| Smooth (Neat plane) | 74(98.7) | 72(98.6) | 56(96.6) | 45(83.3) | 76(97.4) | 74(98.7) | 85(97.7) | 84(97.7) | 291(97.7) | 275(95.5) |  |  |
| Frizzled | 1(1.3) | 1(1.4) | 1(1.7) | 2(3.7) | NR | 1(1.3) | 2(2.3) | 2(2.3) | 4(1.3) | 6(2.1) |  |  |
| Silky | NR | NR | 1(1.7) | 5(9.3) | NR | NR | NR | NR | 1(0.3) | 5(1.7) |  |  |
| Superficially silky | NR | NR | NR | 2(3.7) | 2(2.6) | NR | NR | NR | 2(0.7) | 2(0.7) |  |  |
|  |  |  |  |  |  |  |  |  |  |  |  |  |
| ***Feather distribution*** | | | | | | | | | | | *0.9^ns^* | *14.5^*^* |
| Naked neck | 9(12.0) | 6(8.2) | 2(3.4) | NR | 4(5.1) | 3(4.0) | 4(4.6) | 8(9.3) | 19(6.4) | 17(5.9) |  |  |
| Normal feathered (Full) | 65(86.7) | 65(89.0) | 56(96.6) | 52(96.3) | 71(91.0) | 67(89.3) | 77(88.5) | 73(84.9) | 269(90.3) | 257(89.2) |  |  |
| Feathered shank (Ptilopody) | 1(1.3) | 2(2.7) | NR | 2(3.7) | 3(3.8) | 5(6.7) | 6(6.9) | 5(5.8) | 10(3.4) | 14(4.9) |  |  |
|  |  |  |  |  |  |  |  |  |  |  |  |  |
| ***Body plumage pattern*** | | | | | | | | | | | *176.7^***^* | *262.8^***^* |
| Partridge | 1(1.3) | 22(30.1) | 15(25.9) | 24(44.4) | 15(19.2) | 33(44.0) | 1(1.1) | 30(34.9) | 32(10.7) | 109(37.8) |  |  |
| Pencilled | 9(12.0) | 1(1.4) | 18(31.0) | 2(3.7) | 36(46.2) | 4(5.3) | 1(1.1) | 3(3.5) | 64(21.5) | 10(3.5) |  |  |
| Birchen | 6(8.0) | 29(39.7) | NR | 10(18.5) | NR | 8(10.7) | NR | 10(11.6) | 6(2.0) | 57(19.8) |  |  |
| Barred | 8(10.7) | 4(5.5) | 2(3.4) | NR | 1(1.3) | 3(4.0) | 2(2.3) | 10(11.6) | 13(4.4) | 17(5.9) |  |  |
| Uniform (solid-coloured) | 26(34.7) | 6(8.2) | 14(24.1) | 8(14.8) | 17(21.8) | 7(9.3) | 12(13.8) | 2(2.3) | 69(23.2) | 23(8.0) |  |  |
| Coronation | 6(8.0) | 3(4.1) | 3(5.2) | 4(7.4) | 4(5.1) | 8(10.7) | 3(3.4) | 9(10.5) | 16(5.4) | 23(8.3) |  |  |
| Mottled | 4(5.3) | 1(1.4) | 3(5.2) | 5(9.3) | NR | 9(12.0) | 2(2.3) | 1(1.2) | 9(3.0) | 16(5.6) |  |  |
| Spotted/speckled | 3(4.0) | NR | 3(5.2) | 1(1.9) | 5(6.4) | 3(4.0) | 3(3.4) | NR | 14(4.7) | 4(1.4) |  |  |
| None (Mixed pattern) | 12(16.0) | 7(9.6) | NR | NR | NR | NR | 63(72.4) | 21(24.4) | 75(25.2) | 28(9.7) |  |  |
| **P<0.05; **P<0.01; ***P<0.001; ns = non-significant; χ^2^ = Chi – square test of fixed variables;* n = *Chickens sampled; NR = not reported* | | | | | | | | | | | | |
